# Unravelling the genetic structure and genetic relationships of indigenous Greek sheep breeds using SNP markers

**DOI:** 10.1186/s12711-026-01074-5

**Published:** 2026-08-03

**Authors:** Spyridoula Georgatou, Dimitris Papachristou, George Laliotis, Nicolaos Kassinis, Boro Mioč, Aashish Gyawali, Doris Seichter, Maulik Upadhyay, Nikolaos Kostaras, Ivica Medugorac, Iosif Bizelis, Panagiota Koutsouli

**Affiliations:** 1 Laboratory of Animal Breeding and Husbandry, Department of Animal Science, Agricultural University of Athens, 75 Iera Odos, 11855 Athens, Greece; 2 Game and Fauna Service, Ministry of Interior, Nicosia, Cyprus; 3 Department of Animal Science and Technology, Faculty of Agriculture, University of Zagreb, Svetošimunska cesta 25, 10000 Zagreb, Croatia; 4 Tierzuchtforschung e.V. München, 85586 Grub, Germany; 5 Population Genomics Group, Department of Veterinary Sciences, LMU Munich, 82152 Martinsried, Germany; 6 SNSB-Genomics Core Facility, Menzinger Straße 67, 80638 Munich, Germany; 7 AMALTHIA, Network for the Protection of Greek Indigenous Farm Animals, 51 Argyrokastrou, 15669 Athens, Greece

## Abstract

**Background:**

Indigenous sheep of Greece represent important genetic resources shaped by long-term adaptation to diverse environments. However, many populations face demographic decline, genetic erosion, and their genomic diversity remains inadequately characterized. This study presents a comprehensive genome-wide analysis of both recognized and previously uncharacterized indigenous Greek sheep. Newly genotyped data from 36 Greek breeds/populations, one Cypriot breed and one outgroup (Cypriot Mouflon) were combined with previously published genotypes from 85 international breeds and four additional outgroups, analyzing 127 ovine populations in total. To mitigate commercial BeadChip ascertainment bias when evaluating these uncharacterized populations, we utilized genome-wide SNP blocks to assess genetic diversity, reconstruct population structure, estimate effective population sizes, and place them within a broad comparative framework encompassing European, Southwest Asian, and North African breeds.

**Results:**

Genome-wide analyses evaluating 46,733 SNPs and 4347 multi-allelic haplotype blocks revealed a distinct ascertainment bias affecting Western and Eastern breeds differently. Mitigating this bias via the block-based approach demonstrated that Greek sheep breeds/populations retain high genetic diversity, despite pronounced heterogeneity. Breeds such as Lesvos, Vlahiko, and Karagouniko exhibited high heterozygosity, low inbreeding, and relatively large effective population sizes, whereas Thraki, Agrinio, Katafygio, Serres, and Argos showed low diversity, elevated inbreeding, and small recent effective population sizes. Greek breeds/populations occupied an intermediate position between Western European and Southwest Asian groups, reflecting their geographic location and historical role in early dispersal routes. Island populations (Cretan breeds, Kasos and Karpathos) formed a cohesive genetic cluster shaped by long-term isolation, while semi-fat-tailed Greek breeds showed close affinities with Middle Eastern and North African populations. Introgression from East Friesian sheep strongly influenced the genomic profile of the Arta breed.

**Conclusions:**

This study provides a comprehensive genomic baseline for indigenous Greek and Cypriot sheep, highlighting their high genetic diversity and complex demographic histories. While several breeds represent valuable reservoirs of adaptive variation, others face immediate risk of genetic erosion. These findings provide essential guidance for prioritizing conservation actions and integrating genomic information into sustainable management and breeding strategies.

**Supplementary Information:**

The online version contains supplementary material available at 10.1186/s12711-026-01074-5.

## Background

The domestication of sheep (*Ovis aries*) is widely regarded as a critical milestone in the emergence of agricultural societies in the Mediterranean Basin. Excavations and zooarchaeological analyses [1, 2] show that the domestication of sheep began in the so-called Fertile Crescent, more precisely in the northern Levant, and then spread to other regions. Zeder [3] and Demirci et al. [4] also posit that the domestication of sheep commenced as early as 11–12,000 BCE, first occurred within the Fertile Crescent, particularly in southeastern Anatolia and the northern Zagros Mountains. The introduction of sheep to the island of Cyprus is attributed to the early farmers who migrated from the Fertile Crescent, marking the first significant westward migration around 10,500 BCE [3, 5–7]. Furthermore, the presence of archaeological evidence from sites such as Frachthi Cave suggests that sheep were already an integral part of the Neolithic farming in Greece, by around 8000 BCE [8]. The movement of domesticated sheep was facilitated by two primary routes: an overland route crossing through western Anatolia and the Hellespont into the Balkans, and an Aegean maritime route [9–11]. As Cymbron et al. [12] discussed, based on Tresset & Vigne [13], the expansion of farming and livestock proceeded more rapidly along the Mediterranean coast. This phenomenon was attributed to several factors, including seafaring, forest density and the relationship between the native Mesolithic and immigrant Neolithic human populations. In older times, sheep were transported across the Aegean to Greece and, into southern Italy, Sicily, and Spain [14]. Recent genomic analyses support that Greece played a pivotal role as a critical stepping stone in this process, as seafaring Neolithic settlers carried livestock and agricultural practices across the Mediterranean [3, 15]. In Europe, where there were no native wild sheep at that time, the only plausible source of introgression comes from early domestic animals that went feral—i.e., European mouflon—and later back-admixed into managed flocks [15]. By the end of the Neolithic period, domestic sheep had proliferated throughout Europe, following both coastal maritime routes and overland paths via the Danube River, reaching the British Isles and Scandinavia 2000–3000 years later [14].

Genetic studies have provided strong evidence that European domestic sheep are descended from Near Eastern ancestors. Bruford and Townsend [16] and Luikart et al. [17] confirmed that domesticated sheep originated from the Asian mouflon (*Ovis orientalis*), which spread across Europe during the Neolithic agricultural expansion. Recent analyses [18–20] further support this view, highlighting the role of Near Eastern domesticated lineages in the ancestry of modern European breeds. Although the European mouflon (*Ovis gmelinii*) is closely related to domestic sheep, it is now recognized as a distinct species representing a later feralization event—a reversion to a wild state from domesticated ancestors—rather than a true wild progenitor of domestic sheep. The first zooarchaeological evidence for this feral origin was provided by Vigne [21], who demonstrated that mouflons were introduced to the Mediterranean islands during the Neolithic period as domestic animals, later reverting to a wild phenotype. Since then, this hypothesis has been corroborated by genetic and historical studies [16, 17].

References on Greek indigenous sheep breeds have been available since the early twentieth century, starting with Dimitriadis [22] in his work ‘Greek Animal Husbandry’, which marks the first comprehensive study on Greek livestock. The diversity of autochthonous Greek sheep breeds reflects the long history of sheep farming in Greece, from domestication until the present [23]. Each breed possesses distinct phenotypic and productive characteristics due to the long-term pressure of natural and artificial selection [24], leading to the unique characteristics of native sheep breeds, including disease resistance, resilience to changing weather conditions, and the ability to survive in environments with poor vegetation and limited resources [25, 26]. Therefore, the genetic pool of these breeds contains genes that promote sustainable sheep farming practices that meet present and future needs and expectations.

Improving our comprehension of genetic diversity among and within local breeds is considered highly important for their efficient use in sustainable animal farming, particularly in challenging environments and less intensive productive systems [27] and for the successful implementation of conservation programs [28]. Many studies on the genetic diversity of sheep breeds, have been conducted with a limited subset of well-known Greek breeds by microsatellites [29–36] or by SNPs [37–40]. Also, recent studies, regarding European breeds, do not include Greek sheep breeds at all, even in a comparative context [41, 42]. The present study, examines a total of 36 distinct breeds/populations in terms of morphology, physiology and productivity [26], covering the entire Greek territory. This includes 25 officially recognized indigenous breeds, 5 breeds supported by existing literature, and 6 unrecognized autochthonous populations. While these 6 local populations lack prior scientific references, they have been identified as separate, distinct populations through personal field visits and communication with local farmers. Using standard single-nucleotide polymorphism (SNP) array technology, this study aims to comprehensively define the genetic landscape of indigenous Greek sheep breeds, and small local populations with an unclear official status, aiming to contribute to the protection and conservation of these breeds. Specifically, the objectives of the study are as follows:

- The exploration of the genomic identity and diversity of the indigenous Greek breeds and uncharacterized populations, alongside the Cyprus-fat-tailed breed and
- The description of the genetic structure, phylogenetic relationships, and admixture patterns by comparing the Greek sheep with wild sheep and diverse breeds from Europe, Asia, and North Africa.

## Methods

**Fig. 1:**
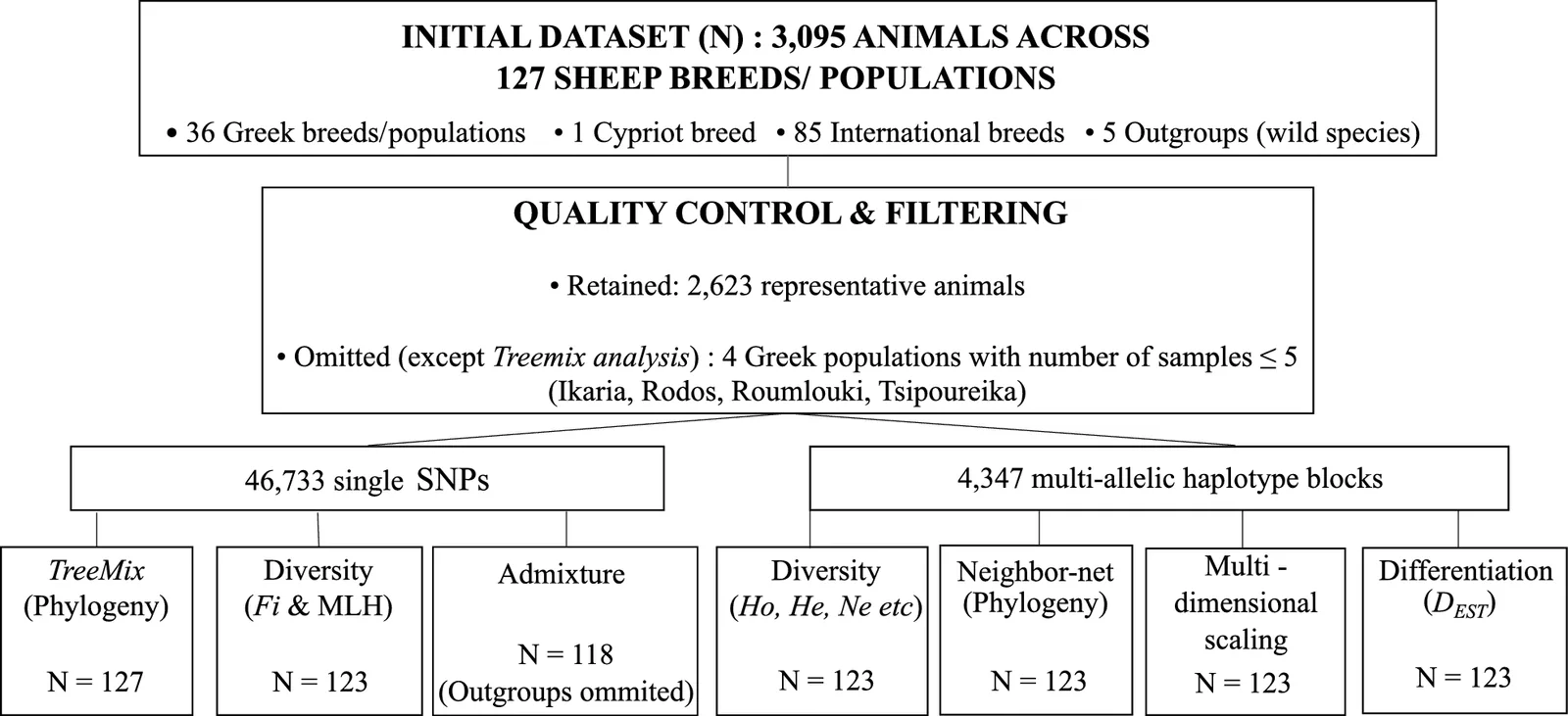
Graphical overview of the study design and dataset composition. The diagram illustrates the initial dataset composition (127 populations), followed by quality control filtering. The subsequent analytical pipeline is divided into two main branches based on the genetic marker type utilized: single-nucleotide polymorphisms (46,733 SNPs) and multi-allelic haplotype blocks (4347 blocks; utilized to mitigate ascertainment bias). The specific number of breeds (N) are detailed for each respective downstream analysis

A graphical overview of the experimental design, quality control filtering, and analytical pipelines utilized in this study is summarized in Fig. 1.

### Sampling

**Fig. 2:**
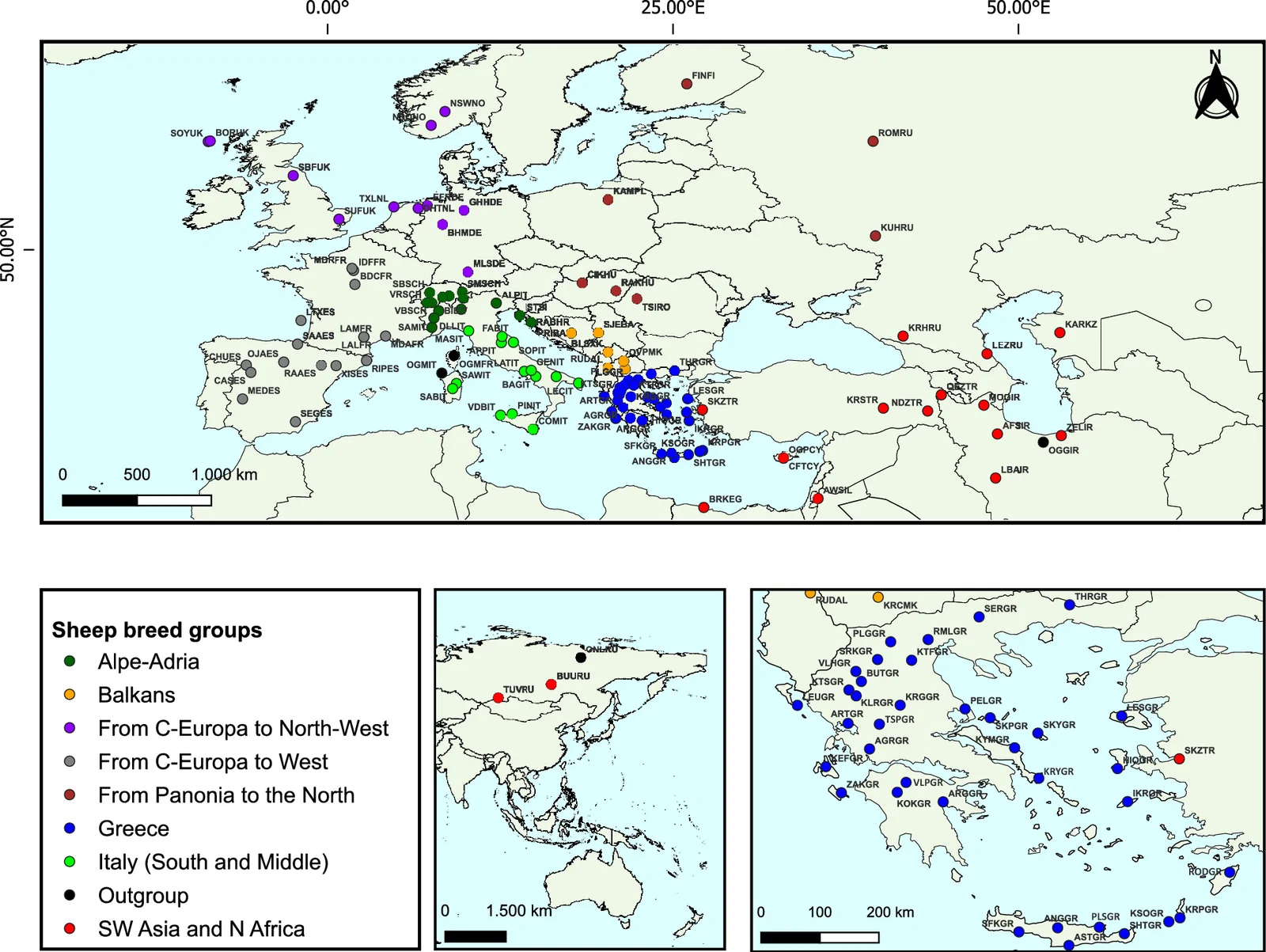
Origin of the breeds used in the analyzed dataset. C: Central, SW: Southwest, N: North, (Map created using the Free and Open Source QGIS)

Blood samples were collected from a total of 778 individuals representing 25 official recognized breeds, 5 breeds with references in the literature and 6 local populations with no available written references. These populations are acknowledged locally and identified in local visits [26]. The sampling locations are illustrated in Fig. 2. Notably, this figure, along with all subsequent figures in the study, follows a standardized color code assigned to each geographic region, as detailed in Additional file 1 Table S1. All samples were collected by trained personnel in accordance with strict veterinary standards. Ethical approval for the study was granted by the Ethics Committee of the Agricultural University of Athens (54/29.06.2023). The 36 local Greek sheep breeds sampled in our analysis are categorized as follows:

(i) Thirty recognized breeds: Agrinio/ Agriniou (AGRGR; n = 20), Anogia/ Anogeiano (ANGGR; n = 25), Argos (ARGGR; n = 31), Epirus Orino/ Boutsiko (BUTGR; n = 10), Chios (HIOGR; n = 48), Pelagonia/ Florina (PLGGR; n = 12), Arta (ARTGR; n = 14), Kalarrytiko (KLRGR; n = 31), Karagouniko (KRGGR; n = 27), Karystos/ Karystou (KRYGR; n = 29), Katafygio/ Katafygiou (KTFGR; n = 8), Katsika (KTSGR; n = 10), Kefalonia/ Kefallinias (KEFGR; n = 29), Kymi (KYMGR; n = 9), Lesvos (LESGR; n = 67), Roumlouki/ Roumloukiou (RMLGR; n = 4), Sarakatsaniko (SRKGR; n = 29), Serres/ Serrai (SERGR; n = 14), Sfakia/ Sfakiano (SFKGR; n = 35), Sitia (SHTGR; n = 19), Skopelos (SKPGR; n = 31), Pelion/ Pilioritiko (PELGR; n = 29), Thraki (THRGR; n = 14), Vlahiko (VLHGR; n = 25), Zakynthos (ZAKGR; n = 14), Asterousia (ASTGR; n = 40), Ikaria (IKRGR; n = 5), Skyros (SKYGR; n = 14), Kokovitiko (KOKGR; n = 24), and Lefkimmi (LEUGR; n = 19).
(ii) Six local populations (no previous written references): Rodos (RODGR; n = 4), Karpathos (KRPGR; n = 16), Kasos (KSOGR; n = 58), Local Lasithi Plateau (PLSGR; n = 11), Vlahiko Peloponnese (VLPGR; n = 7), and Tsipoureika (TSPGR; n = 3).

Moreover, samples were collected from the Cyprus fat-tailed breed (CFTCY; n = 20). The body measurements of the Greek sheep breeds/populations studied here are provided in Additional file 1 Table S2. Also, detailed sampling information (including the number of flocks and samples per flock), alongside recent population figures, active breeding programs, and *in situ* conservation status for the recognized breeds, are provided in Additional file 1 Table S3 and in Additional file 2 Table S4. Despite the extensive nature of this study, it is crucial to acknowledge its limitations. The sample sizes differed among the breeds, and it is possible that certain populations were still underrepresented. In some breeds, namely, the Chios, Lesvos, Kymi and Cyprus fat-tailed breeds whole genome genotypes from other studies were additionally used: For the Chios breed 22 samples and for the Cyprus fat-tailed breed 30 samples were reported by [43]. For the Lesvos breed six samples and for the Kymi breed six samples were reported by [39] (Additional file 1 Tables S1–S3). Additionally, genotypic data from 86 international breeds from diverse regions outside Greece were incorporated for comparison purposes (Additional file 1 Table S1). Their inclusion was based on genetic, historical, and geographical criteria. Some of these were sampled and genotyped in this study: Cyprus fat-tailed, Pramenka (Sjenica, Bosnia), Pramenka (Privor, Bosnia), Rab (Croatia), Racka (Hungary), Merinolandschaf (Germany). More specifically, the extensive dataset of the selected breeds (86 breeds from regions outside Greece) was categorized into seven primary geographic groups: (a) Southwest Asia and North Africa, (b) Italy, (c) the Balkans, (d) the Alpe–Adria, (e) from Pannonia to the North, (f) from Central Europe to the Northwest, and (g) from Central Europe to the West. Additionally, five outgroups were included, consisting of *Ovis nivicola* (Snow sheep, ONLRU), *Ovis orientalis musimon*—Asian Mouflon (OGGIR), *Ovis gmelini ophion*—Cyprus Mouflon (OGPCY), *Ovis gmelini musimon*—Sardinian Mouflon (OGMIT), and *Ovis gmelini musimon*—Corsican Mouflon (OGMFR). In total, 122 domestic sheep breeds/populations and 5 wild ovine outgroups were included in our dataset, forming 9 distinct groups for the analysis: the 8 geographic groups (including the Greek breeds group) and the outgroup (wild sheep). All the above breeds are described in Additional file 1 Table S1.

### DNA extraction, SNP genotyping, and quality control

DNA isolation was performed using a commercial kit (QIAamp DNA MiniKit, QIAGEN) according to the manufacturer’s instructions. For SNP genotyping, the Illumina OvineSNP50 BeadChip array was used following standard procedures (http://www.illumina.com). Furthermore, quality controls were applied to obtain a high-quality dataset: (i) only the animals with call rate higher than 0.95 were included; (ii) SNPs that mapped to unknown or sex chromosomes were removed; (iii) SNPs that were genotyped for less than 90% of the samples were removed; (iv) SNPs with a MAF lower than 0.025 and showing departures from Hardy–Weinberg equilibrium within breed (*P* ≤ 0.01) were removed. The dataset, at this stage, consisted of 46,733 autosomal SNPs genotyped in 3095 individuals.

### Haplotyping and genome-wide relationships (GRM)

The Beagle software package (v5.0) was used to impute missing genotypes [44] and perform haplotype phasing [45]. To improve the efficiency of these processes, genotyping data from all animals available in the in-house database were incorporated. This database contains a considerable number of pairs and trios from various projects, e.g. [46]. Genome-wide relationships between individuals were estimated using a genome-wide relationship matrix (GRM) as described by Yang et al. [47] (utilizing the average observed allele frequencies of the entire multi-breed dataset). This was implemented in the R package *snpReady* [48], based on 46,733 single-nucleotide polymorphism (SNP) genotypes from 3095 animals. To analyze diversity, phylogeny, and population structure, it is essential to select animal samples that are both representative of each breed and minimally related. To ensure representativeness we excluded outlier individuals, which were identified as admixed, by using a multivariate outlier analysis [49]; this approach was described in Ramljak et al. [50] and Pogorevc et al. [51]. The overall relatedness within the dataset was further reduced by progressively removing highly related individuals, based on each breed’s population size. A more lenient approach was adopted concerning breeds comprising a small total and sampled number of animals, i.e. related animals were not removed. These analyses were conducted based on genome-wide additive genetic relationships derived from the GRM matrix. Ultimately, the final dataset used for diversity and phylogenetic analyses consisted of 2623 of the most representative and least related animals. Following this strict quality control and the removal of highly related individuals, four Greek populations (Rodos, Ikaria, Tsipoureika, and Roumlouki) retained sample sizes that were too small to reliably estimate genetic diversity and admixture within these populations. Consequently, these four populations were excluded from all subsequent analyses, reducing the core dataset to 123 populations, with the sole exception of the *TreeMix* analysis (Fig. 1). The initial and optimized sample sizes for each breed are detailed in columns *N*, *N*_*d*_ and *N*_*al*_ (Additional file 1 Table S1).

### Haplotype diversity

Genotyping with the OvineSNP50 BeadChip kit creates a bias toward artificially selected or improved sheep breeds at the expense of many local breeds. The Illumina OvineSNP50 BeadChip was designed based on SNPs discovered and validated across more than 75 sheep breeds worldwide. However, according to the Illumina OvineSNP50 BeadChip datasheet [52], only the Chios and Cyprus fat-tailed breeds were included in the original validation panel. As is apparent, despite the broad sampling of the Beadchip, the Greek breeds are underrepresented. This limited representation of Greek genetic diversity could introduce ascertainment bias when analyzing native Greek breeds. Due to the inherent bias that is created by this kit when using pre-ascertained single-nucleotide polymorphisms (SNPs) [53], a 4-SNP-block approach was adopted, as previously described [54], to reduce this ascertainment bias. Specifically, 4-SNP blocks (haplotypes) that spanned less than 150 kilobases and had an inter-marker distance shorter than 50 kilobases were defined, leading to a compromise between the maximum number of SNPs and the minimum recombination probability within the block. This approach has been utilized in other studies conducted on goats [51] and cattle [27, 54]. In order to assess the effect of ascertainment bias on diversity estimates, observed heterozygosity (*H*_*o*_) was estimated for each breed, using both SNPs and 4-SNP haplotype blocks. In total, 4347 SNP blocks were considered as multi-allelic markers and their haplotypes as alleles in subsequent unbiased allelic diversity and heterozygosity analyses. Henceforth, SNP blocks are also referred to as multi-allelic markers. A scatter plot was generated to examine the relationship between the two measures across all breeds, and a linear regression line with confidence intervals was fitted to evaluate the correlation between SNP-derived and block-derived estimates.

### Genetic diversity

Several population genetic parameters were estimated based on the 4347 multi-allelic haplotypic blocks, including the total number of alleles (*n*_*A*_), the mean number of alleles per block (*m*_*A*_), allelic richness (*A*_*R*_), observed heterozygosity (*H*_*o*_), and expected heterozygosity (*H*_*e*_). For comparison, *H*_*o*_ and *H*_*e*_ were also estimated using the individual SNPs. Additionally, private alleles (*np*_*A*_), defined as alleles present in only one population, and their frequency (*PAf*) were recorded, along with common alleles (*nc*_*A*_), which were shared across all subpopulations [54–56].

To further assess allelic diversity and explore genetic and geographical proximity between breed pairs, the number of semi-private alleles (*npA2*) present in only two populations was also considered. Relative inbreeding coefficients for each sheep (*Fi*) were estimated using the diagonal elements of the GRM matrix, following the formula *Fi* = *GRM (i,i)* − 1. *Fi* was estimated by SNPs.

To estimate the variance of heterozygosity at individual level as a measurement of genetic heterogeny or homogeny within each breed, multilocus heterozygosity (MLH) was estimated using the *inbreedR* package [57]. MLH was estimated by SNPs. For better visualization of the MLH values, a boxplot was generated using the *ggplot2* package in *R* [58].

To evaluate subpopulation differentiation, both *D*_*EST*_ and *G*_*ST*_ estimators were estimated. However, only *D*_*EST*_ was ultimately used, as it performs a similar function to *G*_*ST*_ for multi-allelic loci but is considered unbiased and more suitable in cases of high gene diversity [59]. This analysis was performed across 118 breeds, excluding the outgroups.

Also, weighted means were estimated for each geographic group for all genetic diversity parameters using breed sample size (***N***_***al***_) as weights, except for *Fi*, MLH, *H*_*o*_, and *H*_*e*_. *Fi* and MLH were estimated at the individual level, while *H*_*o*_ and *H*_*e*_ were estimated at the locus/population level and therefore not weighted.

### Past effective population size based on linkage disequilibrium (***Ne***)

To estimate the historical and recent effective population size (*Ne*) a linkage disequilibrium (LD) based method was applied as implemented in the *SΝeP* tool [60]. *Ne* was estimated individually for each sheep breed. Default parameters were applied, apart from the sample size correction, occurrence of mutation (α = 2.2) [61], recombination rate between a pair of genetic markers according to Sved & Feldman [62] and finally minimum and maximum distances were set to 200,000 and 9,500,000 bp, respectively. The most recent *Ne*, was represented by five generations ago (*Ne*_*5*_), while the *Ne* in pre-industrial times is represented by 85 generations ago (*Ne*_*85*_). To facilitate the visualization and interpretation of *Ne* variations across time and geographic space, *Ne*_5_ and *Ne*_85_ (standardized) values were plotted by *ggplot2* in *R* [57]. The means of *Ne*_5_ and *Ne*_85_, for each group, were estimated. To further assess historical demographic fluctuations and potential non-linear population dynamics, we also estimated the historical *Ne* using the GONE software [63]. The analysis was conducted using default parameters, assuming a constant recombination rate of 1.5 cM/Mb [64], to evaluate *Ne* trajectories over the past 100 generations.

### Phylogeny and population structure

To explore the relationships among animals and breeds, four different phylogenetic and population structure analyses were performed. Two of these analyses were based on bi-allelic SNP genotypes (*Admixture, TreeMix*), while the other two utilized multi-allelic SNP-block genotypes (*Neighbor-net* based on Nei’s *D*_*A*_-distances, and *Multidimensional Scaling* based on *D*_*PS*_ distances). Moreover, two approaches followed a supervised clustering method (*TreeMix* and *Neighbor-net*), whereas the other two were unsupervised (*Admixture* and *Multidimensional Scaling*).

### Supervised phylogeny analysis

To clarify the phylogenetic relationships among the studied populations, maximum likelihood (ML) methods were applied using the *TreeMix* program [65]. This specific analysis utilized the complete dataset of 127 populations, uniquely retaining four Greek populations (Rodos, Ikaria, Tsipoureika, and Roumlouki) that were excluded from subsequent diversity and structure analyses due to their small sample sizes after quality control. This analysis utilized allele counts of single-nucleotide polymorphisms (SNPs) genotyped in 127 breeds, for each breed and marker separately, with *Ovis nivicola* (ONLRU) designated as the outgroup for rooting the tree. The *TreeMix* analyses were performed using the *scalepopgen* package [66]. The second supervised approach uses Nei’s *D*_*A*_-distances [67] based on allele frequencies from 4347 haplotype blocks. The resulting *D*_*A*_ distance matrix of 123 breeds was then used to construct a neighbor-net with *SplitsTree* App V.6.0 [68].

### Unsupervised population structure analyses

Similarly to the above analysis, the population structure was initially examined based on SNPs genotypes. To this end, clustering was conducted using the *Admixture* 1.23 program [69], operating under the assumption that the number of clusters is equal to *K*, with *K* ranging from 1 to 118, representing all 118 breeds. Since the admixture analysis does not require an outgroup, we excluded the Snow sheep (*Ovis nivicola*) and the four mouflon subpopulations. We also removed additional closely related individuals from the Greek breeds. The new sample sizes used in the admixture analysis are provided in column *N*_*ad*_ of Additional file 1 Table S1. To assess the quality of the clustering and thus infer the most likely *K*, 10 cross-validations were performed [70] and the cross-validation error for each *K* was estimated. The *Admixture* analyses were performed using the *scalepopgen* package [66] and to illustrate the results, the *R* package *Pophelper* [71] was used.

The second unsupervised approach evaluated genome-wide allele-sharing distances based on multiallelic SNP blocks from 2623 animals. The allele-sharing distance matrix (*D*_*PS*_) was estimated following the methodology outlined by Bowcock et al. [72]. Multidimensional scaling (MDS) [73] was then applied to project the high-dimensional *D*_*PS*_ matrix onto a two-dimensional (2D) plane. To improve the clarity and interpretability of the MDS results and the visualization of genetic relationships among the 123 studied breeds, the mean MDS coordinates of all individuals within each breed were computed, representing the central position of the breed symbol (circle). The standard deviation (SD) around this centroid was then estimated to quantify the dispersion of individuals within each breed. Specifically, for each breed, the spatial distance of every individual from the breed centroid was determined using the Pythagorean theorem, where the hypotenuse represented the distance between an individual’s position and the breed’s center. The SD of these spatial distances was subsequently estimated and utilized as the radius of the breed symbol, serving as a pr for the spatial dispersion of individuals within the breed. For visualization purposes, the plot dimensions were proportionally adjusted in *R*, with the longest possible radius set to 1 inch (2.54 cm).

## Results

### Genetic diversity

Heterozygosity estimates, derived from SNPs and haplotype blocks, are shown in Additional file 6 Fig. S1. Some breeds with elevated *Ho* (blocks), such as Turkish and Egyptian, appear below the trendline, suggesting an underestimation of their values due to the lower SNP-derived values for these breeds. Conversely, breeds with low *Ho* (blocks), including those from Central Europe and Italy, appear above the trendline, indicating an overestimation of their values due to their higher SNP-derived values. Due to ascertainment bias in the SNP array, heterozygosity values are overestimated in some western breeds while underestimated in some eastern breeds. However, estimating diversity parameters using both SNPs and haplotype blocks provides a reliable representation of levels of genetic variation.

A total of 96 common alleles (blocks) were identified across the 123 ovine populations analyzed, accounting for just 0.14% of the overall 65,757 alleles. These 96 ancestral 4-SNP haplotypes are present in Snow sheep, all mouflons, and all sheep breeds. Furthermore, the average number of alleles per haplotype block in Snow sheep is 1.11, meaning that there is usually only a single haplotype with ancestral alleles. As it is shown in Table 1, most genetic diversity estimators for the breeds in this study exhibited significant differentiation among the predefined geographical groups. Compared to the other geographical groups, the Greek group exhibits intermediate diversity parameter values, falling between the higher diversity observed in the Southwest Asian/North African groups and the lower diversity of the Northwestern European group. Specifically, the Greek group has the following intermediate diversity parameters values: total number of alleles ($\overline{{n_{A} }}$ = 33,973), number of alleles per haplotype block ($\overline{{m_{A} }}$ = 7.81), observed (*H*_*o*_ = 0.750) and expected heterozygosity (*H*_*e*_ = 0.744) and allelic richness (*A*_*R*_ = 4.86). Intermediate values were found for the number of private ($\overline{{np_{A} }}$ = 14.59) and semi-private alleles ($\overline{{np_{A2} }}$ = 22.83), while the inbreeding coefficient was similar to the other groups ($\overline{F}$ = 0.09), except one group that had the highest value (Central Europe to Northwest Europe; $\overline{F}$ = 0.18). Within the Greek sheep breeds, Lesvos breed (LESGR) has the highest values for most diversity parameters (total number of alleles (*n*_*A*_ = 44,309), number of alleles per haplotype block (*m*_*A*_ = 10.10) and expected heterozygosity (*H*_*e*_ = 0.792) based on multi-allelic SNP blocks. On the opposite, the above parameters have the lowest values in Agrinio breed (AGRGR) (*n*_*A*_ = 23,066, *m*_*A*_ = 5.31, *H*_*e*_ = 0.675). The highest observed heterozygosity (*H*_*o*_ = 0.795) was displayed by the Karagouniko breed (KRGGR), while the lowest (*H*_*o*_ = 0.672) by the Thraki breed (THRGR). Thraki breed, also, exhibits the lowest allelic richness (*A*_*R*_ = 4.08) and the highest inbreeding coefficient (F = 0.19). On the other hand, the highest allelic richness (*A*_*R*_ = 5.21) and lowest inbreeding coefficient ($\overline{F}$ = 0.04) is displayed by Vlahiko breed (VLHGR). Lastly, the Chios breed (HIOGR) has the lowest average frequency of private alleles ($\overline{PAf}$ = 0.014) and the highest number of private alleles (np_A_ = 43), while Arta breed (ARTGR) as a distinct case of synthetic breed has the highest ($\overline{PAf}$ = 0.227) and lowest (*np*_*A*_ = 1), respectively.

The geographical group of “Central Europe to Northwest” displays the lowest average values for all the estimators of allelic diversity (Additional file 3 Table S5) i.e., total number of alleles ($\overline{{n_{A} }}$ = 28,069), number of alleles per haplotype block ($\overline{{m_{A} }}$ = 6.46), number of private ($\overline{{np_{A} }}$ = 7.39) and semi-private alleles ($\overline{{np_{A2} }}$ = 14.93), observed (*H*_*o*_ = 0.698) and expected heterozygosity (*H*_*e*_ = 0.685), allelic richness (*A*_*R*_ = 4.17), while having the highest inbreeding coefficient ($\overline{F}$ = 0.18). The Italian group has the lowest inbreeding ($\overline{F}$ = 0.08). The “Southwest Asian and North African” group displays the highest values of the number of private ($\overline{{np_{A} }}$ = 30.77) and semi-private alleles ($\overline{{np_{A2} }}$ = 43.35) and the observed heterozygosity (*H*_*o*_ = 0.771). The group of “Central Europe to West” displays the highest expected heterozygosity (*H*_*e*_ = 0.757). The above group, also, exhibits the highest values for the total number of alleles ($\overline{{n_{A} }}$ = 36,123) and the number of alleles per haplotype block ($\overline{{m_{A} }}$ = 8.31). The Balkans group has the highest allelic richness value (*A*_*R*_ = 4.99).

**Table 1:** Parameters of genetic diversity in the 123 examined sheep breeds with 46,733 SNPs & 4347 blocks

Breed	Code	*N*_*al*_	*n*_*A*_	*m*_*A*_	*H*_*o*_	*H*_*e*_	*Hdef*	*np*_*A*_
Outgroups								
Snow sheep	ONLRU	24	4844	1.11	0.240	0.027	− 8.014	30
Asian Mouflon	OGGIR	19	30,820	7.09	0.659	0.720	0.085	223
Cyprus Mouflon	OGPCY	25	8650	1.99	0.389	0.266	− 0.461	8
Sardinian Mouflon	OGMIT	17	24,697	5.68	0.723	0.659	− 0.097	6
Corsican Mouflon	OGMFR	14	13,406	3.08	0.489	0.461	− 0.06	15
Southwest Asia & North Africa								
Buubei	BUURU	17	33,018	7.60	0.800	0.753	− 0.063	17
Tuvan	TUVRU	16	35,991	8.28	0.800	0.775	− 0.033	31
Karakul (Romania)	KARKZ	28	41,133	9.46	0.777	0.780	0.004	57
Zel	ZELIR	24	38,879	8.94	0.773	0.765	− 0.011	50
Lori − Bakhtiari	LBAIR	24	36,908	8.49	0.773	0.756	− 0.022	28
Moghani	MOGIR	33	41,194	9.48	0.791	0.778	− 0.017	47
Afshari	AFSIR	33	34,857	8.02	0.770	0.743	− 0.036	21
Qezel	QEZTR	35	43,913	10.10	0.782	0.786	0.004	61
Norduz	NDZTR	20	28,913	6.65	0.761	0.712	− 0.069	7
Karakas	KRSTR	17	29,330	6.75	0.767	0.732	− 0.047	10
Lezgin	LEZRU	15	34,816	8.01	0.811	0.771	− 0.052	27
Karachaev	KRHRU	16	35,967	8.27	0.801	0.776	− 0.032	39
Local Awassi	AWSIL	24	38,305	8.81	0.776	0.767	− 0.012	33
Cyprus Fat-tailed	CFTCY	36	27,215	6.26	0.698	0.675	− 0.033	5
Ossimi	OSSEG	8	26,929	6.19	0.754	0.733	− 0.029	24
Egyptian Barki	BRKEG	13	32,669	7.52	0.753	0.755	0.003	20
Sakiz	SKZTR	16	21,423	4.93	0.726	0.648	− 0.121	9
			$\overline{nA}$ = 35,229	$\overline{mA}$ = 8.10	$\overline{H}_{o}$ = 0.771	$\overline{H}_{e}$ = 0.747		$\overline{npA}$ = 30.77
Greece								
Karpathos	KRPGR	13	27,316	6.28	0.687	0.724	0.051	10
Kasos	KSOGR	30	35,670	8.21	0.767	0.762	− 0.006	13
Sitia	SHTGR	13	30,184	6.94	0.776	0.749	− 0.037	7
Lasithi Plateu (Local)	PLSGR	11	30,582	7.04	0.778	0.757	− 0.028	17
Asterousia	ASTGR	26	39,098	8.99	0.772	0.782	0.013	17
Anogia	ANGGR	13	33,044	7.60	0.792	0.769	− 0.031	23
Sfakia	SFKGR	31	38,211	8.79	0.755	0.769	0.018	6
Chios	HIOGR	46	34,166	7.86	0.702	0.714	0.018	43
Lesvos	LESGR	48	44,309	10.10	0.779	0.792	0.016	13
Skyros	SKYGR	12	30,826	7.09	0.772	0.757	− 0.02	21
Karystos	KRYGR	22	35,393	8.14	0.761	0.762	0.002	9
Kymi	KYMGR	15	30,198	6.95	0.736	0.738	0.003	18
Kalarrytiko	KLRGR	21	35,463	8.16	0.767	0.766	− 0.001	11
Kokovitiko	KOKGR	11	29,847	6.87	0.687	0.753	0.087	11
Argos	ARGGR	20	29,820	6.86	0.737	0.727	− 0.013	3
Vlahiko Peloponnese	VLPGR	7	26,272	6.04	0.786	0.742	− 0.06	5
Zakynthos	ZAKGR	12	28,239	6.50	0.774	0.732	− 0.057	28
Kefalonia	KEFGR	28	39,581	9.11	0.769	0.781	0.016	6
Arta	ARTGR	14	32,142	7.39	0.768	0.760	− 0.01	1
Agrinio	AGRGR	11	23,066	5.31	0.708	0.675	− 0.049	10
Skopelos	SKPGR	17	31,296	7.20	0.730	0.738	0.011	14
Pelion	PELGR	22	36,097	8.30	0.749	0.773	0.031	27
Vlahiko	VLHGR	25	39,448	9.07	0.794	0.785	− 0.011	16
Karagouniko	KRGGR	23	37,841	8.71	0.795	0.780	− 0.02	10
Thraki	THRGR	14	24,349	5.60	0.672	0.679	0.01	3
Serres	SERGR	11	26,263	6.04	0.709	0.720	0.015	7
Pelagonia	PLGGR	8	24,288	5.59	0.745	0.704	− 0.058	21
Sarakatsaniko	SRKGR	20	34,817	8.01	0.740	0.765	0.033	7
Katsika	KTSGR	10	26,180	6.02	0.753	0.725	− 0.039	5
Epirus Orino	BUTGR	10	27,343	6.29	0.784	0.730	− 0.073	4
Katafygio	KTFGR	7	23,180	5.33	0.743	0.700	− 0.062	2
Lefkimmi	LEUGR	10	24,901	5.73	0.726	0.699	− 0.038	36
			$\overline{nA}$ = 33,973	$\overline{mA}$ = 7.81	$\overline{H}_{o}$ = 0.750	$\overline{H}_{e}$ = 0.744		$\overline{npA}$ = 14.59
Italy (South and Middle)								
Comisana	COMIT	24	38,044	8.75	0.793	0.775	− 0.023	36
Pinzirita	PINIT	24	39,659	9.12	0.768	0.787	0.023	9
Valle del Belice	VDBIT	24	32,347	7.44	0.725	0.739	0.019	11
Sardinian White	SAWIT	24	32,908	7.57	0.762	0.744	− 0.025	10
Sardinian Ancestral Black	SABIT	20	29,628	6.82	0.761	0.707	− 0.076	23
Leccese	LECIT	22	37,303	8.58	0.747	0.780	0.042	5
Altamurana	ALMIT	23	26,587	6.12	0.722	0.695	− 0.038	34
Bagnolese	BAGIT	24	40,226	9.25	0.780	0.791	0.014	34
Laticauda	LATIT	24	37,693	8.67	0.771	0.776	0.006	7
Gentile di Puglia	GENIT	24	31,980	7.36	0.769	0.742	− 0.036	13
Sopravissana	SOPIT	24	38,130	8.77	0.773	0.783	0.013	12
Appenninica	APPIT	24	35,069	8.07	0.781	0.759	− 0.028	19
Fabrianese	FABIT	24	33,181	7.63	0.787	0.748	− 0.051	15
Massese	MASIT	24	32,763	7.54	0.758	0.747	− 0.014	7
			$\overline{nA}$ = 34,750	$\overline{mA}$ = 7.99	$\overline{H}_{o}$ = 0.764	$\overline{H}_{e}$ = 0.755		$\overline{npA}$ = 16.73
Balkans								
Karakachanska	KRCMK	6	14,878	3.42	0.646	0.558	− 0.157	7
Ovchepolean	OVPMK	10	31,715	7.30	0.792	0.773	− 0.025	13
Balusha	BLSXK	25	34,255	7.88	0.755	0.750	− 0.008	12
Ruda	RUDAL	16	36,147	8.32	0.761	0.778	0.021	18
Pramenka (Sijenica)	SJEBA	16	37,097	8.53	0.793	0.787	− 0.008	20
Pramenka (Privor)	PRIBA	15	34,479	7.93	0.784	0.771	− 0.018	26
			$\overline{nA}$ = 33,544	$\overline{mA}$ = 7.72	$\overline{H}_{o}$ = 0.755	$\overline{H}_{e}$ = 0.736		$\overline{npA}$ = 16.70
Alpe-Adria								
Rab	RABHR	25	37,715	8.68	0.781	0.776	− 0.006	18
Istrian	ISTSI	24	33,483	7.70	0.776	0.756	− 0.026	29
Alpagota	ALPIT	24	33,574	7.72	0.752	0.749	− 0.005	23
Bergamasca	BERIT	24	37,306	8.58	0.773	0.772	− 0.001	22
Sambucana	SAMIT	24	36,536	8.40	0.771	0.767	− 0.006	50
Biellese	BIEIT	24	38,983	8.97	0.776	0.778	0.003	10
Delle Langhe	DLLIT	24	30,337	6.98	0.730	0.726	− 0.006	2
Bundner Oberländer	BOSCH	21	28,299	6.51	0.759	0.720	− 0.053	2
Mirror	SMSCH	20	29,503	6.79	0.753	0.726	− 0.038	7
Black-Brown Mountain	SBSCH	23	30,055	6.91	0.747	0.729	− 0.024	3
White Alpine	SWACH	21	30,441	7.00	0.747	0.726	− 0.03	9
Valais Blacknose	VBSCH	21	24,811	5.71	0.646	0.671	0.037	3
Valais Red	VRSCH	17	22,870	5.26	0.699	0.662	− 0.055	16
Engadine Red	ERSCH	21	34,400	7.91	0.783	0.764	− 0.025	15
			$\overline{nA}$ = 32,381	$\overline{mA}$ = 7.45	$\overline{H}_{o}$ = 0.750	$\overline{H}_{e}$ = 0.737		$\overline{npA}$ = 15.35
From Panonia to the North								
Racka	RAKHU	15	29,759	6.85	0.732	0.729	− 0.003	21
Tsigaia	TSIRO	26	42,379	9.75	0.796	0.799	0.004	9
Cikta	CIKHU	8	24,576	5.65	0.772	0.715	− 0.081	3
Kamieniec	KAMPL	6	23,681	5.45	0.770	0.721	− 0.067	10
Kuchugur	KUHRU	15	28,863	6.64	0.773	0.704	− 0.098	21
Romanov	ROMRU	32	32,218	7.41	0.733	0.730	− 0.004	27
Finnsheep	FINFI	45	35,861	8.25	0.748	0.754	0.008	9
			$\overline{nA}$ = 33,773	$\overline{mA}$ = 7.77	$\overline{H}_{o}$ = 0.760	$\overline{H}_{e}$ = 0.736		$\overline{npA}$ = 15.08
From Central Europe to Northwest								
Merinolandschaf	MLSDE	44	36,787	8.46	0.765	0.757	− 0.011	3
Black-Headed Mutton	BHMDE	17	28,886	6.65	0.729	0.721	− 0.011	11
German Heath	GHHDE	7	22,701	5.22	0.687	0.693	0.008	10
Drenthe Horned Heath	DHTNL	5	19,536	4.49	0.738	0.662	− 0.114	4
East-Friesian	EFRDE	23	27,080	6.23	0.695	0.688	− 0.01	9
Texel	TXLNL	24	26,968	6.20	0.702	0.693	− 0.014	7
Suffolk	SUFUK	19	26,295	6.05	0.714	0.693	− 0.03	16
Scottish Blackface	SBFUK	34	34,858	8.02	0.767	0.751	− 0.022	6
Soay	SOYUK	45	19,483	4.48	0.583	0.568	− 0.025	4
Boreray	BORUK	14	17,800	4.09	0.569	0.552	− 0.032	11
Old Norwegian Spæl	NSONO	15	30,023	6.91	0.722	0.737	0.02	14
Spæl-White	NSWNO	29	29,297	6.74	0.705	0.706	0	7
			$\overline{nA}$ = 28,069	$\overline{mA}$ = 6.46	$\overline{H}_{o}$ = 0.698	$\overline{H}_{e}$ = 0.685		$\overline{npA}$ = 7.39
From Central Europe to West								
Ile de France	IDFFR	23	27,435	6.31	0.713	0.700	− 0.018	11
Milk-Lacaune	LALFR	39	35,603	8.19	0.767	0.755	− 0.016	7
Meat-Lacaune	LAMFR	30	36,644	8.43	0.772	0.765	− 0.009	17
Berrichon du Cher	BDCFR	19	21,913	5.04	0.655	0.636	− 0.031	10
Merinos de Rambouillet	MDRFR	45	37,516	8.63	0.736	0.748	0.016	14
Merinos d'Arles	MDAFR	18	34,660	7.97	0.785	0.765	− 0.025	56
Latxa	LTXES	19	34,576	7.95	0.747	0.755	0.011	33
Sasi-Ardi	SAAES	24	38,437	8.84	0.770	0.772	0.003	31
Ripollesa	RIPES	21	37,382	8.60	0.776	0.773	− 0.003	40
Xisqueta	XISES	24	40,510	9.32	0.800	0.786	− 0.018	37
Rasa Aragonesa	RAAES	20	40,041	9.21	0.814	0.797	− 0.021	23
Churra	CHUES	45	40,458	9.31	0.773	0.771	− 0.003	41
Castellana	CASES	22	37,914	8.72	0.799	0.782	− 0.022	33
Ojalada	OJAES	24	38,546	8.87	0.800	0.782	− 0.022	25
Segurena	SEGES	12	32,738	7.53	0.796	0.766	− 0.038	11
Merino Estremadura	MEDES	13	32,261	7.42	0.784	0.764	− 0.027	0
			$\overline{nA}$ = 36,123	$\overline{mA}$ = 8.31	$\overline{H}_{o}$ = 0.768	$\overline{H}_{e}$ = 0.757		$\overline{npA}$ = 24.44

Breed	*np*_*A2*_	$\overline{PAf}$	*F*	*A*_*R*_	*H*_*ο*_ (SNPs)	*H*_*e*_ (SNPs)	*Ne*_*5*_	*Ne*_*85*_
Outgroups								
Snow sheep	43	0.833	1.11	1.08	0.007	0.007	–	–
Asian Mouflon	234	0.071	0.35	4.54	0.268	0.296	77	888
Cyprus Mouflon	20	0.253	0.79	1.77	0.098	0.101	138	169
Sardinian Mouflon	26	0.088	0.17	3.94	0.337	0.305	54	482
Corsican Mouflon	23	0.124	0.52	2.60	0.203	0.202	64	130
Southwest Asia & North Africa								
Buubei	32	0.052	0.06	4.88	0.374	0.349	108	976
Tuvan	38	0.035	0.06	5.22	0.373	0.358	247	2506
Karakul (Romania)	69	0.021	0.08	5.16	0.362	0.36	713	3287
Zel	81	0.023	0.10	5.06	0.347	0.348	824	6299
Lori-Bakhtiari	58	0.028	0.10	4.93	0.348	0.344	329	1821
Moghani	65	0.023	0.07	5.09	0.367	0.358	332	2411
Afshari	36	0.033	0.09	4.65	0.358	0.344	141	881
Qezel	76	0.016	0.08	5.21	0.364	0.362	1037	6707
Norduz	15	0.054	0.10	4.36	0.355	0.33	71	586
Karakas	15	0.047	0.10	4.60	0.356	0.339	79	699
Lezgin	42	0.041	0.04	5.18	0.38	0.358	162	1883
Karachaev	48	0.035	0.05	5.23	0.375	0.361	298	2448
Local Awassi	42	0.025	0.10	5.06	0.35	0.351	312	2605
Cyprus Fat-tailed	6	0.064	0.17	3.93	0.325	0.313	73	325
Ossimi	22	0.065	0.13	5.03	0.337	0.333	87	1125
Egyptian Barki	34	0.042	0.12	5.09	0.34	0.346	192	1963
Sakiz	3	0.043	0.12	3.74	0.341	0.303	45	261
	$\overline{npA2}$ = 43.35		$\overline{F}$ = 0.09	$\overline{AR}$ = 4.83			$\overline{{Ne_{5} }}$ = 297	
Greece								
Karpathos	5	0.028	0.17	4.57	0.322	0.338	97	619
Kasos	21	0.044	0.08	4.84	0.36	0.355	562	1516
Sitia	10	0.046	0.06	4.88	0.366	0.349	109	1026
Lasithi Plateu (Local)	23	0.025	0.07	5.10	0.364	0.353	148	1492
Asterousia	36	0.039	0.07	5.14	0.362	0.365	416	2636
Anogia	22	0.020	0.05	5.18	0.372	0.358	269	2552
Sfakia	38	0.015	0.09	4.96	0.354	0.357	501	2146
Chios	5	0.014	0.15	4.33	0.33	0.334	516	624
Lesvos	53	0.051	0.06	5.18	0.366	0.37	915	3412
Skyros	12	0.031	0.07	5.04	0.363	0.353	117	1076
Karystos	19	0.067	0.08	4.93	0.358	0.355	254	1138
Kymi	9	0.029	0.11	4.72	0.347	0.346	121	744
Kalarrytiko	27	0.054	0.07	4.98	0.36	0.357	183	1201
Kokovitiko	20	0.073	0.16	5.03	0.324	0.353	110	1049
Argos	22	0.071	0.11	4.51	0.346	0.341	86	566
Vlahiko Peloponnese	8	0.067	0.05	5.17	0.372	0.347	76	941
Zakynthos	15	0.019	0.06	4.73	0.366	0.343	92	697
Kefalonia	42	0.042	0.07	5.13	0.361	0.365	346	1919
Arta	12	0.227	0.07	5.02	0.365	0.36	160	1123
Agrinio	10	0.035	0.14	4.14	0.335	0.316	54	344
Skopelos	12	0.031	0.12	4.71	0.344	0.347	179	783
Pelion	20	0.024	0.09	5.05	0.353	0.363	220	1405
Vlahiko	34	0.030	0.04	5.21	0.374	0.367	346	2172
Karagouniko	29	0.104	0.04	5.14	0.375	0.367	245	1732
Thraki	9	0.061	0.19	4.08	0.317	0.319	40	303
Serres	7	0.080	0.14	4.58	0.333	0.336	60	522
Pelagonia	6	0.037	0.10	4.62	0.352	0.33	71	533
Sarakatsaniko	28	0.064	0.10	4.98	0.349	0.358	166	1143
Katsika	13	0.060	0.09	4.68	0.354	0.338	82	601
Epirus Orino	15	0.071	0.05	4.80	0.37	0.342	69	683
Katafygio	12	0.050	0.10	4.64	0.35	0.324	57	524
Lefkimmi	6	0.026	0.12	4.43	0.343	0.328	60	510
	$\overline{npA2}$ = 22.83		$\overline{F}$ = 0.09	$\overline{AR}$ = 4.86			$\overline{{Ne_{5} }}$ = 210	
Italy (South and Middle)								
Comisana	42	0.024	0.05	5.09	0.375	0.362	365	1585
Pinzirita	60	0.035	0.08	5.23	0.362	0.368	250	2137
Valle del Belice	18	0.027	0.13	4.61	0.342	0.346	109	633
Sardinian White	24	0.035	0.08	4.67	0.36	0.349	179	902
Sardinian Ancestral Black	14	0.036	0.09	4.37	0.358	0.332	80	627
Leccese	26	0.039	0.10	5.14	0.354	0.366	178	1495
Altamurana	16	0.026	0.13	4.12	0.341	0.327	57	325
Bagnolese	51	0.027	0.05	5.29	0.372	0.372	327	2383
Laticauda	37	0.045	0.07	5.08	0.365	0.365	232	1384
Gentile di Puglia	10	0.021	0.07	4.64	0.366	0.351	87	646
Sopravissana	33	0.033	0.06	5.15	0.369	0.371	231	1740
Appenninica	19	0.032	0.05	4.85	0.372	0.359	159	928
Fabrianese	13	0.033	0.05	4.71	0.374	0.352	94	734
Massese	25	0.143	0.09	4.70	0.358	0.349	133	726
	$\overline{npA2}$ = 27.93		$\overline{F}$ = 0.08	$\overline{AR}$ = 4.84			$\overline{{Ne_{5} }}$ = 177	
Balkans								
Karakachanska	3	0.050	0.24	3.26	0.295	0.259	26	207
Ovchepolean	16	0.028	0.04	5.37	0.375	0.364	136	1657
Balusha	24	0.031	0.09	4.75	0.355	0.35	156	899
Ruda	27	0.035	0.09	5.26	0.35	0.363	222	2473
Pramenka (Sijenica)	35	0.035	0.04	5.37	0.373	0.369	301	3201
Pramenka (Privor)	27	0.029	0.05	5.17	0.369	0.36	215	1858
	$\overline{npA2}$ = 24.72		$\overline{F}$ = 0.09	$\overline{AR}$ = 4.99			$\overline{{Ne_{5} }}$ = 176	
Alpe-Adria								
Rab	39	0.031	0.06	5.07	0.367	0.363	244	1806
Istrian	29	0.043	0.07	4.80	0.366	0.355	138	938
Alpagota	39	0.022	0.11	4.73	0.352	0.35	173	822
Bergamasca	38	0.035	0.08	5.05	0.364	0.362	599	1947
Sambucana	24	0.023	0.07	4.96	0.365	0.362	236	1157
Biellese	57	0.056	0.06	5.14	0.373	0.367	628	2264
Delle Langhe	22	0.107	0.13	4.45	0.344	0.342	137	558
Bundner Oberländer	6	0.025	0.08	4.38	0.363	0.342	84	453
Mirror	6	0.040	0.10	4.47	0.36	0.346	103	511
Black-Brown Mountain	12	0.024	0.10	4.48	0.358	0.347	172	571
White Alpine	6	0.040	0.11	4.50	0.359	0.348	347	664
Valais Blacknose	15	0.137	0.22	3.95	0.304	0.314	197	417
Valais Red	8	0.036	0.16	3.88	0.33	0.312	79	326
Engadine Red	21	0.054	0.06	4.93	0.372	0.36	228	1198
	$\overline{npA2}$ = 24.06		$\overline{F}$ = 0.10	$\overline{AR}$ = 4.66			$\overline{{Ne_{5} }}$ = 240	
From Panonia to the North								
Racka	15	0.021	0.11	4.62	0.344	0.338	101	630
Tsigaia	54	0.090	0.03	5.39	0.378	0.376	822	4920
Cikta	12	0.111	0.08	4.71	0.357	0.334	54	519
Kamieniec	5	0.080	0.11	5.02	0.359	0.343	53	590
Kuchugur	17	0.053	0.07	4.40	0.368	0.332	70	531
Romanov	30	0.039	0.15	4.48	0.343	0.339	322	976
Finnsheep	36	0.040	0.13	4.70	0.352	0.353	445	1149
	$\overline{npA2}$ = 31.22		$\overline{F}$ = 0.10	$\overline{AR}$ = 4.75			$\overline{{Ne_{5} }}$ = 267	
From Central Europe to Northwest								
Merinolandschaf	11	0.039	0.08	4.73	0.364	0.358	355	858
Black-Headed Mutton	9	0.078	0.14	4.49	0.35	0.344	116	608
German Heath	9	0.130	0.19	4.55	0.321	0.321	54	475
Drenthe Horned Heath	7	0.071	0.12	4.49	0.345	0.307	49	429
East-Friesian	5	0.067	0.19	4.12	0.329	0.325	224	527
Texel	12	0.056	0.19	4.12	0.341	0.334	345	455
Suffolk	11	0.025	0.17	4.16	0.339	0.328	320	511
Scottish Blackface	36	0.072	0.11	4.69	0.366	0.356	260	883
Soay	14	0.241	0.33	3.16	0.27	0.264	247	211
Boreray	5	0.039	0.35	3.14	0.266	0.259	46	153
Old Norwegian Spæl	20	0.058	0.16	4.72	0.342	0.346	210	926
Spæl-White	19	0.040	0.18	4.24	0.332	0.33	291	543
	$\overline{npA2}$ = 14.93		$\overline{F}$ = 0.18	$\overline{AR}$ = 4.17			$\overline{{Ne_{5} }}$ = 210	
From Central Europe to West								
Ile de France	6	0.033	0.15	4.20	0.343	0.335	397	483
Milk-Lacaune	19	0.017	0.08	4.71	0.363	0.356	312	850
Meat-Lacaune	18	0.129	0.07	4.89	0.365	0.361	255	1119
Berrichon du Cher	15	0.014	0.24	3.66	0.31	0.299	154	219
Merinos de Rambouillet	15	0.032	0.11	4.67	0.351	0.355	1082	1043
Merinos d'Arles	26	0.037	0.05	5.02	0.372	0.36	372	1573
Latxa	56	0.027	0.12	4.93	0.341	0.35	199	1419
Sasi-Ardi	40	0.029	0.09	5.11	0.353	0.358	667	2744
Ripollesa	41	0.023	0.08	5.14	0.356	0.36	261	1897
Xisqueta	59	0.026	0.05	5.29	0.369	0.367	421	3808
Rasa Aragonesa	48	0.014	0.02	5.42	0.387	0.377	553	5426
Churra	46	0.028	0.07	4.91	0.367	0.364	340	1283
Castellana	48	0.027	0.04	5.18	0.381	0.369	165	1455
Ojalada	29	0.047	0.04	5.16	0.38	0.37	274	1754
Segurena	19	0.046	0.06	5.24	0.366	0.358	175	1844
Merino Estremadura	13	0.000	0.05	5.10	0.374	0.361	192	1553
	$\overline{npA2}$ = 30.97		$\overline{F}$ = 0.08	$\overline{AR}$ = 4.89			$\overline{{Ne_{5} }}$ = 364	

*N*_*al*_ = number of genotyped animals; *n*_*A*_ and $\overline{nA}$ = total and mean number of haplotypic alleles observed within subpopulation; *m*_*A*_ = n_A_/4347 (number of blocks); *H*_*o*_ = average observed heterozygosity within subpopulation; *H*_*e*_ = average expected heterozygosity within subpopulation; npA and $\overline{npA }$= number and mean number of private alleles; npA2 and $\overline{npA2}$ = number and mean number of alleles present only in two subpopulations; $\overline{PAf}$ = average frequency of private alleles, *F* and $\overline{F}$ = inbreeding coefficient per breed and per group; *A*_*R*_ and $\overline{AR}$ = number and mean value of allelic richness; *H*_*o*_ (SNPs) and *H*_*e*_ (SNPs) = observed and expected heterozygosity within subpopulation by SNPs; *Ne*_5_ and $\overline{{Ne_{5} }}$ = effective population number for 5 generations back per breed and per group (harmonic mean, *SNeP* values); *Ne*_85_ = effective population number for 85 generations back.

**Fig. 3:**
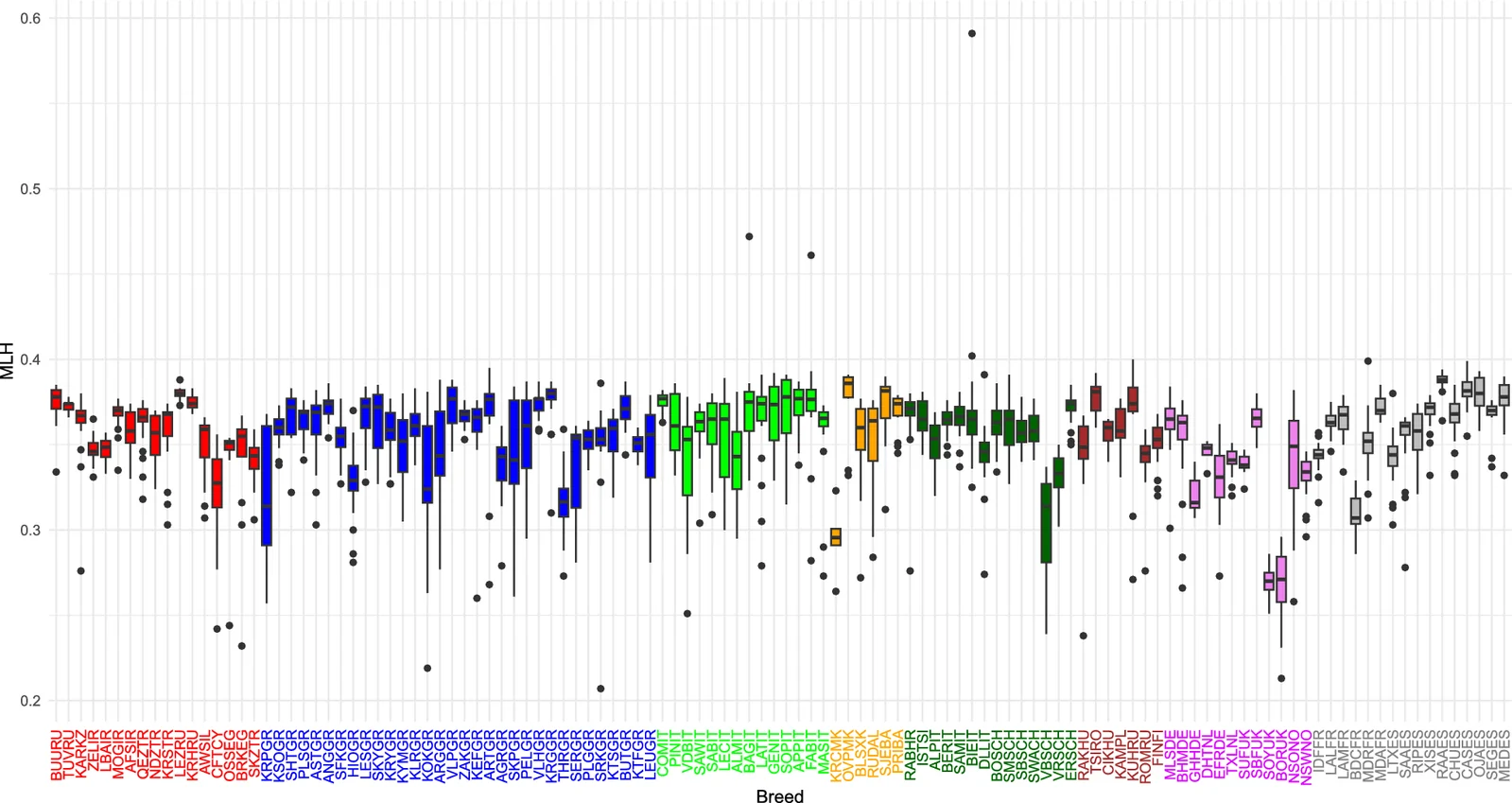
Box plots of the multilocus heterozygosity distribution within the 118 sheep breeds, using bi-allelic SNPs. From left to right: Buubei (BUURU); Tuvan (TUVRU); Karakul, (KARKZ); Zel (ZELIR); Lori-Bakhtiari (LBAIR); Moghani (MOGIR); Afshari (AFSIR); Qezel (QEZTR); Norduz (NDZTR); Karakas (KRSTR); Lezgin (LEZRU); Karachaev (KRHRU); Local Awassi (AWSIL); Cyprus Fat-Tail (CFTCY); Ossimi (OSSEG); Egyptian Barki (BRKEG); Sakiz (SKZTR); Rodos (KRPGR); Karpathos (KRPGR); Kasos (KSOGR); Sitia (SHTGR); Lasithi Plateau (PLSGR); Asterousia (ASTGR); Anogia (ANGGR); Sfakia (SFKGR); Chios (HIOGR); Lesvos (LESGR); Skyros (SKYGR); Karystos (KRYGR); Kymi (KYMGR); Kalarrytiko (KLRGR); Kokovitiko (KOKGR); Argos (ARGGR); Vlahiko Peloponnese (VLPGR); Zakynthos (ZAKGR); Kefalonia (KEFGR); Arta (ARTGR); Agrinio (AGRGR); Skopelos (SKPGR); Pelion (PELGR); Vlahiko (VLHGR); Karagouniko (KRGGR); Thraki (THRGR); Serres (SERGR); Pelagonia (PLGGR); Sarakatsaniko (SRKGR); Katsika (KTSGR); Epirus Orino (BUTGR); Katafygio (KTFGR); Lefkimmi (LEUGR); Comisana (COMIT); Pinzirita (PINIT); Valle del Belice (VDBIT); Sardinian White (SAWIT); Sardinian Ancestral Black (SABIT); Leccese (LECIT); Altamurana (ALMIT); Bagnolese (BAGIT); Laticauda (LATIT); Gentile di Puglia (GENIT); Sopravissana (SOPIT); Appenninica (APPIT); Fabrianese (FABIT); Massese (MASIT); Karakachanska (KRCMK); Ovchepolean (OVPMK); Balusha (BLSXK); Ruda (RUDAL); Pramenka Sjenica (SJEBA); Pramenka Privor (PRIBA); Rab (RABHR); Istrian (ISTSI); Alpagota (ALPIT); Bergamasca (BERIT); Sambucana (SAMIT); Biellese (BIEIT); Delle Langhe (DLLIT); Bündner Oberländer (BOSCH); Mirror (SMSCH); Black-Brown Mountain (SBSCH); White Alpine (SWACH); Valais Blacknose (VBSCH); Valais Red (VRSCH); Engadine Red (ERSCH); Racka (RAKHU); Tsigaia (TSIRO); Cikta (CIKHU); Kamieniec (KAMPL); Kuchugur (KUHRU); Romanov (ROMRU); Finnsheep (FINFI); Merinolandschaf (MLSDE); Black-Headed Mutton (BHMDE); German Heath (GHHDE); Drenthe Horned Heath (DHTNL); East-Friesian (EFRDE); Texel (TXLNL); Suffolk (SUFUK); Scottish Blackface (SBFUK); Soay (SOYUK); Boreray (BORUK); Old Norwegian Spæl (NSONO); Spæl White (NSWNO); Île de France (IDFFR); Milk Lacaune (LALFR); Meat Lacaune (LAMFR); Berrichon du Cher (BDCFR); Merinos de Rambouillet (MDRFR); Merinos d’Arles (MDAFR); Latxa (LTXES); Sasi-Ardi (SAAES); Ripollesa (RIPES); Xisqueta (XISES); Rasa Aragonesa (RAAES); Churra (CHUES); Castellana (CASES); Ojalada (OJAES); Segureña (SEGES); Merino de Extremadura (MEDES)

The distribution of MLH values is summarized in a boxplot (Fig. 3), allowing visual comparison of within- and between-breed genetic diversity. The results show that breeds with established pedigrees, structured improvement programs, and commercial value (e.g., TXLNL-Texel, SUFUK-Suffolk, IDFFR-Île-de-France, COMIT-Comisana) tend to exhibit a narrower range of MLH values, indicating more uniform levels of genetic diversity. In contrast, local or indigenous breeds across the examined dataset, particularly those from less intensively managed populations (e.g., KRPGR-Karpathos, VRSCH-Valais red, BLSXK-Balusha, RUDAL-Ruda), display a broader range of MLH, reflecting greater within-breed variation likely due to unstructured breeding. A particularly wide range of MLH was observed in some geographical groups, such as the Greek group but also in “Alpe-Adria” (e.g. VBSCH-Valais blacknose breed) and “Central Europe to Northwest” (e.g. NSONO-Old Norwegian Spæl breed) groups]. Within the Greek group, this wide range encompasses populations with highly contrasting demographic backgrounds. Specifically, a broad range of MLH is observed in breeds currently under in situ conservation programs (such as the SKPGR-Skopelos, PELGR-Pelion, and SERGR-Serres), in historically recognized breeds where the *in-situ* conservation has ceased (such as the LEUGR-Lefkimmi and ARGGR-Argos), as well as in breeds/ populations that are not officially recognized (such as the KOKGR-Kokovitiko and KRPGR-Karpathos). Nonetheless, the broader MLH ranges observed in indigenous Greek breeds/populations suggest a reservoir of genetic diversity valuable for breed resilience and adaptive potential. The correlation between MLH and the inbreeding coefficient (*F*), was −0.95 across all breeds and −1.00 among the Greek breeds.

### Effective population size (*Ne*)

Effective population size (*Ne*) estimates for each breed are presented in Table 1. Regarding the breeds and populations with large size, the sample size often does not reflect the whole genetic diversity of the breed. However, because the majority of the described Greek breeds/populations are rare with small census size, the sampling was adequate to reflect their genetic diversity. The parameters *Ne*_5_ and *Ne*_85_ represent effective population size estimates five and 85 generations ago, respectively. Assuming that the generational interval for sheep is three years, this corresponds to periods of 15 and 255 years, respectively—that is, the recent past and the time before industrialization. Across all breeds and geographical groups, *Ne* values were consistently higher in the past than in recent generations. Recent effective population sizes (*Ne*_5_) ranged from very low values in small or isolated populations to moderate or high values in larger or more widely distributed breeds, with the lowest *Ne*_5_ observed in the Karakachanska (KRCMK) breed (*Ne*_5_ = 26) and the highest in the Merinos de Rambouillet (MDRFR) breed (*Ne*_5_ = 1082), whereas historical estimates (*Ne*_85_) were substantially higher, reflecting larger ancestral population sizes. At the group level, the mean *Ne*_5_ ranged from 176 in the Balkan group to 364 in the “Central Europe to the West” group.

Compared to the other geographical groups, the Greek sheep breeds displayed low to intermediate *Ne* values and the group showed a moderate average recent value (*Ne*_5_ = 210), lower than that observed in the Asian (*Ne*_5_ = 297) and Western European groups (*Ne*_5_ = 364), but higher than that of the Balkan and Italian (*Ne*_5_ = 177) groups. Within the Greek group, *Ne* estimates were highly heterogeneous. Lesvos (LESGR) exhibited the highest recent historical *Ne* value (*Ne*_5_ = 915), followed by Kasos (KSOGR; *Ne*_5_ = 562) and Chios (HIOGR; *Ne*_5_ = 516). Lesvos also ranked third among all examined breeds, following Merinos de Rambouillet (MDRFR; *Ne*_5_ = 1082) and Qezel (QEZTR; *Ne*_5_ = 1037). In contrast, several mainland and island breeds showed markedly low recent *Ne* values, including Thraki (THRGR; *Ne*_5_ = 40), Agrinio (AGRGR; *Ne*_5_ = 54), Katafygio (KTFGR; *Ne*_5_ = 57) and Lefkimmi (LEUGR; *Ne*_5_ = 60).

Among all examined domestic breeds, the Chios breed showed the smallest difference between recent and historical effective population size estimates (*Ne*_5_ = 516, *Ne*_85_ = 624), while maintaining a high absolute *Ne*. Although a similarly flat *Ne* trajectory was observed in a small number of highly isolated or feral populations (e.g. Soay sheep and Cyprus Mouflon), these populations differed markedly from Chios in terms of genetic diversity and inbreeding levels.

**Fig. 4:**
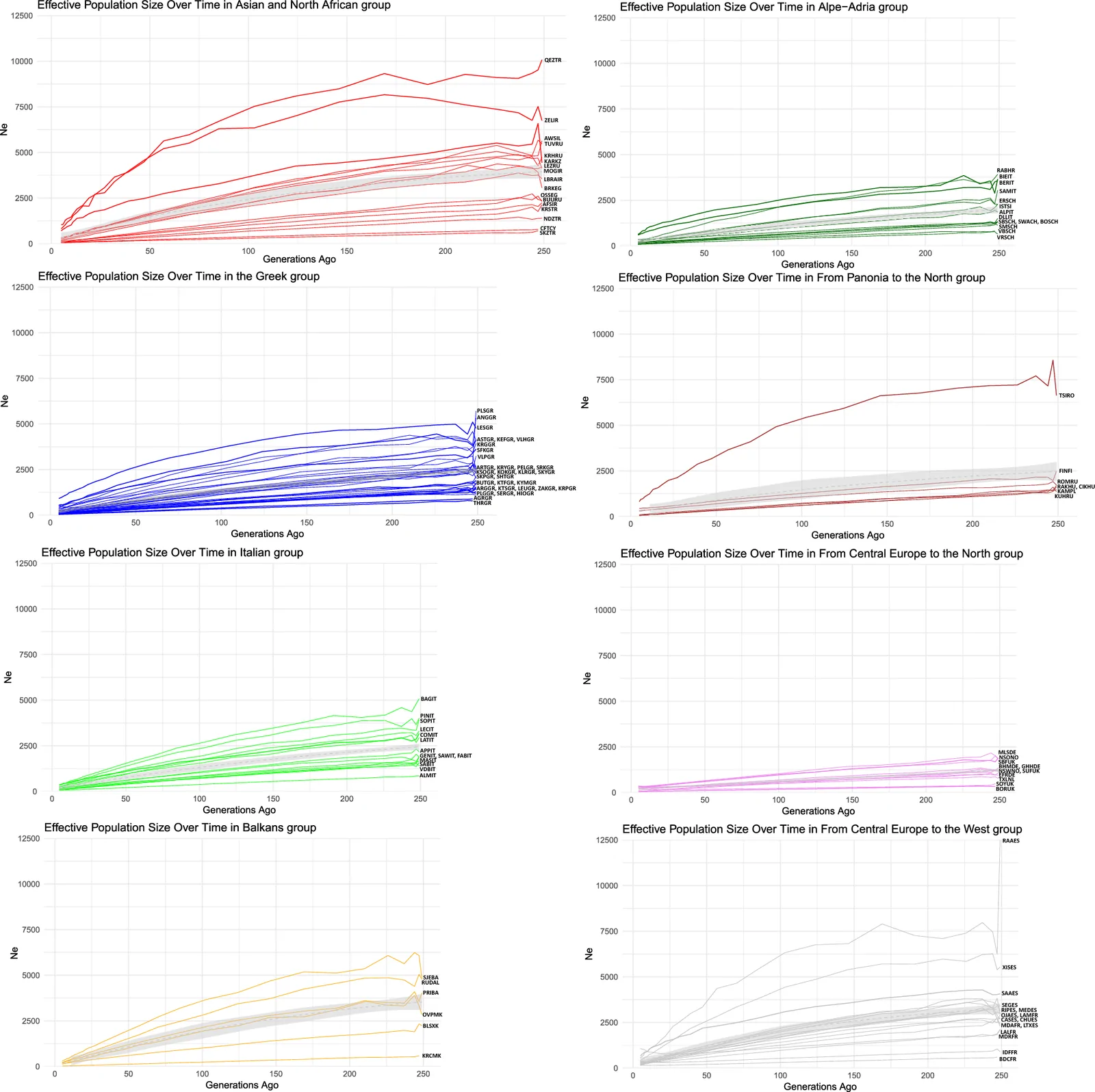
Plot of the Effective Population size over time for the 8 geographical groups of sheep breeds with trendlines highlighting demographic trajectories. The names of the breed groups are given according to their geographical distributions as shown in Fig. 2. For a full definition of the breed groups see [Additional file 1 Table S1]

Figure 4 illustrates the trajectory of effective population size (*Ne*) over time—up to 249 generations ago—for eight geographical groups of sheep breeds, based on a standardized sample size per breed. Across all groups, *Ne* decreased toward recent generations, indicating a general reduction in effective population size from the past to the present. However, both the magnitude of *Ne* and the shape of the trajectories differed among geographical groups. Breeds from Southwest Asia and the Balkans exhibited higher *Ne* values in the more distant past and more gradual declines over time, whereas Central and Northern European breeds showed consistently lower *Ne* values and steeper recent reductions. Greek breeds occupied an intermediate position, displaying heterogeneous *Ne* trajectories that ranged from relatively stable profiles to pronounced recent declines. Notably from Fig. 4, the Lasithi Plateau population (PLSGR) exhibited particularly high *Ne* values in the distant past (approximately 250 generations ago), followed by a pronounced reduction through time, resulting in substantially lower *Ne* estimates at 85 and 5 generations ago, relative to other Greek breeds. To further investigate potential historical demographic fluctuations—such as sudden population expansions or bottlenecks that linear-assumption methods might smooth over—we additionally analyzed the dataset using the GONE software. The resulting historical trajectories successfully captured dynamic demographic shifts and are provided in the supplementary material (Additional file 4 Table S6 and Additional file 7 Fig. S2). However, because GONE can be highly sensitive to small sample sizes and to the absence of a highly resolved genetic map, it produced biologically implausible overestimations of absolute *Ne* for several of the rare Greek breeds [63].

### Genetic differentiation

Pairwise population differentiation values were estimated using the unbiased *D*_*EST*_ (Jost’s estimate of differentiation) and *G*_*ST*_ (Nei’s coefficient of gene differentiation) for multi-allelic markers. These values are provided in Additional file 5 Table S7. The correlation between *D*_*EST*_ and *G*_*ST*_ was estimated to be 0.98. Among the 118 sheep breeds analyzed (excluding outgroups), *D*_*EST*_ values ranged from 0.0012 between the Pramenka (Sijenica) (SJEBA) and Ovchepolean (OVPMK) breeds to 0.4714 between the Karakachanska (KRCMK) and Soay (SOYUK) breeds.

Greek breeds showed relatively low differentiation, with an overall *D*_*EST*_ of 0.117. Within this group, the lowest pairwise *D*_*EST*_ value (0.0023) was observed between two Cretan breeds (Anogia (ANGGR)—Lasithi Plateau (PLSGR)), whereas the highest differentiation (*D*_*EST*_ = 0.2785) was found between two mainland breeds (Thraki (THRGR)—Agrinio (AGRGR)). The latter two breeds exhibit low genetic diversity and are geographically distant from each other, contributing to their high level of differentiation.

Focusing on the geographical groups, both the Greek sheep and the Cyprus fat-tailed breed belong to groups characterized by notably low genetic differentiation. Specifically, the Greek group exhibited a low level of differentiation (*D*_*EST*_ = 0.117), placing it among the least differentiated geographical groups. The Cyprus fat-tailed breed is part of the Asian and North African group, which displayed the lowest differentiation overall (*D*_*EST*_ = 0.103), closely followed by the neighboring Balkan group (*D*_*EST*_ = 0.105). The highest genetic differentiation (*D*_*EST*_ = 0.281) was observed within the “Central Europe to Northwest” group breeds, followed by the Alpe-Adria group (*D*_*EST*_ = 0.187). These findings indicate that breeds and breed groups with high allelic diversity and heterozygosity tend to exhibit a wide, yet partially overlapping, range of diversity, which leads to lower differentiation.

Specific pairwise comparisons of *D*_*EST*_ further illuminate the complex genetic history of these target populations. As a baseline, the differentiation between the domestic Cyprus fat-tailed (CFTCY) breed and the wild Cyprus Mouflon (OGPCY) outgroup was exceptionally high (0.610), reflecting deep evolutionary divergence. Among domestic populations, regional and historical ties are evident in the moderate differentiation between the Cyprus fat-tailed and the Local Awassi (AWSIL) (0.199), as well as between the Greek Chios (HIOGR) and the Turkish Sakiz (SKZTR) (0.198). Interestingly, the Greek Sarakatsaniko (SRKGR) and the Balkan Karakachanska (KRCMK) breeds are highly differentiated (0.258). Conversely, low differentiation between the Greek Arta (ARTGR) breed and the European East Friesian (EFRDE) (0.088) was found.

### Genetic distances and phylogenetic relationships

**Fig. 5:**
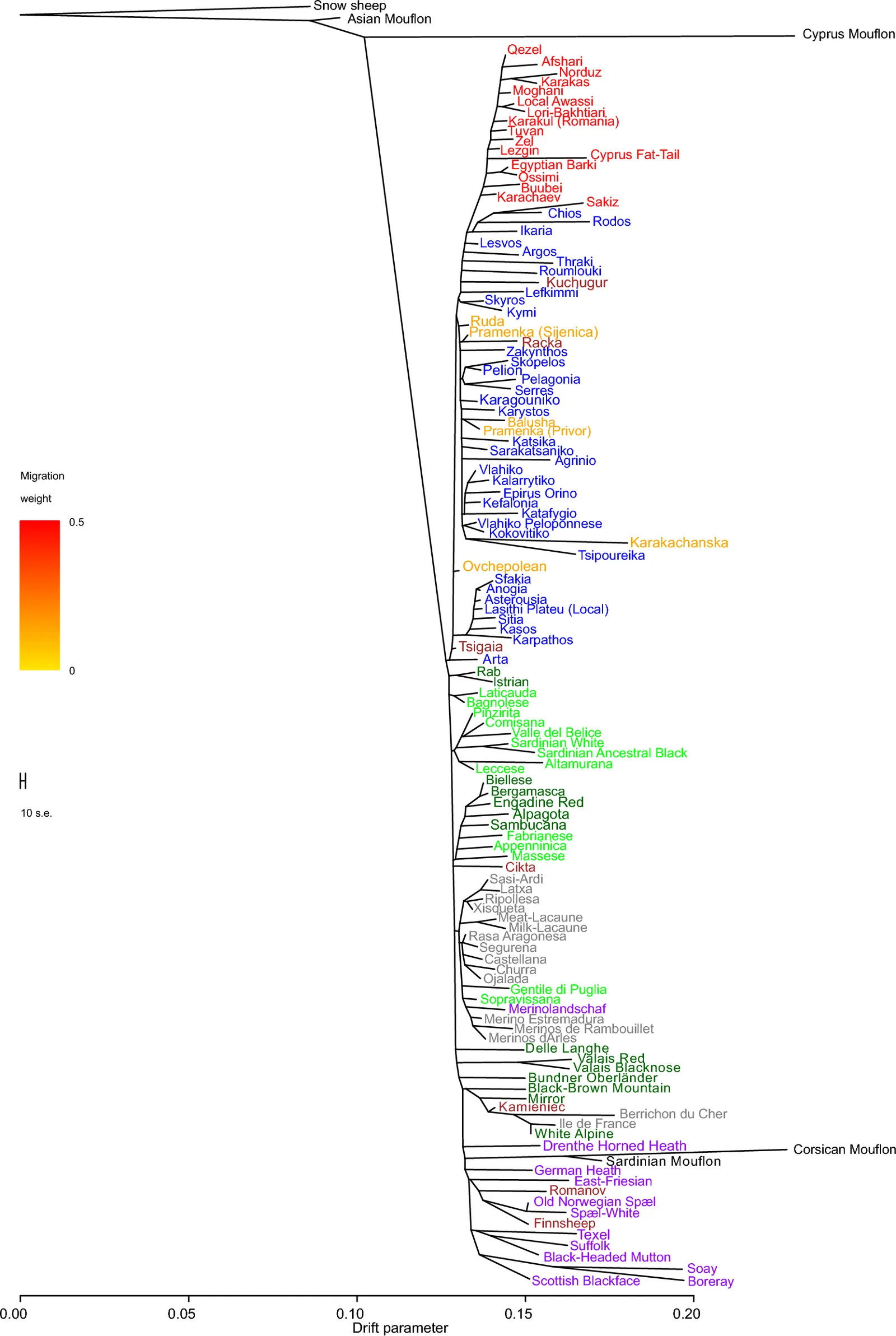
Maximum likelihood (ML) tree inferred from genome-wide allele frequency data by methods implemented in the TreeMix program. The ML dendrogram of the relationships between the 127 examined sheep populations was rooted with the Snow sheep (*Ovis nivicola*) as an outgroup

We used allele counts from bi-allelic SNPs to reconstruct a phylogeny using the *TreeMix* program (Fig. 5), with Snow sheep (*Ovis nivicola*) as the outgroup to root the tree. The resulting topology reveals a broad east–west genetic structure, with eastern sheep breeds cluster on one side of the tree and Western European breeds forming the second branch on the opposite tree side. The sheep breeds of the Balkans and Greece are positioned within a broader phylogenetic lineage that subdivides into three principal clades. At the basal portion of this lineage—hereafter referred to as the eastern clade—lies a distinct group comprising sheep from Crete, Kasos, and Karpathos. This is followed by a larger assemblage that further bifurcates into two major phylogenetic subclades. One subclade encompasses the Balkan populations together with the majority of Greek thin-tailed sheep, whereas the other includes the Near Eastern and North African lineages, in addition to four Greek semi-fat-tailed and five Greek thin-tailed breeds. Comparison of the Crete–Kasos–Karpathos Island cluster depicted in Fig. 5 with the geographic origins of these breeds shown in Fig. 2 reveals a pattern consistent with that expected by the principle of “isolation by distance”. The clustering of semi-fat-tailed Greek breeds Argos, Lesvos, Chios, and Ikaria is also, consistent with their morphological characteristics and geographic origin. The Chios also clusters with the Sakiz breed in close proximity to Ikaria. Several Greek breeds, like Lesvos, Argos, Thraki, Roumlouki, Lefkimmi, Zakynthos and more, appear as isolated branches within eastern clade. These are breeds with different characteristics, bred in diverse locations (from the East to the Western part of Greece) with different histories and development, and their distinct positions in the phylogenetic tree do not imply a similar historical evolution. Furthermore, although Lesvos also appears isolated, it is closely related to the Chios and Ikaria breeds due to geographical proximity and possible past exchanges of breeding animals. Moving westward, the majority of Greek breeds clusters closer to Balkan populations. The Tsipoureika population clusters with the Karakachanska breed, while the Pelion and Skopelos form a cluster positioned near the Serres and Pelagonia breeds. The Skyros clusters with Kymi, further supporting regional structuring among Greek sheep populations. In addition, mountain-type Greek breeds, including Vlahiko, Kalarrytiko, Epirus Orino, Kefalonia, Katafygio, Kokovitiko, and Vlahiko Peloponnese, form a coherent cluster positioned close to the Karakachanska breed. It is worth noting that the Sarakatsaniko breed was not included in this cluster and did not show a close genetic relationship to the Karakachanska breed in our analysis. The Arta breed is positioned closer to the clade encompassing Western European breeds and out of the group of the Greek breeds. The Western and Central European breeds clade shows clear geographic clustering, with Italian breeds grouping near Alpine populations, while most Spanish and French breeds cluster together in proximity to the Italian and Alpine groups. The Sardinian and Corsican mouflons are not positioned near the root of the tree like the Asian and Cyprus mouflons, but instead cluster closer to Central European domestic breeds. Finally, in this maximum likelihood tree, breeds with high levels of inbreeding tend to exhibit long branches (e.g., Karakachanska, Berrichon du Cher, Thraki), reflecting greater genetic drift. It should be noted, that the extended branch observed for the Tsipoureika population is likely amplified by its sampling structure, as this previously undocumented population was sampled from a single flock, inherently increasing the level of inbreeding. In contrast, breeds that are less differentiated and have higher genetic diversity typically display shorter or no branches (e.g. Tsigaia, Ovchepolean, Qezel, Lesvos, Anogia).

In addition, we estimated Nei’s *D*_*A*_ genetic distance based on multiallelic SNP blocks and present the distance matrix as a neighbor-net tree (Additional file 8 Fig.S3 ). The phylogenetic tree appears to be split in two, like Fig. 5, with the eastern breeds in the left half, such as the Asian (red), Balkan (orange) and Greek (blue) groups, and the western breeds in the right half, such as the “Central Europe to Southwest” group (purple). Within the Greek group, there are three main clusters. As a first point, the Cretan sheep breeds, along with the Kasos and Karpathos breeds, are close to the North African and South Italian breeds. Second, the Skyros, Kymi, Skopelos, Pelion, Karagouniko, Karystos, Kalarrytiko, Vlahiko, and Kefalonia sheep breeds are closely related to the Balkan and West Asian breeds. Finally, the third cluster is made up of the Sarakatsaniko, Kokovitiko, Vlahiko Peloponnese, Zakynthos, Katsika, Katafygio, Pelagonia, Thraki, Lefkimmi, Chios, Serres, Epirus Orino and Arta breeds, which are close to the Balkan group. This cluster includes the Turkish breed Sakiz and the fat-tailed Cypriot sheep, which are close to the Chios and Serres breeds. Moreover, as expected, the Arta breed is close to the East Friesian breed. The Tsigaia sheep breed clusters with the Balkan group. The Lesvos and Argos breeds do not cluster with any other breeds.

### Assessment of population structure using unsupervised heuristic and unsupervised model-based methods

**Fig. 6:**
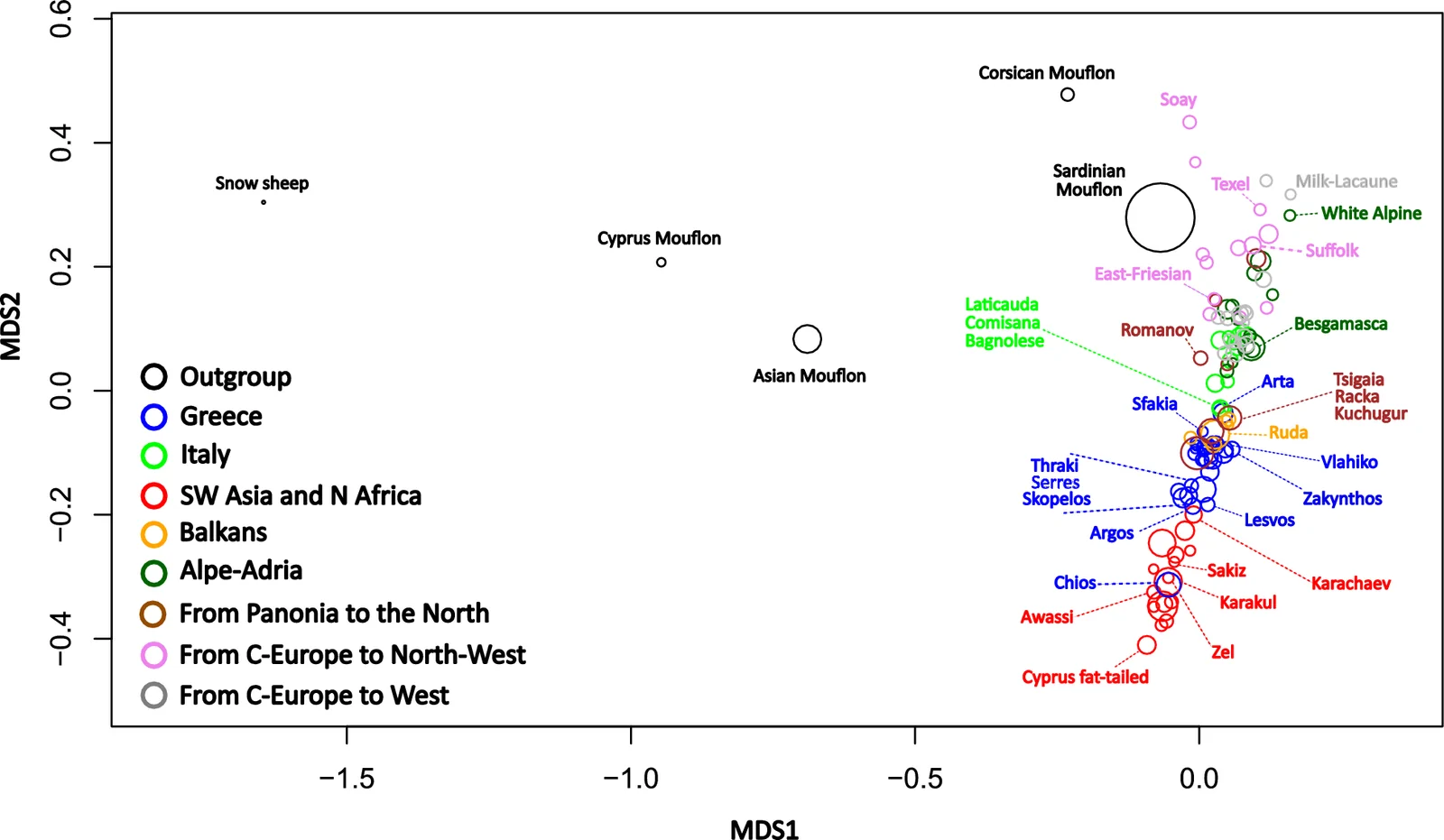
MDS projection of the estimated allele sharing distance matrix (*D*_*PS*_) among 123 breeds/populations using multi-allelic SNP blocks. The position of each breed is represented with a circle in which the center is the average position of all animals of the breed, with a radius equal to the SD (standard deviation), (C: Central, SW: Southwest, N: North)

We used multi-allelic SNP blocks to estimate the allele sharing distance matrix (*D*_*PS*_) among 2623 animals and projected these onto a two-dimensional (2D) figure using multidimensional scaling (MDS). The MDS projection of 123 breeds is shown in Fig. 6.

As illustrated in Fig. 6, the breeds/populations are categorized into eight distinct groups, alongside the Greek breeds. The plot reflects a general east-to-west gradient from bottom to top. At the lower part of the figure, the breeds of Cyprus fat-tailed and Chios are positioned close to Southwestern Asian breeds, particularly the Karakul and Zel. Slightly above them, but still near the Asian cluster, lies a group of Greek breeds: Lesvos, Argos, Lefkimmi, Thraki, Serres, Skopelos, and Karpathos. Interestingly, the Asian breed Karachaev also clusters closely with this group. Another cluster of Greek breeds includes Skyros, Pelagonia, Pelion, Kymi, Karagouniko, Sarakatsaniko, Katafygio, Vlahiko, Vlahiko of the Peloponnese, Karystos, Kefalonia, Zakynthos, Kalarrytiko, Epirus Orino, Katsika, Agrinio, and Kokovitiko. Just to the left of the cluster, the Cretan breeds, along with Kasos, form a group. The Arta breed is positioned distinctly, overlapping with three Italian breeds and located further west than the other Greek breeds. Between the Greek breeds and the Arta lie the Balkan breeds, along with Tsigaia, Racka, and Kuchugur. As we move upward in the plot (toward the west), we encounter the Italian breeds, followed by Central and Western European breeds, those North of Pannonia, and finally the Northwestern European breeds. At the farthest end, the Soay sheep appears as the most genetically distinct. The outgroup populations are positioned far from the main clusters. Notably, as in Fig. 5, the Corsican and Sardinian mouflons are closer to the Western breeds than the Asian and Cyprus mouflons which are placed nearer the Snow sheep outgroup.

**Fig. 7:**
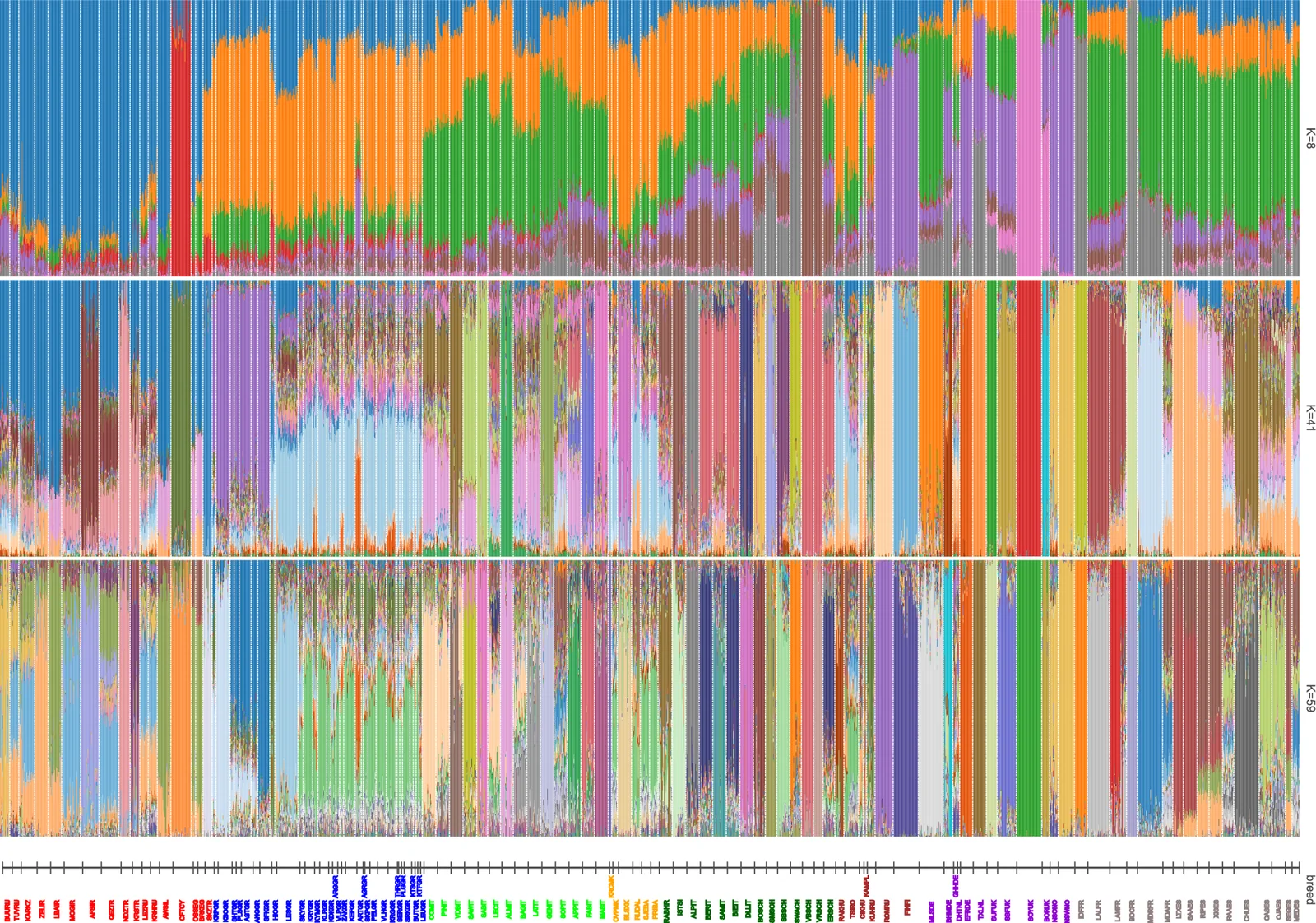
Model-based clustering of the estimated membership fractions of individuals from all 118 breeds. Each cluster is represented by a different color and each animal is shown as a vertical bar, at *K* = 8, *K* = 41 and *K* = 59. The K-value of 59 represents the lowest cross-validation error (*cv* = 0.61153), the K-value of 41 represents the onset of the CV error plateau and intermediate breed resolution, and the K-value of 8 represents the number of geographical groups. Breeds are ordered from left to right by population and group. For a full definition of the breed groups and populations see [Additional file 1 Table S1]

The admixture analysis represents the second unsupervised clustering method applied to 2299 animals using the genotypes of bi-allelic SNPs. For the Greek breeds, out of the initial 778 animals only 372 were retained for the admixture analysis (detailed sample sizes retained per breed are provided in Additional file 1 Table S1; See *N*_*ad*_ column). Here we attempted to remove any remaining family structure from our dataset in order to counteract the particular sensitivity of admixture analysis. As a result, some breeds were left with very few samples, for example, only two animals for the Thraki breed and three animals for the Argos breed. The lowest cross-validation error (*cv error* = 0.61153) was obtained at *K* = 59. We also examined *K* = 8, which corresponds to the number of geographical groups used to classify the breeds. At *K* = 8, the inferred clusters represent broad-scale ancestry components rather than detailed breed-level structure, which becomes more visible at higher *K* values. To fully illustrate the developmental progression of these admixture patterns across all levels of population subdivision, the complete set of bar plots from *K* = 2 through *K* = 59 is provided in Additional file 9 Fig. S4. Figure 7 shows the clustering patterns obtained for *K* = 8, *K* = 41 (representing the onset of the CV error plateau and intermediate breed resolution) and *K* = 59.

At *K* = 8, it is evident that the predefined geographical groups do not accurately reflect the true ancestry relationships among the breeds. The Asian breeds exhibit remarkably similar ancestry profiles, and within this group the Cyprus fat-tailed (CFTCY) breed appears to be genetically distinct, exhibiting a predominantly uniform ancestry pattern (only red). The Greek breeds share ancestry components with the Balkan breeds, while the Italian breeds show shared ancestry with the Alpine group. Overall, most European breeds exhibit similar colour patterns, which contrast with those of the Asian and African breeds. Several exceptions are noteworthy. The Soay breed (SOYUK) forms a distinct cluster. Two Alpine breeds, Valais Blacknose (VBSCH) and Valais Red (VRSCH), also form a unique cluster (predominantly brown), probably reflecting their high inbreeding levels. Furthermore, the Romanov (ROMRU) and Finnsheep (FINFI) breeds share a common ancestry pattern (with a dominant purple component), together with two Norwegian breeds (Old Norwegian Spæl (NSONO) and Spæl-White (NSWNO)), likely due to their close geographical proximity. Finally, the Île-de-France (IDFFR) and Berrichon du Cher (BDCFR) breeds also share a common ancestry component, which may be explained by their similar selection histories as meat-type breeds and their close geographical origin. At *K* = 59, the population is partitioned into 59 distinct ancestry components, allowing for a high-resolution view of breed-level genetic structure. At this level, several breeds exhibit a dominant, nearly unique ancestry component, while others display mixed and more complex patterns. Among the Greek breeds/populations, the Cretan —Sitia (SHTGR), Lasithi Plateau (PLSGR), Asterousia (ASTGR) and Anogia (ANGGR)—show similar ancestry patterns and share components primarily with Sfakia (SFKGR) breed, which appears to be the genetically most homogeneous of the Cretan breeds/populations (dominated by a strong blue component). Among the other islands of the East Aegean, Lesvos (LESGR), Chios (HIOGR) and Kasos (KSOGR) each form clearly distinguishable clusters, characterized by a dominant breed-specific component. The Karpathos (KRPGR) population also shares ancestry with Kasos population, likely reflecting their geographical proximity and potential historical gene flow. Most of the remaining Greek breeds exhibit a mixed ancestry profile, consisting of components derived from other Greek breeds, such as Lesvos breed (e.g. observed in the Argos breed—ARGGR) and Chios breed (e.g. observed in the Skopelos- SKPGR and Kymi-KYMGR), as well as from European breeds, including, for example, East-Friesian (EFRDE) breed. Moreover, mainland breeds (such as Kallarytiko, Vlahiko, Sarakatsaniko, Epirus Orino) share a relatively high proportion of ancestry with several Balkan breeds, such as Pramenka (Privor) (PRIBA) breed, Pramenka (Sijenica) (SJEBA) breed, Ruda (RUDAL) breed and Ovchepolean (OVPMK) breed. One notable exception is the Arta (ARTGR) breed, which shows a much higher proportion of ancestry shared with East-Friesian. The results are in accordance with the geographical location of the breeds/populations studied and also with the known history of breed development as in the case of Arta breed.

At *K* = *41*, the Admixture analysis revealed that the genetic background of the Greek sheep populations is primarily driven by two major ancestral components, based on their membership coefficients. The most dominant is the component characterizing the mainland Greek sheep, alongside several island breeds (encompassing almost all Greek breeds except for the Cretan/Kasos populations and the Arta breed), which accounts for an average membership coefficient of 29.7% across all Greek breeds. This cluster corresponds to a ‘Mainland/Balkan’ genetic signature, as it is maximized in continental and mountainous Greek breeds (such as Kalarrytiko-KLRGR and Karystos-KRYGR), while also being shared with neighboring Balkan populations (e.g., Karakachanska-KRCMK). The second major component accounts for an average of 23.8% of the genetic makeup of the Greek breeds and strongly defines the genetic signature of the Cretan and Kasos-Karpathos group. This component reaches its maximum proportions exclusively in these insular native breeds/populations, reaching its maximum coefficients in Sfakia (SFKGR), Kasos (KSOGR), and Asterousia (ASTGR). In addition to these primary components, highly specific minor genetic signatures were observed in distinct breeds. An ancestral component with a 4.0% overall average membership characterizes the Chios breed (HIOGR), which is also shared with the closely related Sakiz sheep (SKZTR). Another minor component (3.4% average membership) represents a Middle Eastern genetic influence; notably, among the Greek populations, this signature is uniquely maximized in the Lesvos breed (LESGR) (13.9%), reflecting the island’s geographical proximity to the Asia Minor. Furthermore, a distinct component (3.9% average membership) reflecting shared ancestry with Western Balkan populations was detected; this signature is globally maximized in breeds like Balusha and reaches the highest proportions in mountainous Greek breeds such as Vlahiko (VLHGR) and Katafygio (KTFGR). Finally, the Arta breed (ARTGR) exhibited a highly distinct profile, strongly driven by a specific component (reaching a local average of 37.0% in Arta, with a 3.3% overall Greek average) that is maximized almost exclusively in the European East Friesian (EFRDE) breed (94.9%).

At *K* = 59 value, a much clearer separation of genetically distinct breeds becomes, also, evident across all groups. Examples include Cyprus fat-tailed (CFTCY), Afshari (AFSIR) and Zel (ZELIR) within the Asian group; Altamurana (ALMIT) among the Italian breeds; and several Northern and Western European breeds such as Romanov (ROMRU), Finnsheep (FINFI), East-Friesian (EFRDE), Texel (TXLNL), Suffolk (SUFUK), Spæl-White (NSWNO), Île-de-France (IDFFR), Milk- and Meat-Lacaune (LALFR / LAMFR) and Berrichon du Cher (BDCFR).

## Discussion

This research constitutes the most comprehensive study of 36 indigenous sheep breeds/populations of Greece and one Cyprus to date compared with 86 breeds originating from Asia Minor, North Africa, and Europe, as well as four mouflon populations and Snow sheep as outgroups [39, 43, 74–84]. Sampling was carried out across the entire Greek geographic range (Fig. 2), encompassing almost all the autochthonous breeds currently raised, including several that are classified as continually at risk of extinction or not recognized officially, aiming to capture the widest possible representation of each population. While some breeds were represented by only a few individuals (Table 1), increasing the sample size was not feasible due to the small population sizes and high levels of relatedness within many herds. Although, the small sample sizes may affect the interpretation of some results, the implementation of more than one method (MDS, Admixture, and *TreeMix*) reinforces the findings and assists to a more reliable characterizations of these breeds/populations.

The findings suggest that many Greek breeds have high genetic diversity levels, which is indicative of past admixture, historically large population sizes, and varying human selection (Table 1). The results for the diversity parameters of the Greek breeds align with those of previous studies that used microsatellite markers [30, 33, 35, 38]. A few endangered Greek breeds, such as Agrinio and Thraki, exhibit reduced genetic diversity and elevated inbreeding due to recent demographic declines. However, they do not reach the severe genetic erosion seen in other Northern European isolated breeds. For instance, the Soay (SOYUK) and Boreray (BORUK) breeds, two fully isolated insular populations with small effective population sizes, show pronounced reduction in genetic diversity and elevated inbreeding [85–87]. This does not happen in Greek breeds/populations, despite their currently small effective population sizes, because they may have historically benefited from regional connectivity, transhumant pastoralism, and traditional flock management that buffered them against absolute genetic drift.

Moreover, some Greek breeds exhibit a deep ancestral variation possibly due to their geographic proximity to the domestication center. For instance, the Lesvos (LESGR) breed emerges as one of the most genetically diverse populations in our dataset, with diversity indices that rival or exceed those of highly variable Middle Eastern breeds such as the Qezel or Moghani. Similarly, the Chios breed reflects the Asian group’s pattern of harboring a high number of private alleles. This reflects its history, having been spread across mainland Greece to many different regions and flocks. Similarly, the “Southwest Asia and North Africa” group appears to represent a genetically distinct and highly diverse gene pool, as indicated by elevated observed heterozygosity and the high number of private alleles. Such diversity is consistent with the role of Southwest Asia as an important center of sheep domestication and diversification, where natural and human-mediated selection have acted over extended timescales. Also, the high expected heterozygosity and allelic richness detected in the “Central Europe to West” group suggest that substantial genetic variability has been retained despite geographical structuring. This may reflect historical admixture events, transhumance systems, or diverse breeding strategies that have promoted gene flow across regions [74]. Likewise, historical admixture and crossbreeding strategies have impacted specific Greek breeds; for example, the synthetic Arta breed’s genomic profile is the direct result of the development of Arta breed through the crossbreeding of local populations with East Friesian sheep 60 years ago [26, 40]. Theoretically, crossbreeding reduces the number of private alleles in the original breed, and to some extent in the recipient breed as well [88], so alleles formerly private to East Friesian sheep have partially replaced alleles that were once private to Arta.

Similarly, the Balkan group exhibits a rich genetic background, likely shaped by its position as a crossroad for livestock historical movement, facilitating repeated genetic exchanges and the maintenance of large effective population sizes [39, 43, 89]. This regional dynamic is illustrated by some mainland Greek breeds. For example, the Vlahiko breed (VLHGR), displayed the highest allelic richness within the Greek group and the lowest inbreeding coefficient, while its genetic diversity metrics are remarkably high—comparable to diverse Zackel types such as the Pramenka Sjenica and Tsigaia (Table 1). This is probably because the Vlahiko breed comes from two flocks with high number of individuals. Likewise, the Karagouniko breed maintained high levels of observed heterozygosity, exhibiting the highest *Ho* among Greek breeds, probably because of its broad dispersion in mainland Greece until the recent past [90]. These comparisons highlight the conservation importance and the adaptive potential of Greek sheep breeds, some of which surpass or match many breeds of Tsigai, Ruda, Zackel type, long recognized for their high diversity and resilience [91].

On the other hand, the Greek and Italian groups display considerable within-group heterogeneity coupled with low mean inbreeding levels, suggesting that traditional management practices and regional connectivity may have contributed to the preservation of genetic diversity. These populations therefore represent important reservoirs of adaptive genetic variation, reinforcing their significance for conservation and future breeding strategies under changing environmental conditions [35, 75, 92].

The effective population size (*Ne*) estimates provided insights into the demographic trajectories of the studied breeds. The trendlines indicated a consistent decline in *Ne* from the past to the present across all breeds. While current *Ne* values (*Ne*_5_) for the Greek breeds were generally moderate to low, reflecting the small-scale and fragmented nature of traditional sheep farming, specific cases, such as the island breeds Lesvos, Chios and the population of Kasos, stood out with relatively high *Ne* values, high genetic diversity and low inbreeding levels, suggesting more stable population dynamics or stronger breeding programs. Chios is the most well-known sheep of Greece, which has been continuously supported by the State and Agricultural Associations since the 1950s. This could explain the high level of relatedness among the flocks of all sampling areas of the breed (mainland and Chios Island). Although its highest *Ne*_*5*_, Chios shows a remarkably small difference between recent and historical *Ne* values, indicating long-term demographic stability rather than recent expansion or recovery from a bottleneck. At the same time, the moderate historical *Ne*_*85*_ suggests that Chios did not originate from an exceptionally large ancestral population but instead maintained a relatively stable effective population size over an extended period as previously reported [24]. Similarly, the Kasos population maintains a high recent *Ne* despite lacking official recognition as a standardized breed. Because of its geographical isolation and the consistent, traditional management practices of local farmers, the population has preserved highly uniform phenotypic characteristics. Consequently, the Kasos sheep function essentially as a de facto breed, sustaining a broad genetic base and a stable population size. Moreover, Lesvos and Lasithi Plateau, exhibited much higher historical *Ne* values followed by pronounced decline toward the present. This decline likely reflects their historically larger population sizes and subsequent recent demographic contractions, as is mentioned for Lesvos breed [40, 93], possibly driven by changes in management practices, land use, or breeding intensity (Fig. 4). Also, the large historical effective population size (*Ne*_*85*_) of Lasithi Plateau population aligns perfectly with the region’s deeply rooted pastoral tradition, as the region of Lasithi plateau sustained massive sheep flocks in the past. The subsequent demographic decline likely reflects modern shifts in agricultural practices and the contraction of traditional flock sizes. The demographic trajectories generated further by GONE (Additional file 7 Fig. S2) provide critical historical context for these observed declines. The initial reduction in *Ne* detected across most groups approximately 25–30 generations ago roughly coincides with the early twentieth century. This period was characterized by massive socio-economic upheavals, including World War I and subsequent regional conflicts, which caused widespread devastation and likely led to severe demographic bottlenecks in local livestock populations. Furthermore, the steep and accelerated decline in *Ne* observed more recently, between 5 and 15 generations ago, aligns closely with the post-war modernization and intensification of agriculture. During this era, traditional pastoral practices were rapidly altered, and many hardy, locally adapted indigenous breeds were marginalized or completely replaced by highly productive, imported commercial breeds. This widespread breed substitution heavily fragmented native sheep populations, drastically reducing their census and effective population sizes.

Additionally, Michailidou et al. [94] report that 9 Greek sheep breeds exhibit considerable genetic diversity and that LD-based *Ne* patterns reflect distinct demographic histories. However, their absolute *Ne* estimates for recent generations differ from our results in the same breeds. This may not be because of biological inconsistency, but it may reflect precise methodological differences; specifically, our analysis applied strict physical distance thresholds for linkage disequilibrium (200 kb to 9.5 Mb), a strict sample size correction, and a specific adjustment for the occurrence of mutation (α = 2.2). Together, these parameter constraints directly scale the absolute *Ne* values estimated by the algorithm. Breeds like Thraki, Agrinio and Katafygio, which have low *Ne* and high inbreeding coefficients, may be at greater risk of losing genetic variation. Comparable patterns of reduced heterozygosity and low effective population size in small, isolated sheep populations have been reported outside Greece as well: for example, the endangered Kosovar Balusha breed [76], together with the highly isolated Soay sheep and the native Czech Wallachian sheep breed [32, 86], showed some of the lowest diversity and *Ne* values among regional sheep breeds reflecting historical bottlenecks [37] and highlighting the vulnerability of such populations. Historical *Ne* trends, particularly *Ne*_85_, were higher in eastern breeds and lower in Northwestern European ones, in line with expectations based on the geographic spread from domestication center and subsequent founder effect [95]. Comparable ranges of effective population size have been reported in LD-based studies of sheep populations, with very low *Ne* values characterizing isolated breeds and higher values observed in more widely distributed or managed populations [77, 96, 97]. Similarly, our results are consistent with broader trends seen in European breeds, where higher *Ne* correlates with greater genetic diversity and lower inbreeding levels [43]. Taken together, patterns of genetic diversity and inbreeding are closely mirrored by effective population size estimates, indicating that contemporary genetic variation in Greek sheep breeds largely reflects their recent and historical demographic trajectories.

Across our dataset, the genetic structure was strongly shaped by geographic origin, underscoring the central role of spatial separation and historical connectivity in sheep population differentiation. This pattern reflects a pronounced east–west genetic gradient, a feature repeatedly observed in sheep and other domestic species [29, 39]. At the eastern end of this gradient, Asian and African breeds exhibited distinct genetic components, reflecting deeper historical divergence and region-specific adaptation. Consistent with previous studies, Balkan and Southwest Asian breeds formed clearly differentiated genetic groups, documenting deep regional structuring in livestock populations [12, 39, 98, 99]. Several thin-tailed Greek breeds showed close genetic affinities with Balkan populations, supporting shared regional ancestry and long-standing gene flow within southeastern Europe [29, 39]. Breeds from Italy showed close genetic affinities with Alpine populations, while Spanish and French breeds grouped nearby, reflecting geographical proximity and shared breeding histories [92]. Overall, the combined evidence confirms that geographic origin remains the primary determinant of genetic differentiation in sheep [29, 39].

Greek sheep breeds occupy a genetically intermediate position between Western European and Southwest Asian populations, a pattern that reflects both their geographic location and their historical role in early dispersal routes into Europe. This position is consistent with archaeological and genetic evidence for Neolithic movements from the Fertile Crescent into Europe through the Aegean, including coastal and island-hopping pathways, as well as subsequent migration through the Balkan Peninsula [3, 5, 12]. The genetic affinities observed between Greek, Balkan, and Southwest Asian breeds support the view that Greece functioned as a major transitional zone during the spread and diversification of domestic sheep. These findings align with previous genome-wide studies identifying Greek sheep as genetically intermediate between eastern and Western European populations [29, 39].

Strong genetic relationships were observed among the Cretan breeds/populations, which include both officially recognized breeds (such as Asterousia, Anogia, and Sfakia) and unrecognized local populations (Lasithi Plateau and Sitia), together with the island populations of Kasos and, to a lesser extent, Karpathos. This pronounced genetic isolation and tight clustering of the Cretan populations can be attributed to historical veterinary sanitary measures. Specifically, to prevent the spread of Malta fever (brucellosis) from mainland Greece starting around the 1960s, strict regulations were implemented that forbade the transportation of animals from the mainland to the islands [100]. This prolonged period of reproductive isolation effectively restricted external gene flow from the mainland, preserving and consolidating the distinct genetic structure of the local Cretan flocks. Within the above cluster the lowest number of private alleles combined with highest semi-private alleles in the Sfakia sheep breed (Table 1) confirmed the historical references that this breed exerted a significant influence on the other Cretan populations through west-to-east dissemination across the island [23], a pattern that is consistent with the observed genetic homogeneity among Cretan breeds and in the *TreeMix* tree (Fig. 5). The remarkable genetic proximity observed between the Lasithi Plateau and Anogia populations is a direct result of their geographical closeness. Both regions share nearly identical geomorphological and geo-climatic conditions, which historically facilitated gene flow and drove parallel adaptation, resulting in the strong morphological and genetic similarities seen between these two mountain populations today, suggesting the feasibility of a joint breeding or conservation program for both populations.

While Kasos consistently aligned with the Cretan group in all analyses, likely due to its close geographic proximity to Crete, which historically facilitated the exchange of sheep populations [26], Karpathos displayed a more complex genetic profile. This pattern suggests episodic gene flow or previously unrecognized livestock movements from mainland Greece and other Northern Aegean islands. Such discrepancies between geographic proximity and genetic affinity underscore the importance of historical animal movements in shaping contemporary breed structure and highlight the value of integrating multiple analytical perspectives when interpreting population histories.

Greek semi-fat-tailed breeds exhibited strong genetic affinities with Middle Eastern and North African populations, reflecting their shared evolutionary origins and morphological characteristics. While the Cyprus fat-tailed breed clusters clearly within the fat-tailed group, the Greek breeds Ikaria, Lesvos, Chios, and Argos display more intermediate, semi-fat-tailed characteristics, despite their traditional classification as fat-tailed. Consistent with this pattern, the close genetic relationship among Chios, Sakiz, and the Cyprus fat-tailed breed provides compelling evidence of long-standing connections across the Aegean region, in agreement with earlier genome-wide studies [33, 39, 43, 101]. Despite this shared ancestry, the Cyprus fat-tailed breed displayed a highly homogeneous genetic profile and a distinct position relative to other fat-tailed populations, likely reflecting prolonged geographic isolation on Cyprus and limited subsequent gene flow [3, 102]. Consistent with this interpretation, the Cyprus fat-tailed breed exhibited the lowest heterozygosity values and the highest inbreeding coefficient among breeds within its geographical group. In addition, both the Cyprus fat-tailed and Sakiz breeds showed the lowest effective population size estimates within the Asian group, suggesting increased susceptibility to genetic drift. Lesvos and Chios retained strong breed-specific genetic identities, whereas Argos showed evidence of admixture, with a substantial proportion of its ancestry shared with Lesvos (at the best K of admixture analysis). This result aligns with earlier microsatellite-based studies reporting a closer genetic relationship between Lesvos and Argos and their distinction from Chios [30, 31], indicating that this pattern is robust across different marker systems. Notably, the Cyprus Mouflon and the Cyprus fat-tailed breed showed no close genetic association, highlighting their distinct evolutionary trajectories despite shared having coexisted on the same island for many centuries. Mouflon populations further illustrated contrasting evolutionary histories between ancient wild lineages and feralized island populations. Cyprus and Asian mouflons clustered near the base of the phylogenetic tree, consistent with their status as ancient lineages closely related to early domestic sheep [17, 21]. In contrast, Sardinian and Corsican mouflons exhibited closer affinities to European breeds, reflecting secondary admixture during feralization [16].

Although traditional classification of Greek sheep has often relied on morphological traits such as wool type or tail conformation [23, 24], the genomic analyses revealed no clustering associated with fleece characteristics, while limited clustering was observed according to tail type. Instead, geography and historical connectivity remain the dominant drivers of population structure, in agreement with previous genome-wide studies [103]. Consistent with this pattern, the *TreeMix* phylogeny highlights the genetic distinctiveness of several traditional Greek breeds, like Roumlouki, Zakynthos and Lefkimmi breeds, that appear as isolated branches. In addition, a coherent clustering of mountain-type Greek breeds/populations is revealed. These include Vlahiko, Kalarrytiko, Epirus Orino, Kefalonia, Katafygio, Kokovitiko, and Vlahiko Peloponnese, while Tsipoureika occupies a distinct position associated with southern mountainous regions of Greece, reflecting its localized distribution and limited gene flow [23]. Although until the early twentieth century, the Sarakatsani people moved seasonally across large parts of the Balkan Peninsula, including present-day Greece, Bulgaria, and North Macedonia [104], unexpectedly, no genetic similarity between Sarakatsaniko and Karakachanska breeds, was observed. The Sarakatsaniko sheep are still found in various regions of Greece, such as Thrace, Macedonia, Epirus, and Thessaly, reflecting the widespread pastoral routes of this group and facilitating gene flow across distant areas [105]. Our findings suggest that despite this well-documented historical connectivity, subsequent geographic isolation, intense genetic drift, or distinct regional crossbreeding over the past century has changed their shared ancestral origins.

Within the Greek breeds’ genetic pool, the Arta breed emerged as genetically distinct from other indigenous populations. This divergence is best explained by its well-documented history of introgression from East Friesian sheep, which has reshaped its genomic background and driven its differentiation from other Greek breeds as a synthetic breed [40]. Specifically, this breed originated through systematic crossbreeding of the local lowland sheep populations of the Arta region with German East Friesian rams. The local lowland Arta population itself resulted from crosses between mountain types (autochthonous Epirus Orino) and several indigenous lowland breeds, mainly Karamaniko, Katsika, and Agrinio, but also breeds reflecting Italian influence such as Sardinian and Zakynthos, as well as Chios and Karagouniko [26]. The genetic proximity of Arta to some Italian dairy breeds (Comisana, Laticauda and Bagnolese) in our analysis further supports this interpretation. While specific documented introgression events between these breeds and East Friesian are not universally established in the literature, East Friesian sheep are widely recognized as a major dairy breed that has historically been used in crossbreeding programs to enhance milk production in Mediterranean sheep populations [106]. Conversely, these findings indicate that although the applied methodologies and datasets are sufficiently sensitive to detect analogous characteristics in other Greek breeds, such features are not observed, thereby supporting their genetic purity.

Conclusively, our findings align with and extend previous genetic studies on Greek and Mediterranean sheep e.g. [30, 33, 39, 94, 107], providing a more complete and less biased picture due to the use of haplotype-based diversity estimates. Especially, our study achieved a more accurate assessment of genetic diversity in underrepresented and genetically distinct breeds from Southwest Asia and the Mediterranean region. The integration of the supervised and unsupervised cluster analyses’ findings reveals a consistent narrative: Greek and Cypriot sheep breeds act as a genetic bridge between breeds of the center of domestication and those of Western Europe. This transitional status is reflected in the heterogeneous genetic profiles and elevated diversity indices of the Greek breeds.

The conservation implications of this study are crucial, suggesting that current management measures should be reconsidered in light of these genomic findings. For small, unstandardized populations that share very close genetic affinities, such as the Kokovitiko and Vlahiko Peloponnese, as well as closely related Cretan breeds, a carefully managed genetic exchange could be an effective strategy to prevent severe inbreeding depression. On the other hand, certain Greek breeds, particularly those exhibiting low *Ne* and high inbreeding, which also have high differentiation (e.g. Argos, Thraki, Agrinio, Serres) are susceptible to extinction. For these unique breeds, immediate and targeted conservation measures, such as herdbooks and active breeding programs for breeds/populations currently lacking them, are urgently required to preserve their distinct genetic heritage.

Concurrently, Greek breeds exhibiting elevated gene diversity, constitute invaluable reservoirs of genetic variation. Many studies [108–113] highlight that genetic diversity serves as a crucial resource for future breeding programs, particularly in terms of prioritizing breeds for in situ and ex situ conservation, in the face of emerging challenges such as climate change, evolving diseases, and shifting production demands. Therefore, our findings provide input to national and regional conservation strategies, while supporting the integration of genomic data into management and breeding.

## Conclusions

This study provides a comprehensive genomic baseline for indigenous Greek sheep, highlighting their high genetic diversity and complex demographic history. Despite the overall high levels of genetic diversity, substantial heterogeneity was observed among breeds, with some breeds/populations showing clear signs of reduced genetic variation and increased inbreeding. These findings underline the importance of indigenous sheep as valuable reservoirs of genetic diversity, while also emphasizing the need for targeted conservation strategies for vulnerable populations. Overall, the results provide essential guidance for prioritizing conservation actions and integrating genomic information into sustainable management and breeding programs.

## Supplementary Information

Additional file 1.

Additional file 2.

Additional file 3.

Additional file 4.

Additional file 5.

Additional file 6.

Additional file 7.

Additional file 8.

Additional file 9.

Additional file 10.

Additional file 10.

## Data Availability

The datasets used and/or analyzed during the current study are available from the corresponding author on reasonable request.
