# Supplementary material for: Unravelling the genetic structure and genetic relationships of indigenous Greek sheep breeds using SNP markers
Source: Genet Sel Evol. 2026 Aug 3;58:50. doi: 10.1186/s12711-026-01074-5 (PMC13491747; doi:10.1186/s12711-026-01074-5)
Supplement: Supplementary file 1 [file 12711_2026_1074_MOESM6_ESM.pdf]

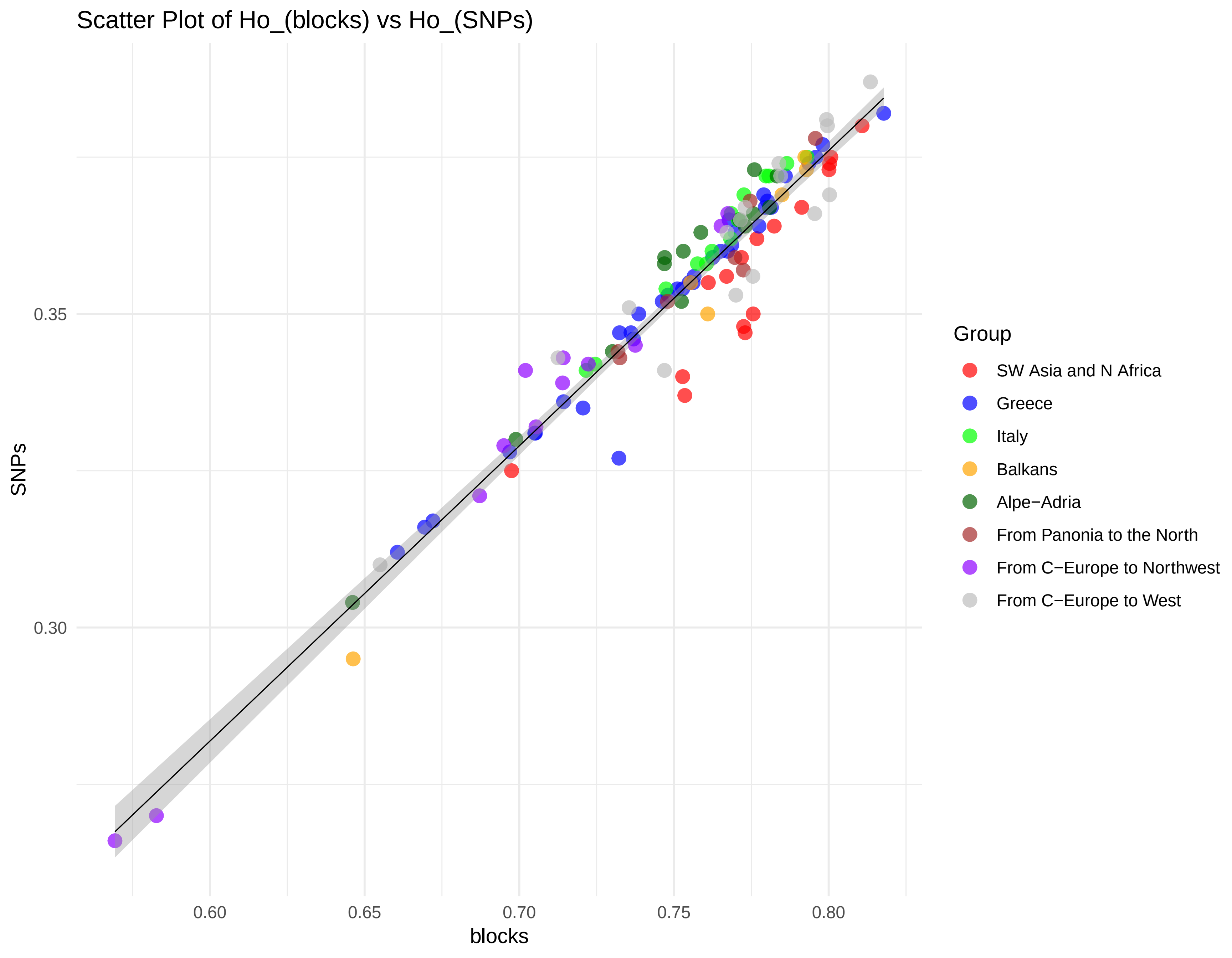
Scatter Plot of Ho_(blocks) vs Ho_(SNPs)
SNPs
0.35
0.30
0.60
0.65
0.70
0.75
0.80
blocks
Group
SW Asia and N Africa
Greece
Italy
Balkans
Alpe−Adria
From Panonia to the North
From C−Europe to Northwest
From C−Europe to West
