# Supplementary material for: Unravelling the genetic structure and genetic relationships of indigenous Greek sheep breeds using SNP markers
Source: Genet Sel Evol. 2026 Aug 3;58:50. doi: 10.1186/s12711-026-01074-5 (PMC13491747; doi:10.1186/s12711-026-01074-5)
Supplement: Supplementary file 2 [file 12711_2026_1074_MOESM7_ESM.pdf]

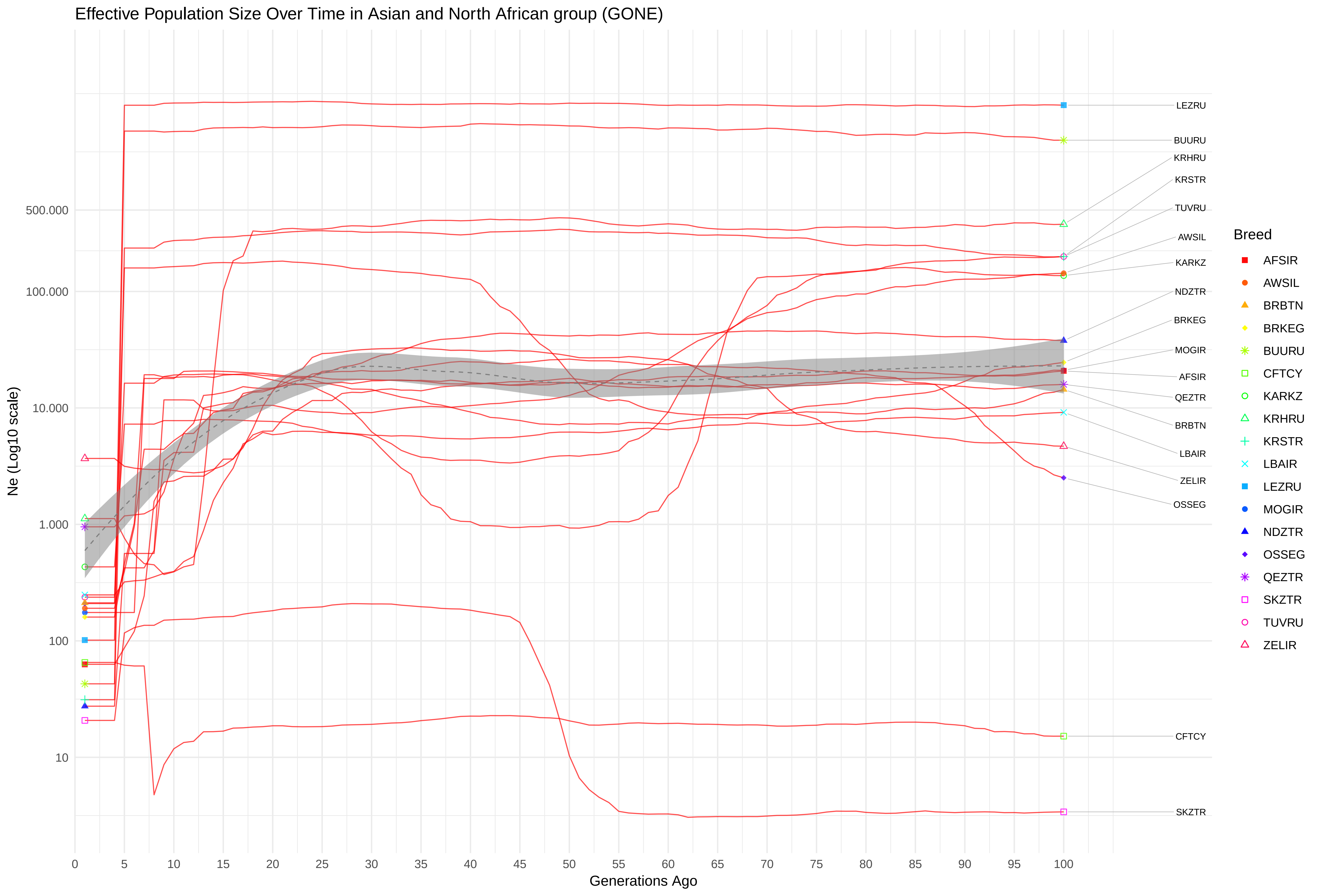

Effective Population Size Over Time in Asian and North African group (GONE)
Ne (Log10 scale)
500.000
100.000
10.000
1.000
100
10
Generations Ago
0
5
10
15
20
25
30
35
40
45
50
55
60
65
70
75
80
85
90
95
100
LEZRU
BUURU
KRHRU
KRSTR
TUVRU
AWSIL
KARKZ
NDZTR
BRKEG
MOGIR
AFSIR
QEZTR
BRBTN
LBAIR
ZELIR
OSSEG
CFTCY
SKZTR
Breed
AFSIR
AWSIL
BRBTN
BRKEG
BUURU
CFTCY
KARKZ
KRHRU
KRSTR
LBAIR
LEZRU
MOGIR
NDZTR
OSSEG
QEZTR
SKZTR
TUVRU
ZELIR

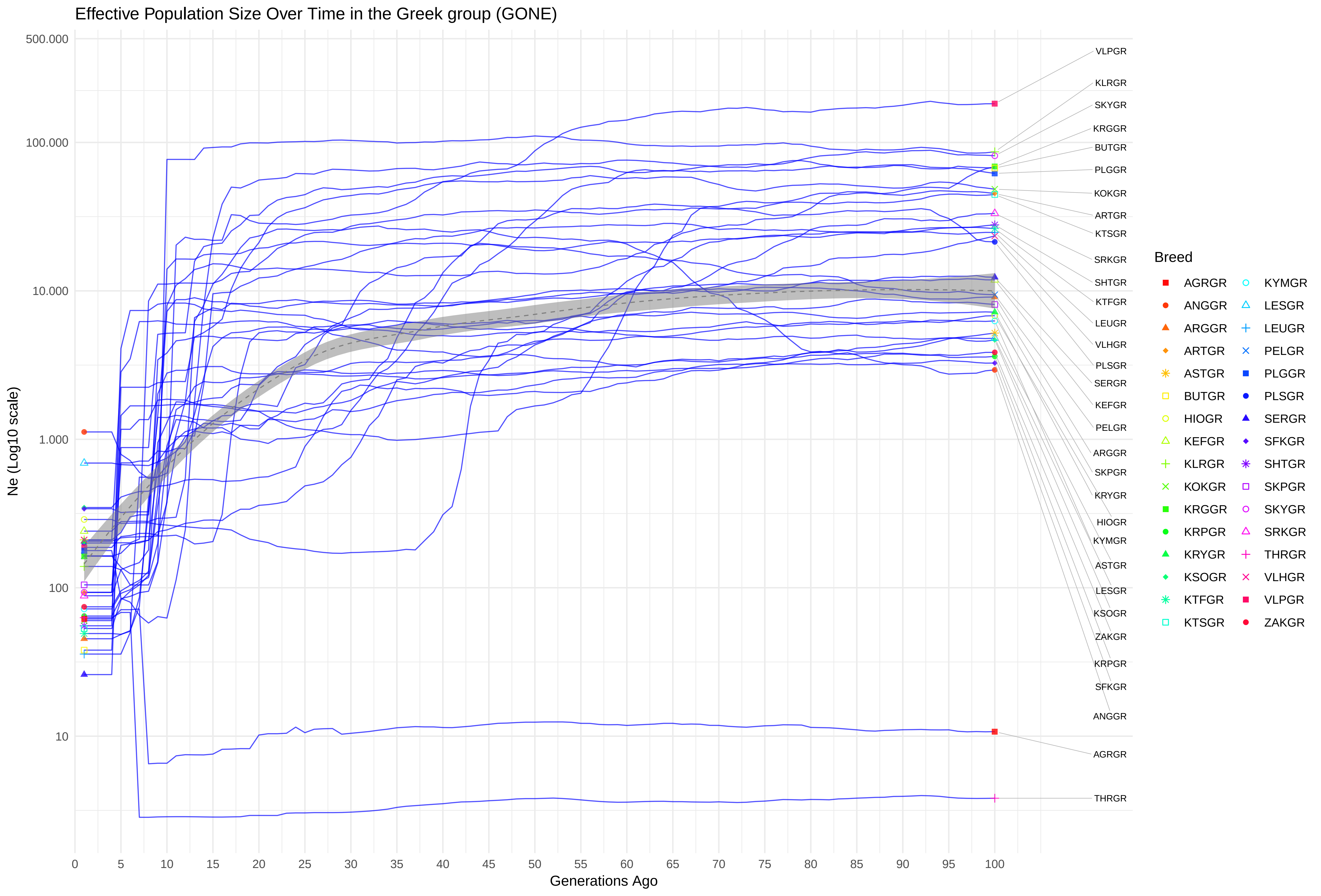

Effective Population Size Over Time in the Greek group (GONE)
Ne (Log10 scale)
500.000
100.000
10.000
1.000
100
10
Generations Ago
0
5
10
15
20
25
30
35
40
45
50
55
60
65
70
75
80
85
90
95
100
VLPGR
KLRGR
SKYGR
KRGGR
BUTGR
PLGGR
KOKGR
ARTGR
KTSGR
SRKGR
SHTGR
KTFGR
LEUGR
VLHGR
PLSGR
SERGR
KEFGR
PELGR
ARGGR
SKPGR
KRYGR
HIOGR
KYMGR
ASTGR
LESGR
KSOGR
ZAKGR
KRPGR
SFKGR
ANGGR
AGRGR
THRGR
Breed
AGRGR
ANGGR
ARGGR
ARTGR
ASTGR
BUTGR
HIOGR
KEFGR
KLRGR
KOKGR
KRGGR
KRPGR
KRYGR
KSOGR
KTFGR
KTSGR
KYMGR
LESGR
LEUGR
PELGR
PLGGR
PLSGR
SERGR
SFKGR
SHTGR
SKPGR
SKYGR
SRKGR
THRGR
VLHGR
VLPGR
ZAKGR

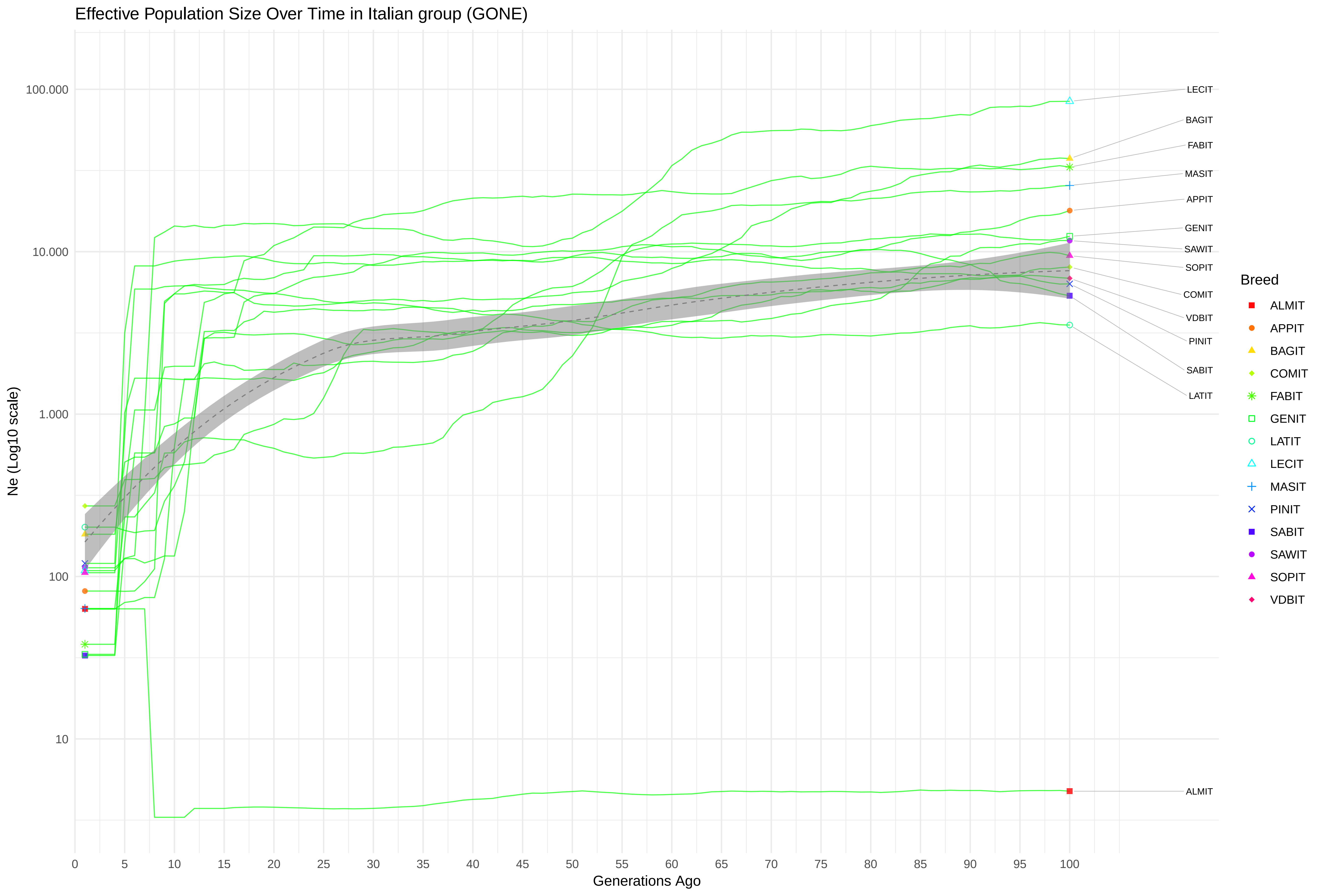

Effective Population Size Over Time in Italian group (GONE)
Ne (Log10 scale)
100.000
10.000
1.000
100
10
Generations Ago
0
5
10
15
20
25
30
35
40
45
50
55
60
65
70
75
80
85
90
95
100
LECIT
BAGIT
FABIT
MASIT
APPIT
GENIT
SAWIT
SOPIT
COMIT
VDBIT
PINIT
SABIT
LATIT
ALMIT
Breed
ALMIT
APPIT
BAGIT
COMIT
FABIT
GENIT
LATIT
LECIT
MASIT
PINIT
SABIT
SAWIT
SOPIT
VDBIT

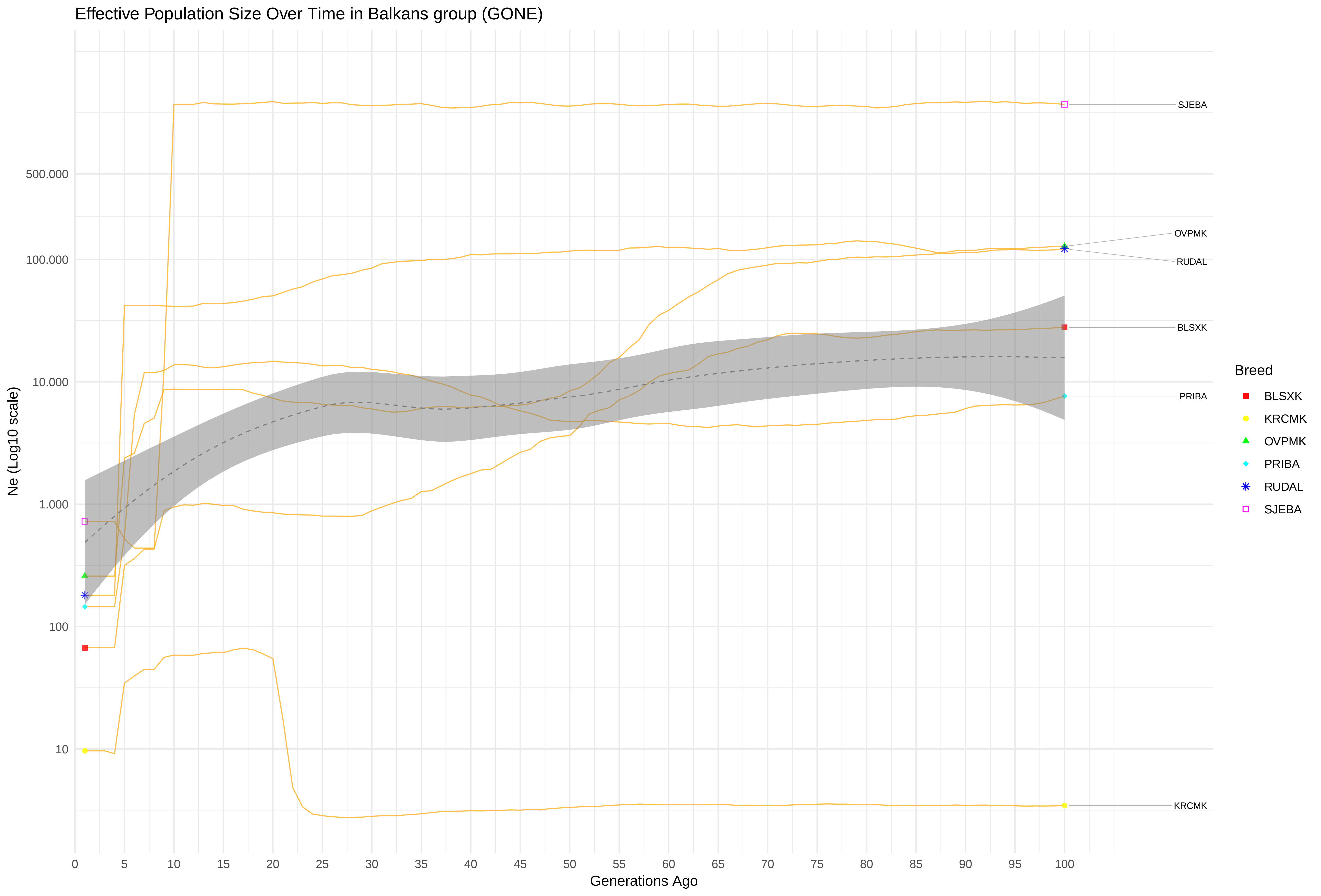
Effective Population Size Over Time in Balkans group (GONE)
Ne (Log10 scale)
Generations Ago
500.000
100.000
10.000
1.000
100
10
0
5
10
15
20
25
30
35
40
45
50
55
60
65
70
75
80
85
90
95
100
SJEBA
OVPMK
RUDAL
BLSXK
PRIBA
KRCMK
Breed
BLSXK
KRCMK
OVPMK
PRIBA
RUDAL
SJEBA

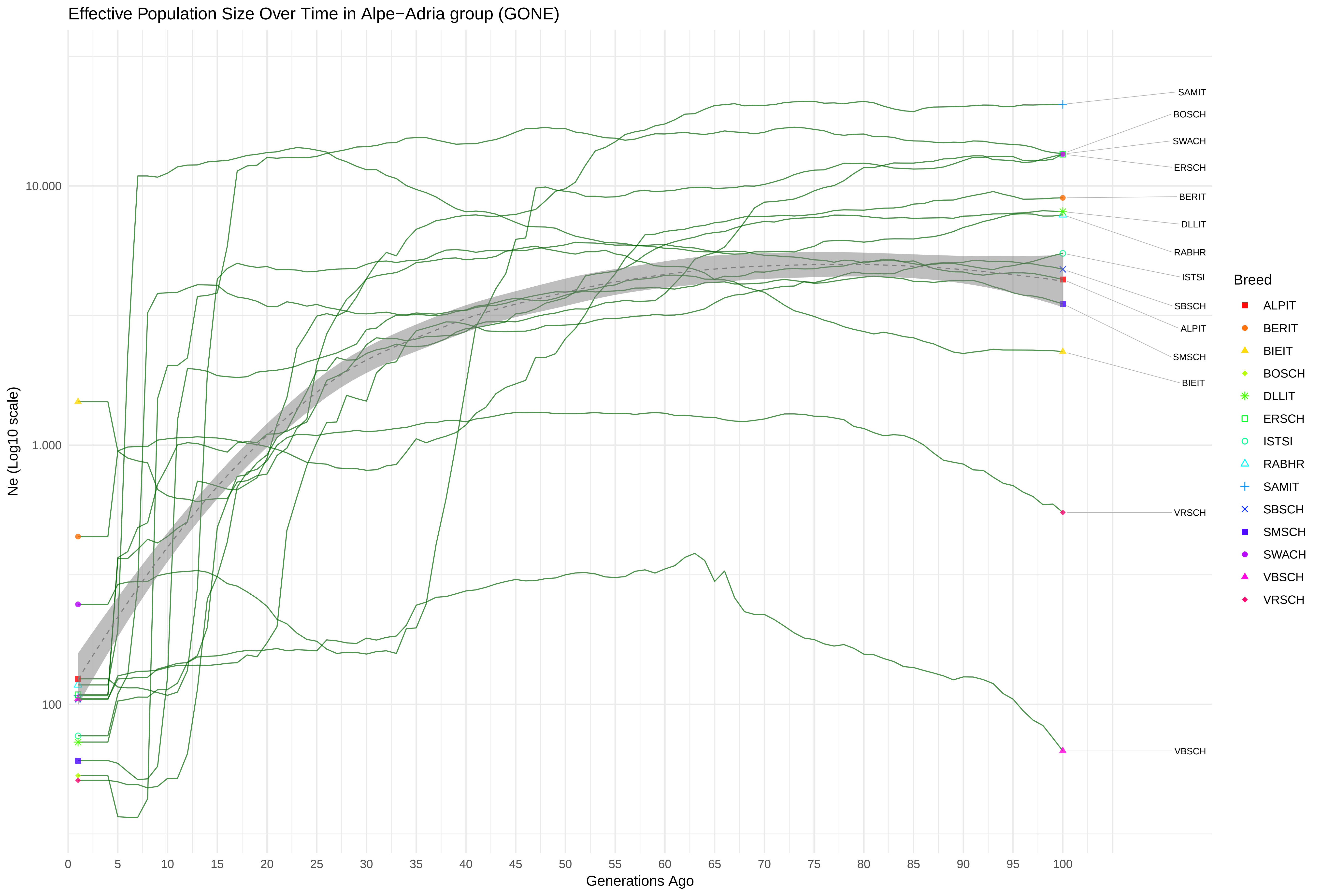

Effective Population Size Over Time in Alpe−Adria group (GONE)
Ne (Log10 scale)
10.000
1.000
100
Generations Ago
0
5
10
15
20
25
30
35
40
45
50
55
60
65
70
75
80
85
90
95
100
SAMIT
BOSCH
SWACH
ERSCH
BERIT
DLLIT
RABHR
ISTSI
SBSCH
ALPIT
SMSCH
BIEIT
VRSCH
VBSCH
Breed
ALPIT
BERIT
BIEIT
BOSCH
DLLIT
ERSCH
ISTSI
RABHR
SAMIT
SBSCH
SMSCH
SWACH
VBSCH
VRSCH

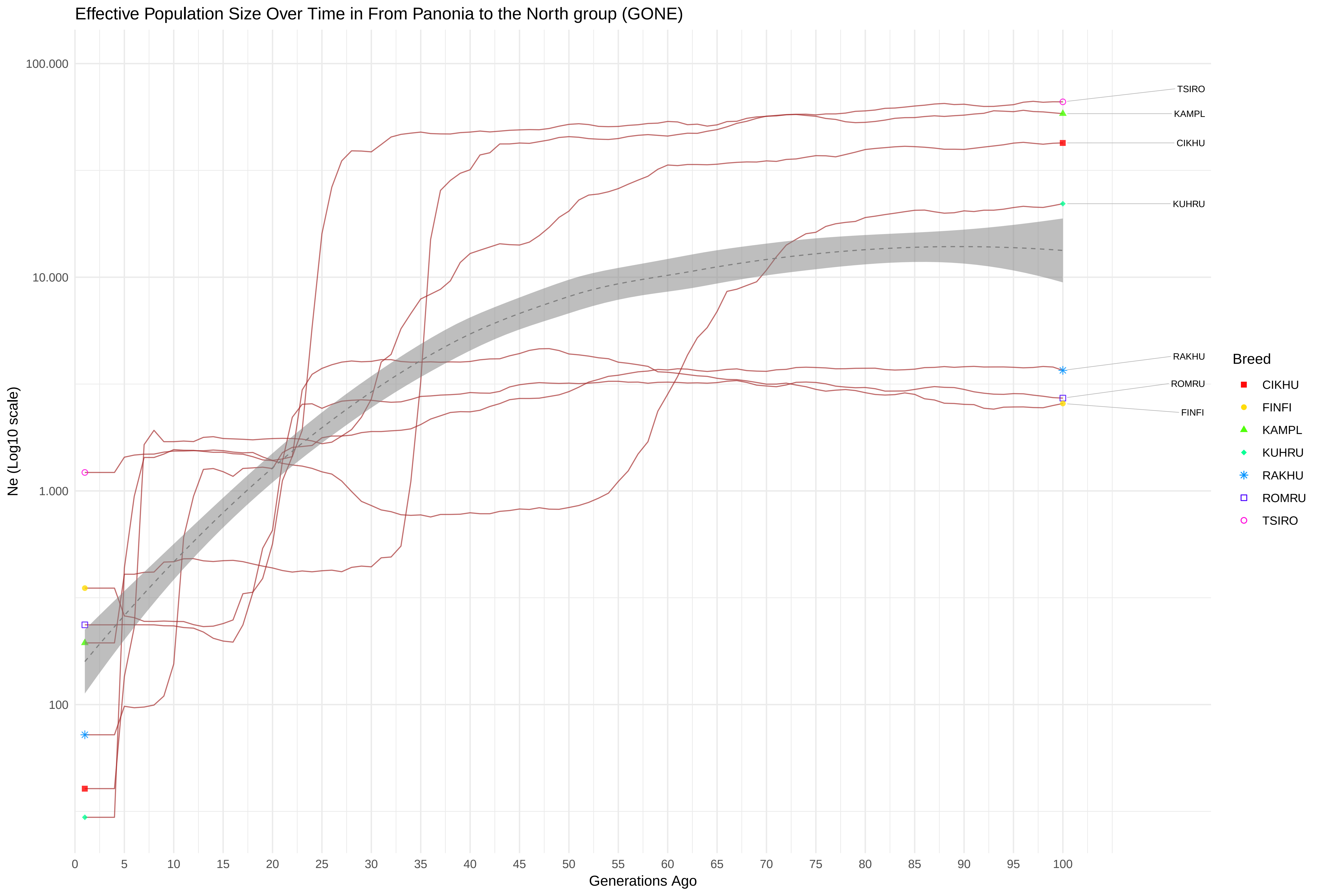

Effective Population Size Over Time in From Panonia to the North group (GONE)
Ne (Log10 scale)
100.000
10.000
1.000
100
Generations Ago
0
5
10
15
20
25
30
35
40
45
50
55
60
65
70
75
80
85
90
95
100
TSIRO
KAMPL
CIKHU
KUHRU
RAKHU
ROMRU
FINFI
Breed
CIKHU
FINFI
KAMPL
KUHRU
RAKHU
ROMRU
TSIRO

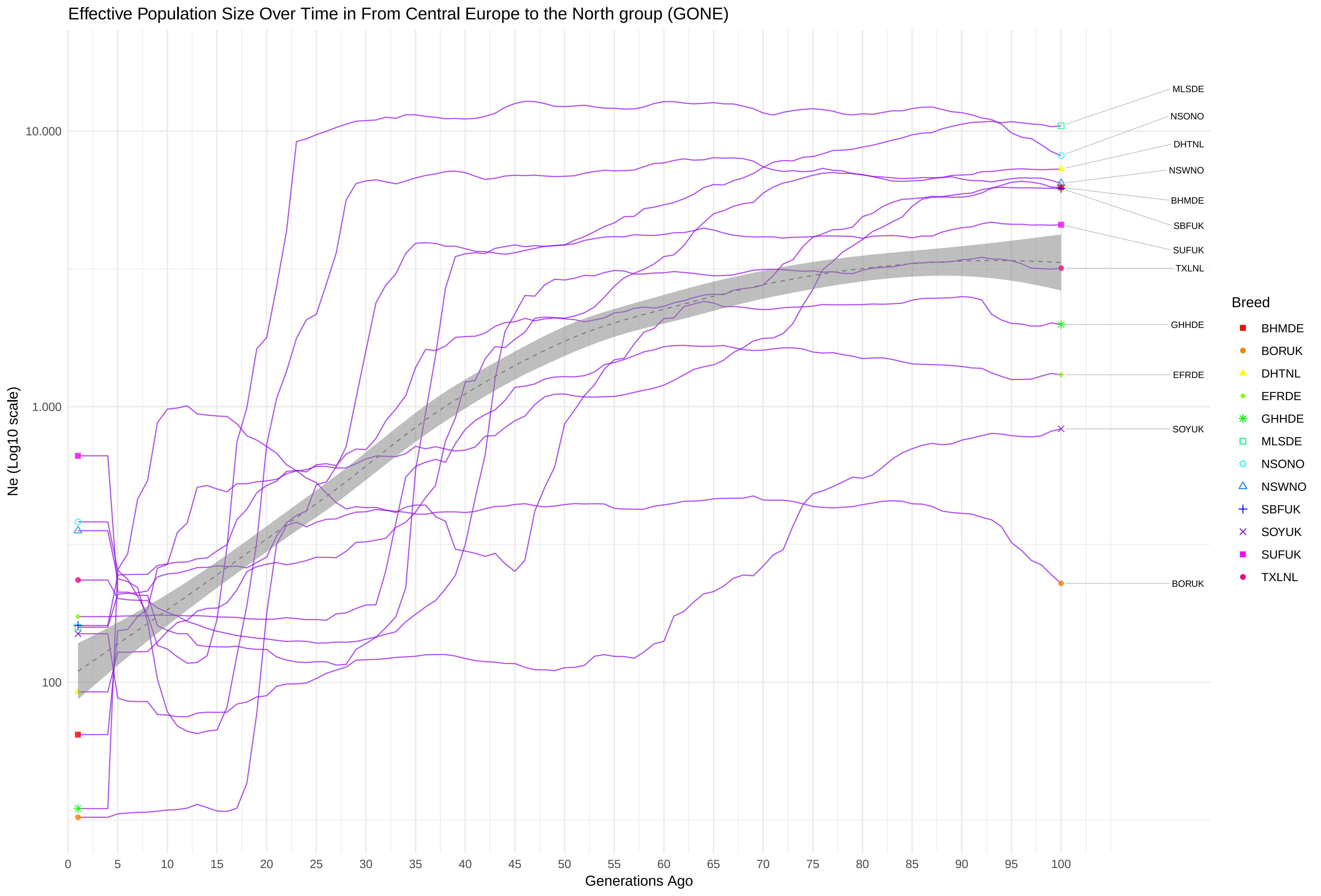

Effective Population Size Over Time in From Central Europe to the North group (GONE)
Ne (Log10 scale)
10.000
1.000
100
Generations Ago
0
5
10
15
20
25
30
35
40
45
50
55
60
65
70
75
80
85
90
95
100
MLSDE
NSONO
DHTNL
NSWNO
BHMDE
SBFUK
SUFUK
TXLNL
GHHDE
EFRDE
SOYUK
BORUK
Breed
BHMDE
BORUK
DHTNL
EFRDE
GHHDE
MLSDE
NSONO
NSWNO
SBFUK
SOYUK
SUFUK
TXLNL

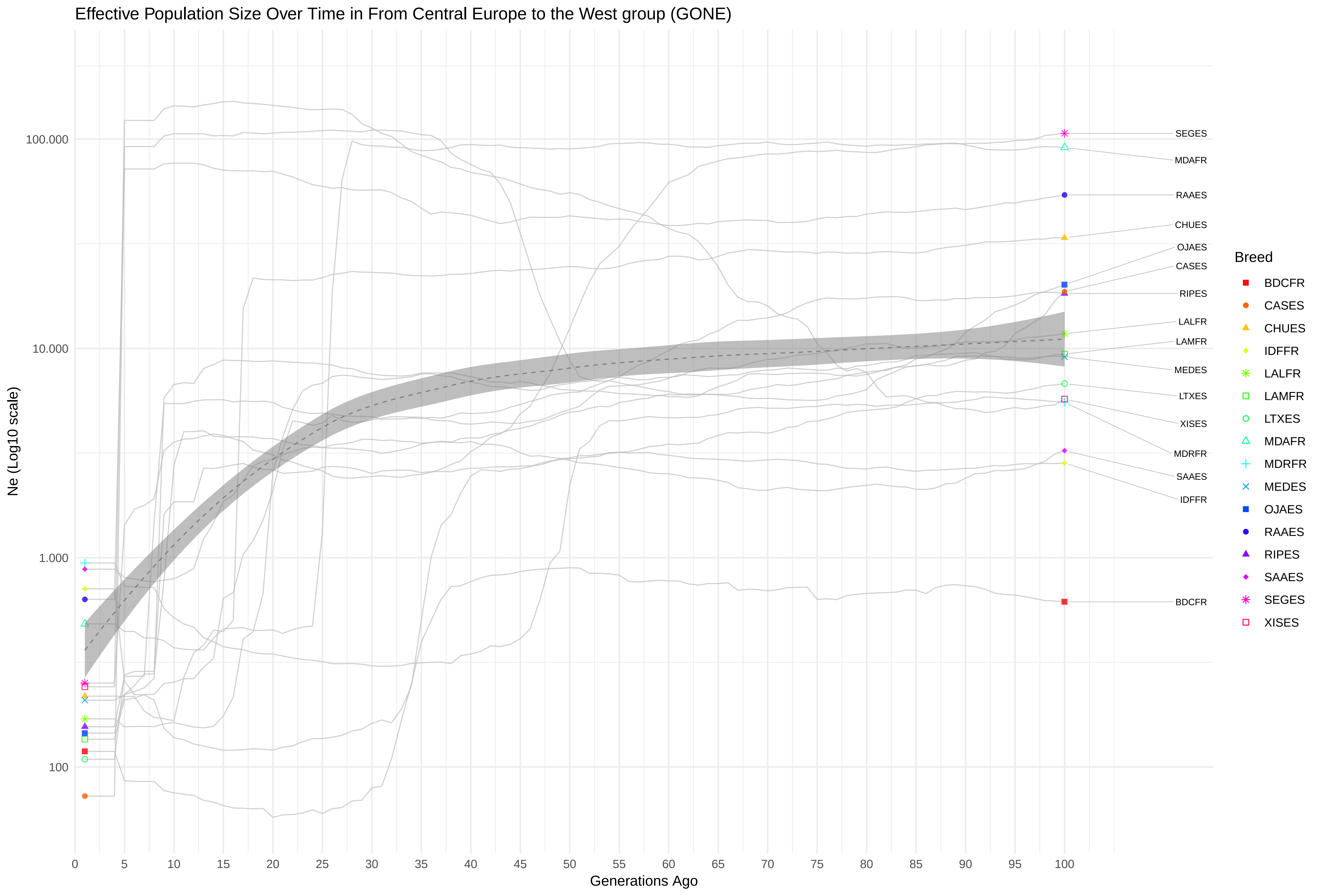
Effective Population Size Over Time in From Central Europe to the West group (GONE)
Ne (Log10 scale)
100.000
10.000
1.000
100
Generations Ago
0
5
10
15
20
25
30
35
40
45
50
55
60
65
70
75
80
85
90
95
100
SEGES
MDAFR
RAAES
CHUES
OJAES
CASES
RIPES
LALFR
LAMFR
MEDES
LTXES
XISES
MDRFR
SAAES
IDFFR
BDCFR
Breed
BDCFR
CASES
CHUES
IDFFR
LALFR
LAMFR
LTXES
MDAFR
MDRFR
MEDES
OJAES
RAAES
RIPES
SAAES
SEGES
XISES
