# Supplementary material for: Unravelling the genetic structure and genetic relationships of indigenous Greek sheep breeds using SNP markers
Source: Genet Sel Evol. 2026 Aug 3;58:50. doi: 10.1186/s12711-026-01074-5 (PMC13491747; doi:10.1186/s12711-026-01074-5)
Supplement: Supplementary file 3 [file 12711_2026_1074_MOESM8_ESM.pdf]

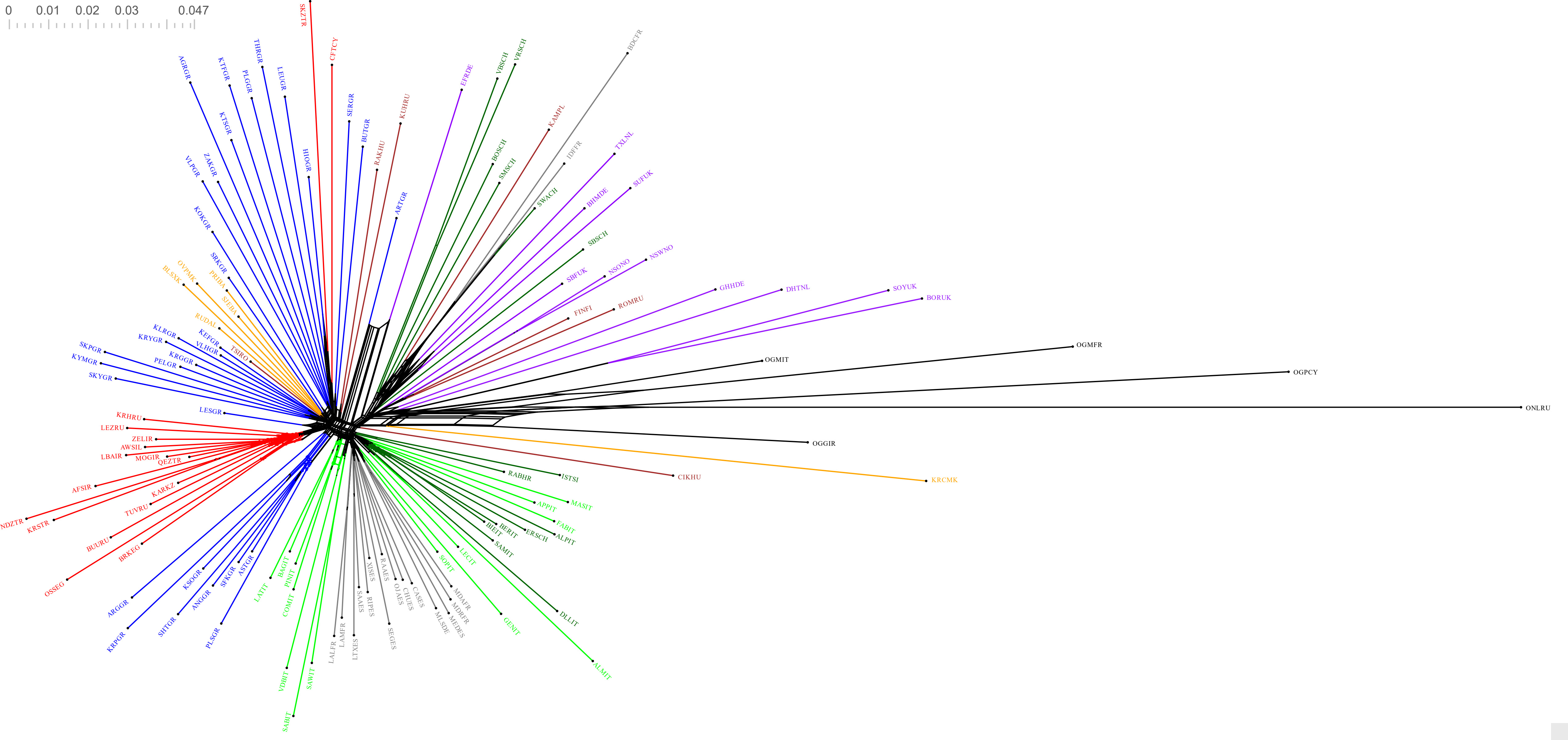
0
0.01
0.02
0.03
0.047
SKZTR
CFTCY
THRGR
LEUGR
AGRGR
KTFGR
PLGGR
KTSGR
SERGR
BUTGR
HIOGR
RAKHU
KUHRU
ARTGR
VLPGR
ZAKGR
KOKGR
SRKGR
OVPMK
BLSXK
PRIBA
SJUBA
RUDAL
KLRGR
KEFGR
KRYGR
VLHGR
TSIRO
SKPGR
KRGGR
KYMGR
PELGR
SKYGR
LESGR
EFRDE
VBSCH
VRSCH
BDCFR
KAMPL
BOSCH
IDFFR
SMSCH
TXLNL
SWACH
SUFUK
BHMDE
SBSCH
NSWNO
NSONO
SBFUK
GHHDE
DHTNL
SOYUK
BORUK
FINFI
ROMRU
OGMFR
OGMIT
OGPCY
ONLRU
OGGIR
CIKHU
KRCMK
KRHRU
LEZRU
ZELIR
AWSIL
LBAIR
MOGIR
QEZTR
AFSIR
KARKZ
TUVRU
NDZTR
KRSTR
BUURU
BRKEG
OSSEG
ARGGR
KSOGR
SFKGR
ASTGR
ANGGR
SHTGR
KRPGR
PLSGR
LATIT
BAGIT
PINIT
COMIT
VDBIT
SAWIT
SABIT
LALFR
LAMFR
LTXES
SAAES
XISES
RIPES
RAAES
OJAES
CHUES
CASES
SEGES
MLSDE
MEDES
MDRFR
MDAFR
SOPIT
LECIT
GENIT
ALMIT
DLLIT
SAMIT
BIEIT
BERIT
ERSCH
ALPIT
FABIT
APPIT
MASIT
RABHR
ISTSI
