# Supplementary material for: Unravelling the genetic structure and genetic relationships of indigenous Greek sheep breeds using SNP markers
Source: Genet Sel Evol. 2026 Aug 3;58:50. doi: 10.1186/s12711-026-01074-5 (PMC13491747; doi:10.1186/s12711-026-01074-5)

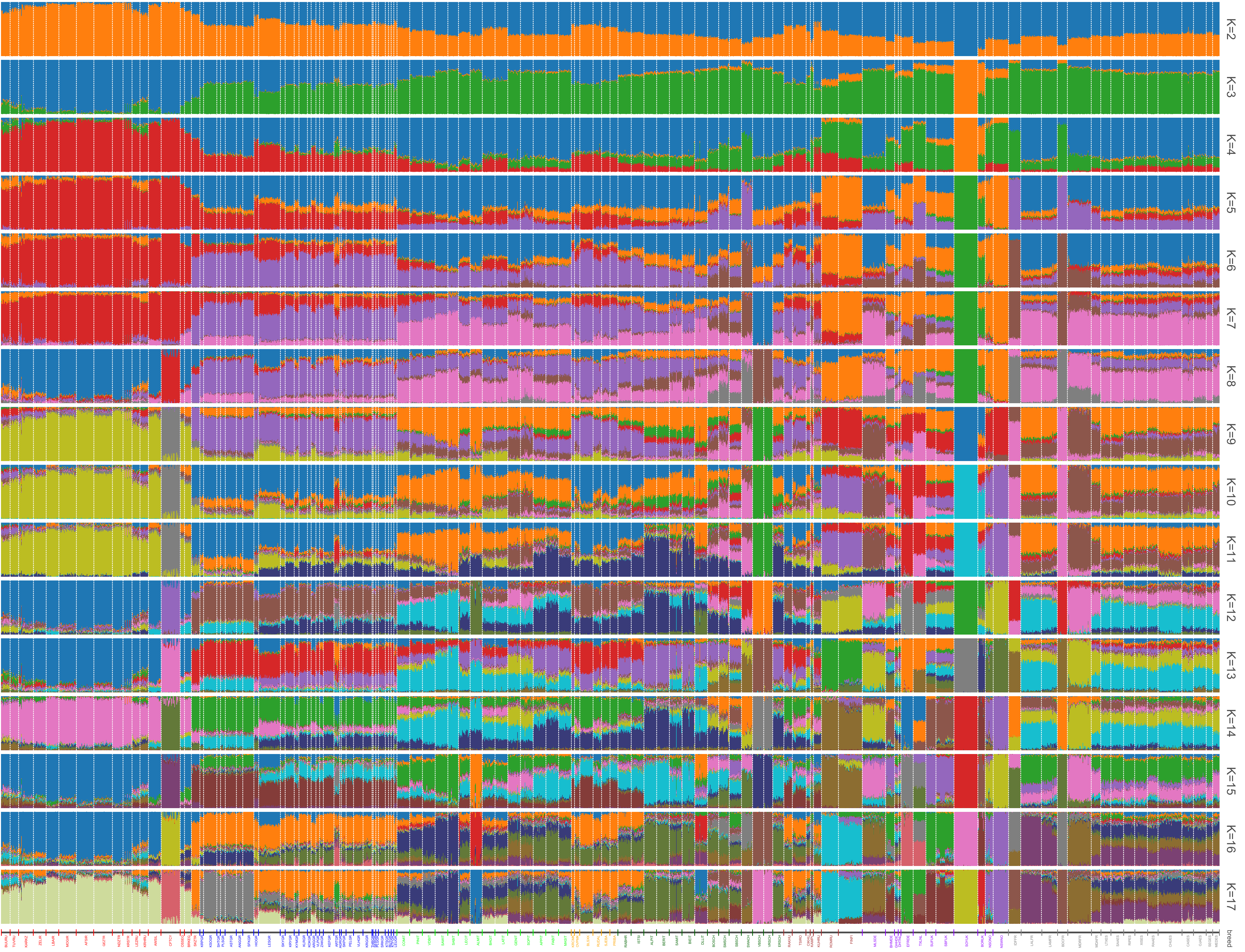
K=2
K=3
K=4
K=5
K=6
K=7
K=8
K=9
K=10
K=11
K=12
K=13
K=14
K=15
K=16
K=17
breed

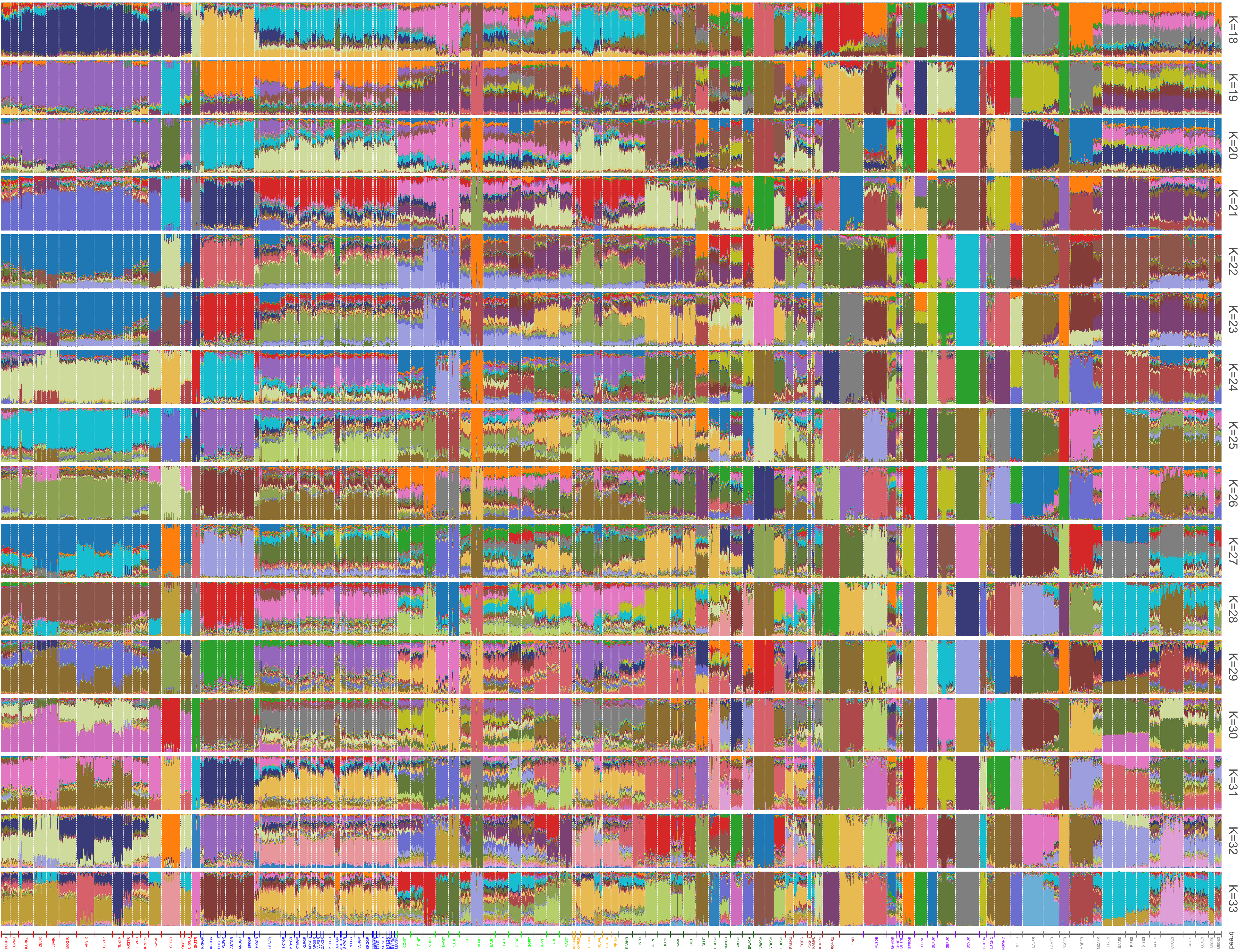
K=18
K=19
K=20
K=21
K=22
K=23
K=24
K=25
K=26
K=27
K=28
K=29
K=30
K=31
K=32
K=33
breed

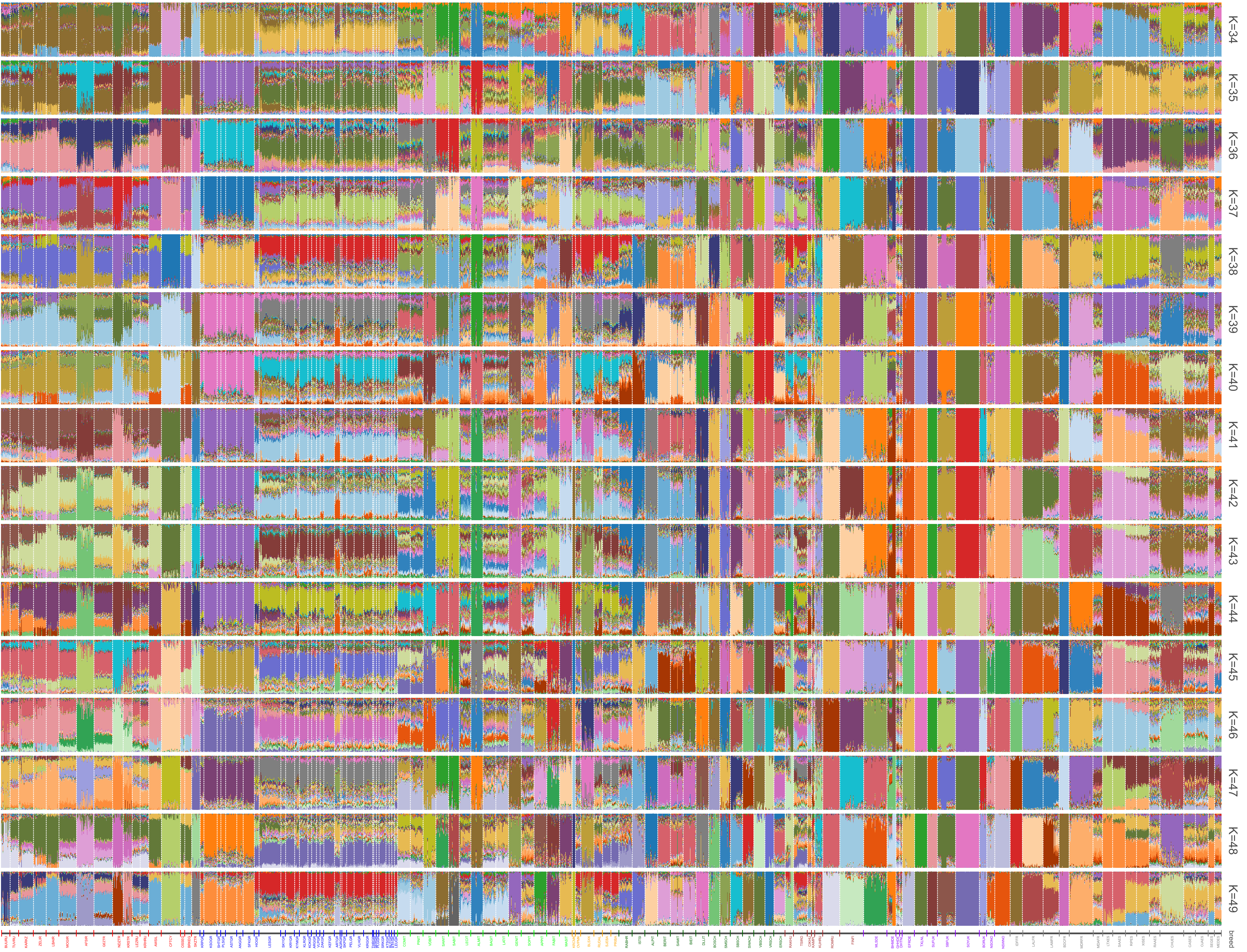
K=34
K=35
K=36
K=37
K=38
K=39
K=40
K=41
K=42
K=43
K=44
K=45
K=46
K=47
K=48
K=49
breed

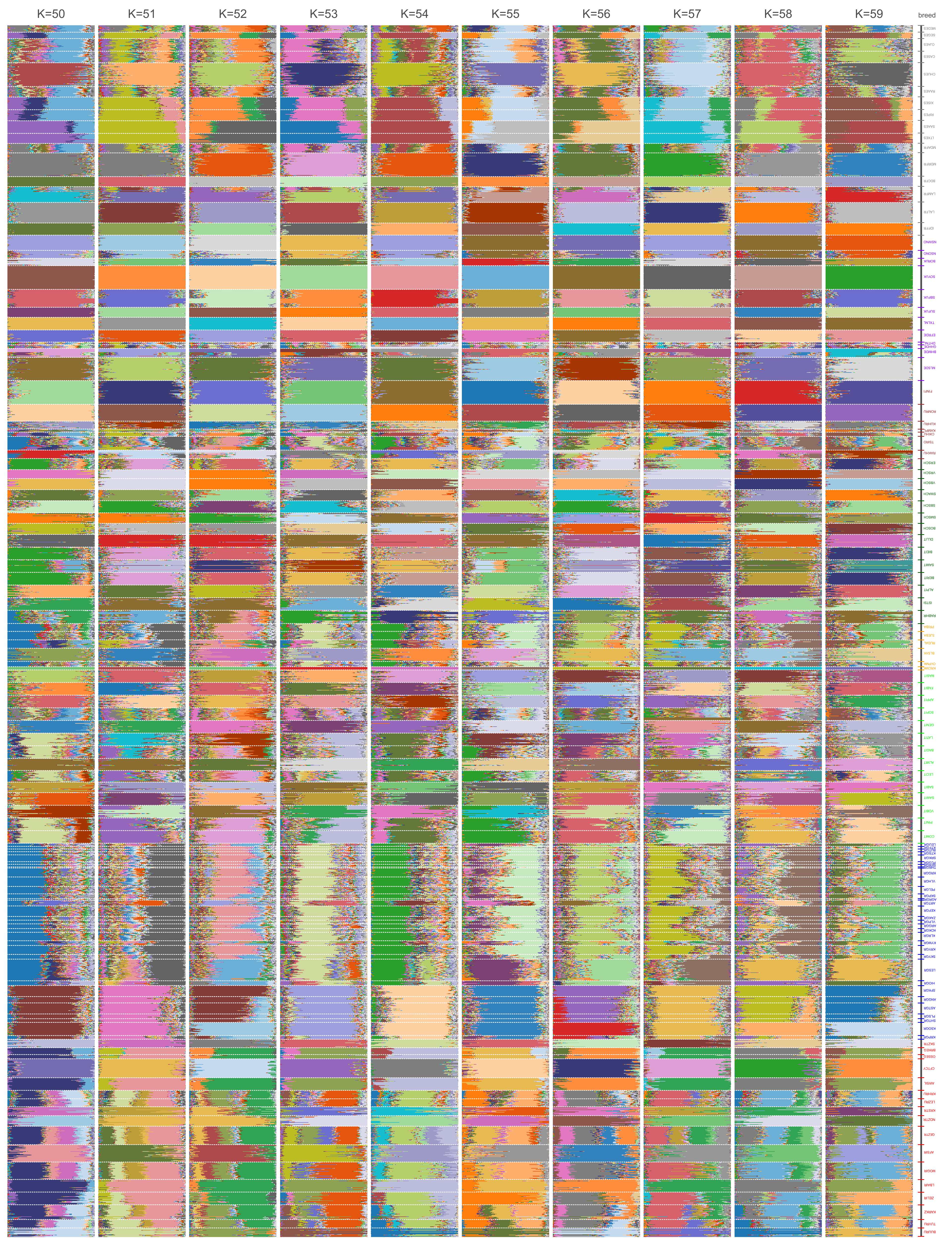
K=50
K=51
K=52
K=53
K=54
K=55
K=56
K=57
K=58
K=59
breed

ADMIXTURE CV Error (Interactive)

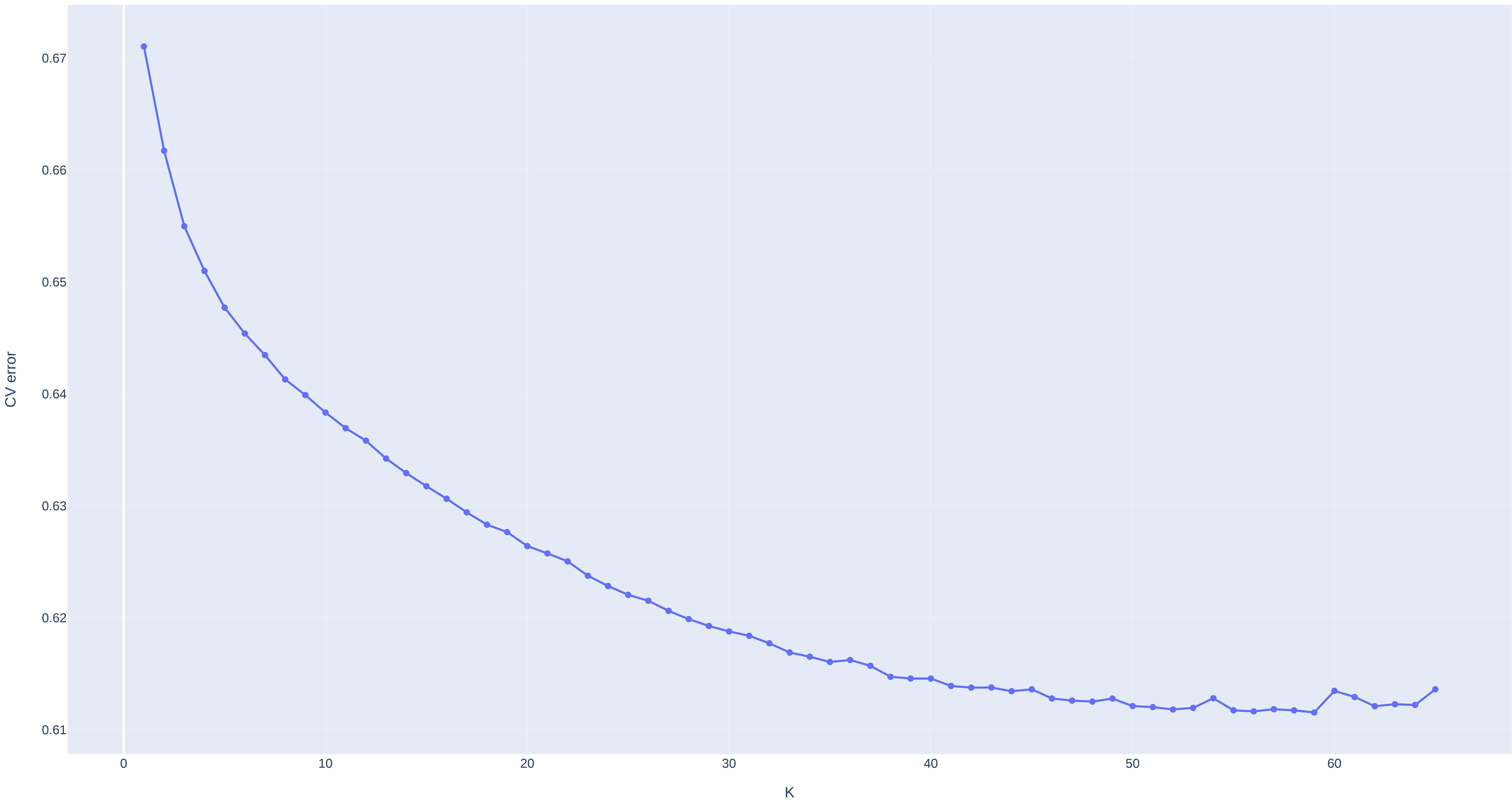

Supplement: Supplementary file 4 [file 12711_2026_1074_MOESM9_ESM.pdf]
