# Supplementary material for: Unravelling the genetic structure and genetic relationships of indigenous Greek sheep breeds using SNP markers
Source: Genet Sel Evol. 2026 Aug 3;58:50. doi: 10.1186/s12711-026-01074-5 (PMC13491747; doi:10.1186/s12711-026-01074-5)
Supplement: Supplementary file 5 [file 12711_2026_1074_MOESM10_ESM.docx]

**Additional files**

**Additional file 1 Table S1**

Format: DOCX

Title: Sample description

Description: Group allocation, RGB colour codes assigned to the pre-defined groups within this paper, breed names and codes, number of sampled and genotyped individuals, filtered sample sizes used in downstream analyses, breeding purposes, breed origin, and source of genotype data of the samples or genotypes used in this study or from previous studies [39,43,74-84]

**Additional file 1 Table S2**

Format: DOCX

Title: Phenotypic traits of each Greek sheep breed/population

Description: Phenotypic traits of Greek sheep breeds, including body weight, withers height, and body length for females and males.

**Additional file 1 Table S3**

Format: DOCX

Title: Sampling information of the Greek sheep populations included in the study

Description: Sampling dates, number of flocks, samples per flock, total number of sampled and genotyped individuals (N), sampling areas, and geographic coordinates for each Greek sheep breed/population and the Cyprus fat-tailed breed.

**Additional file 2 Table S4**

Format: DOCX

Title: Demographic and conservation status of the 36 Greek sheep breeds

Description: Population figures and information on breeding / conservation program correspond to 2023 data from the FAO Domestic Animal Diversity Information System (DAD-IS [93]). Breeds with no updated data are considered with missing or unavailable data in the registry, denoted by a dash (-). For local populations lacking official registry data (breeds 26-36), the population figures reflect the teams' expert assessments and field observations.

Note on Official Status: Following the initial data compilation for this study, the Ikaria and Lefkimmi breeds/ populations were formally granted officially recognized status by the Greek Ministry of Rural Development and Food, as published in the National Government Gazette (Ref. No. 56307/27.02.2025) [113].

**Additional file 3 Table S5**

Format: DOCX

Title: Descriptive statistics and significance testing of genetic diversity parameters across the eight predefined geographical sheep groups

Description: Values are presented as Mean ± Standard Deviation (SD) followed by the Median and 95% Confidence Interval (CI) [Lower, Upper]. Different superscript letters (a, b, c, d) within the same column indicate statistically significant differences (p < 0.05) based on pairwise comparisons with Bonferroni correction. Lack of superscript letters in a column indicates no significant differences across any groups. Ho: observed heterozygosity; He: expected heterozygosity; F: inbreeding coefficient; AR: allelic richness; Ne_5_: effective population size 5 generations ago (*SNeP* values). (*Note: The lower bound of the 95% CI for the 'From Panonia to the North' group's Ne_5_ was mathematically calculated as -0.34 but is reported as 0.00, as population size cannot be negative).

**Additional file 4 Table S6**

Format: DOCX

Title: Effective population size *N_e_* estimates at specific past generations estimated by GONE

Description: Effective population size *N_e_* values estimated by the GONE software at 5, 50, 85, and 100 generations ago for all 123 analysed sheep breeds and ovine populations.

**Additional file 5 Table S7**

Format: XLSX

Title: Pairwise genetic differentiation index

Description: Pairwise genetic differentiation among sheep populations per analysed breed, estimated using *G_ST_* and *D_EST_* values. *G_ST_* values are shown above the diagonal, while *D_EST_* values are shown below the diagonal of the matrix. Within each predefined group, maximum and minimum values are highlighted in bold and enclosed in red and green rectangles, respectively. Across all 123 breeds, overall maximum and minimum values are underlined and shown in red and green italics, respectively.

**Additional file 6 Figure S1**

Format: PDF

Title: Value distribution plot of observed heterozygosity

Description: Scatter plot of observed heterozygosity estimated based on multi-allelic SNP-blocks (*H_O_*) and bi-allelic SNPs (*H_O[SNP]_*).

**Additional file 7 Figure S2**

Format: PDF

Title: Plot of the Effective Population size (*N_e_*) over the past 100 for the 8 geographical groups of sheep breeds with trendlines highlighting demographic trajectories estimated by GONE.

Description: The names of the breed groups correspond to their geographical distributions as illustrated in Figure 2. For a full definition of the specific breeds comprising each group, please refer to Additional file 1: Table S1.

**Additional file 8 Figure S3**

Format: PDF

Title: Neighbour-network

Description: Neighbour-network based on pairwise Nei’s D_A_ genetic distances among 123 breeds.

**Additional file 9 Figure S4**

Format: PDF

Title: Model-based clustering of the estimated membership fractions of individuals from all 118 breeds

Description: Detailed visualization of the unsupervised model-based clustering (ADMIXTURE) progression for values of K = 2 through K = 59. The final panel includes a cross-validation (CV) error plot for all runs evaluated up to K = 65, demonstrating the optimal clustering resolution at K = 59.
