# Supplementary material for: Unravelling the genetic structure and genetic relationships of indigenous Greek sheep breeds using SNP markers
Source: Genet Sel Evol. 2026 Aug 3;58:50. doi: 10.1186/s12711-026-01074-5 (PMC13491747; doi:10.1186/s12711-026-01074-5)
Supplement: Supplementary file 6 [file 12711_2026_1074_MOESM11_ESM.docx]

**Table S6 ~~S7~~ Effective population size *N_e_* estimates at specific past generations estimated by GONE**

| **Ovine Population** | **Code** | ***N_e5_*** | ***N_e50_*** | ***N_e85_*** | ***N_e100_*** |
| --- | --- | --- | --- | --- | --- |
| Snow sheep | ONLRU | 37601.4 | 2497030 | 2206680 | 1898730 |
| Asian Mouflon | OGGIR | 3252770 | 3559430 | 3299100 | 3206260 |
| Cyprus Mouflon | OGPCY | 116.316 | 813.668 | 724.706 | 557.96 |
| Sardinian Mouflon | OGMIT | 12151.7 | 6478.14 | 5153.61 | 3213.13 |
| Corsican Mouflon | OGMFR | 51.8437 | 2785.72 | 2920.79 | 2057.96 |
| Buubei | BUURU | 2382100 | 2635720 | 2204020 | 1989900 |
| Tuvan | TUVRU | 16309.6 | 3891.42 | 178253 | 199289 |
| Karakul (Romania) | KARKZ | 7258.06 | 931.501 | 158686 | 136793 |
| Zel | ZELIR | 3154.49 | 27981.3 | 5807.14 | 4690.59 |
| Lori-Bakhtiari | LBAIR | 320.611 | 6201.41 | 8315.1 | 9159.61 |
| Moghani | MOGIR | 175.201 | 16472 | 20062.6 | 21037.6 |
| Afshari | AFSIR | 87.026 | 16428 | 17852.5 | 20767.7 |
| Qezel | QEZTR | 1182.77 | 17911.9 | 16017.8 | 15914.2 |
| Norduz | NDZTR | 563.017 | 41432.2 | 42411.2 | 37838.8 |
| Karakas | KRSTR | 235912 | 340128 | 248065 | 199370 |
| Lezgin | LEZRU | 3971070 | 4137220 | 3977080 | 3975660 |
| Karachaev | KRHRU | 763.277 | 427406 | 354662 | 377395 |
| Local Awassi | AWSIL | 423.443 | 12798.5 | 112718 | 143364 |
| Cyprus Fat-Tail | CFTCY | 61.8365 | 20.5798 | 20.0501 | 15.2031 |
| Ossimi | OSSEG | 394.284 | 26248.6 | 16489.9 | 2511.4 |
| Egyptian Barki | BRKEG | 455.368 | 7331.15 | 13217.4 | 24596.7 |
| Sakiz | SKZTR | 116.929 | 10.3078 | 3.41379 | 3.40163 |
| Karpathos | KRPGR | 84.454 | 2319.78 | 3504.56 | 3619.62 |
| Kasos | KSOGR | 408.143 | 2100.81 | 4111.64 | 4669.03 |
| Sitia | SHTGR | 4090.52 | 5223.28 | 24600.5 | 27843 |
| Lasithi Plateu (Local) | PLSGR | 200.837 | 35079 | 34406.8 | 21380.7 |
| Asterousia | ASTGR | 2843.97 | 3646.41 | 3866.82 | 5195.05 |
| Anogia | ANGGR | 791.85 | 22947.2 | 3695.54 | 2934.68 |
| Sfakia | SFKGR | 321.098 | 2818.8 | 3180.44 | 3263.6 |
| Chios | HIOGR | 271.08 | 5268.74 | 6069.25 | 6804.9 |
| Lesvos | LESGR | 675.199 | 5690.85 | 5031.9 | 4795.99 |
| Skyros | SKYGR | 693.572 | 29429.4 | 85805.9 | 81345.3 |
| Karystos | KRYGR | 216.742 | 6325.2 | 6533.89 | 7186.38 |
| Kymi | KYMGR | 91.689 | 4572.51 | 6016.82 | 6275.49 |
| Kalarrytiko | KLRGR | 131.887 | 110565 | 88429.5 | 86435.5 |
| Kokovitiko | KOKGR | 2241.9 | 54629.5 | 51100 | 48430.9 |
| Argos | ARGGR | 48.4184 | 8805.2 | 10009.2 | 9089.46 |
| Vlahiko Peloponnese | VLPGR | 221.108 | 87812.3 | 170573 | 182292 |
| Zakynthos | ZAKGR | 95.2799 | 2843.77 | 3760.68 | 3852.97 |
| Kefalonia | KEFGR | 278.739 | 9452.64 | 11777.3 | 11847.5 |
| Arta | ARTGR | 1167.97 | 1677.63 | 46372 | 45307 |
| Agrinio | AGRGR | 71.1381 | 12.4268 | 11.0308 | 10.7306 |
| Skopelos | SKPGR | 193.249 | 4430.25 | 8614.93 | 8098.08 |
| Pelion | PELGR | 137.182 | 19641.5 | 11079.7 | 9420.76 |
| Vlahiko | VLHGR | 235.576 | 8956.83 | 16803.9 | 23437.9 |
| Karagouniko | KRGGR | 170.962 | 13056.1 | 45559.5 | 68718.5 |
| Thraki | THRGR | 68.1729 | 3.79588 | 3.8242 | 3.81951 |
| Serres | SERGR | 1445.68 | 4322.27 | 11333.9 | 12353.5 |
| Pelagonia | PLGGR | 84.8524 | 71759.1 | 67203.2 | 61851.6 |
| Sarakatsaniko | SRKGR | 132.413 | 6084.56 | 27638.1 | 33247.8 |
| Katsika | KTSGR | 82.5185 | 30424.2 | 39469.4 | 44577.4 |
| Epirus Orino | BUTGR | 879.739 | 64756.1 | 68224.6 | 67165 |
| Katafygio | KTFGR | 48.552 | 18681.8 | 25030.2 | 26263.6 |
| Lefkimmi | LEUGR | 35.7403 | 26549.3 | 24736 | 24751.2 |
| Comisana | COMIT | 395.222 | 3174.21 | 6395.95 | 8045.75 |
| Pinzirita | PINIT | 3167.31 | 9228.39 | 7309.69 | 6338.19 |
| Valle del Belice | VDBIT | 69.2332 | 3748.04 | 5916.72 | 6860.42 |
| Sardinian White | SAWIT | 128.715 | 3049.52 | 7714.94 | 11710.6 |
| Sardinian Ancestral Black | SABIT | 160.257 | 10059.7 | 9735.94 | 5354.83 |
| Leccese | LECIT | 129.532 | 12114.7 | 65823 | 84331.5 |
| Altamurana | ALMIT | 63.2114 | 4.74157 | 4.84347 | 4.765 |
| Bagnolese | BAGIT | 506.339 | 5586.7 | 29674.9 | 37466.4 |
| Laticauda | LATIT | 192.26 | 3680.22 | 3209.71 | 3535.04 |
| Gentile di Puglia | GENIT | 1018.73 | 6110.01 | 12564 | 12438.1 |
| Sopravissana | SOPIT | 745.323 | 4720.78 | 7983.66 | 9453.44 |
| Appenninica | APPIT | 81.0657 | 9374.24 | 12187.3 | 17915 |
| Fabrianese | FABIT | 338.094 | 22616.6 | 32193.1 | 33201.4 |
| Massese | MASIT | 232.825 | 2282.06 | 23246.8 | 25581 |
| Karakachanska | KRCMK | 34.5238 | 3.33107 | 3.46411 | 3.45295 |
| Ovchepolean | OVPMK | 2379.59 | 8447.33 | 108693 | 127942 |
| Balusha | BLSXK | 315.91 | 3639.41 | 25721 | 27832.1 |
| Ruda | RUDAL | 42054.8 | 117068 | 123628 | 122190 |
| Pramenka (Sijenica) | SJEBA | 520.058 | 1791030 | 1877300 | 1848810 |
| Pramenka (Privor) | PRIBA | 545.684 | 4725.09 | 5286.71 | 7643.6 |
| Rab | RABHR | 192.413 | 6607.92 | 6243 | 7738.74 |
| Istrian | ISTSI | 109.612 | 3896.93 | 5011.84 | 5495.08 |
| Alpagota | ALPIT | 116.598 | 5927.46 | 5136.23 | 4358.07 |
| Bergamasca | BERIT | 949.746 | 2575.21 | 8512.93 | 8996.6 |
| Sambucana | SAMIT | 364.693 | 9757.3 | 19344.8 | 20650.5 |
| Biellese | BIEIT | 948.078 | 5529.52 | 2594.77 | 2293.93 |
| Delle Langhe | DLLIT | 102.929 | 3699.6 | 7498.41 | 7959.61 |
| Bundner Oberländer | BOSCH | 36.8177 | 16639.4 | 14918.6 | 13329.2 |
| Mirror | SMSCH | 59.1951 | 3790.18 | 4283.04 | 3509.91 |
| Black-Brown Mountain | SBSCH | 128.734 | 2905.58 | 4827.64 | 4768.56 |
| White Alpine | SWACH | 290.192 | 9535.72 | 11619.9 | 13271.5 |
| Valais Blacknose | VBSCH | 125.231 | 316.011 | 138.622 | 66.1004 |
| Valais Red | VRSCH | 50.2389 | 1322.48 | 1053.76 | 549.946 |
| Engadine Red | ERSCH | 367.941 | 3308.55 | 12251.4 | 13267.4 |
| Racka | RAKHU | 98.1575 | 2914.93 | 3713.56 | 3665.78 |
| Tsigaia | TSIRO | 1438.08 | 51943 | 63283.8 | 66209.4 |
| Cikta | CIKHU | 135.19 | 20392 | 40850.2 | 42534 |
| Kamieniec | KAMPL | 407.356 | 45498.2 | 55874.5 | 58283.6 |
| Kuchugur | KUHRU | 435.766 | 836.214 | 20575.6 | 22087.1 |
| Romanov | ROMRU | 236.617 | 3194.02 | 2984.38 | 2718.22 |
| Finnsheep | FINFI | 259.934 | 4374.91 | 2830.09 | 2564.76 |
| Merinolandschaf | MLSDE | 208.881 | 3866.45 | 9705.3 | 10454.4 |
| Black-Headed Mutton | BHMDE | 153.837 | 1112.84 | 5337.43 | 6252.85 |
| German Heath | GHHDE | 256.278 | 867.251 | 2435.92 | 1987.49 |
| Drenthe Horned Heath | DHTNL | 128.619 | 2095.3 | 6750.19 | 7284.72 |
| East-Friesian | EFRDE | 173.637 | 1286.23 | 1430.47 | 1305.77 |
| Texel | TXLNL | 201.353 | 2884.22 | 3318.2 | 3182.41 |
| Suffolk | SUFUK | 212.326 | 3864.67 | 4101.88 | 4575.84 |
| Scottish Blackface | SBFUK | 245.083 | 2084.59 | 5687.99 | 6198.91 |
| Soay | SOYUK | 87.6624 | 112.931 | 704.018 | 831.006 |
| Boreray | BORUK | 33.288 | 441.356 | 444.329 | 228.424 |
| Old Norwegian Spæl | NSONO | 254.907 | 12279.2 | 12082.3 | 8155.16 |
| Spæl-White | NSWNO | 237.51 | 6860.16 | 6603.32 | 6468.8 |
| Ile de France | IDFFR | 258.218 | 2973.94 | 2587.46 | 2833.82 |
| Milk-Lacaune | LALFR | 155.729 | 6797.27 | 10060 | 11749.8 |
| Meat-Lacaune | LAMFR | 209.578 | 5110.77 | 9223.16 | 9399.71 |
| Berrichon du Cher | BDCFR | 85.9581 | 894.864 | 698.754 | 615.756 |
| Merinos de Rambouillet | MDRFR | 728.607 | 2226.73 | 5403.4 | 5535.35 |
| Merinos dArles | MDAFR | 444.569 | 89800.8 | 94154 | 91216.7 |
| Latxa | LTXES | 276.002 | 3003.02 | 5747.29 | 6784.01 |
| Sasi-Ardi | SAAES | 804.675 | 2918.93 | 2122.67 | 3247.32 |
| Ripollesa | RIPES | 271.556 | 6162.68 | 16975.4 | 18295.3 |
| Xisqueta | XISES | 122786 | 55432.3 | 5759.22 | 5728.27 |
| Rasa Aragonesa | RAAES | 71982.9 | 43021.9 | 44996.8 | 54114.3 |
| Churra | CHUES | 217.23 | 24609.9 | 28554.6 | 33763.5 |
| Castellana | CASES | 92114.5 | 8741.07 | 8244.59 | 18676.1 |
| Ojalada | OJAES | 222.037 | 6327.32 | 8923.22 | 20162.5 |
| Segurena | SEGES | 1426.84 | 12488.8 | 92003 | 106546 |
| Merino Estremadura | MEDES | 221.463 | 4962.38 | 8263.03 | 9114.34 |

Effective population size *N_e_* values estimated by the GONE software at 5, 50, 85, and 100 generations ago for all 123 analysed sheep breeds and ovine populations.
