# Supplementary material for: Unravelling the genetic structure and genetic relationships of indigenous Greek sheep breeds using SNP markers
Source: Genet Sel Evol. 2026 Aug 3;58:50. doi: 10.1186/s12711-026-01074-5 (PMC13491747; doi:10.1186/s12711-026-01074-5)
Supplement: Supplementary file 7 [file 12711_2026_1074_MOESM1_ESM.docx]

| **Additional File 1 Table S1 Sample description** | | | | | | | | | | |
| --- | --- | --- | --- | --- | --- | --- | --- | --- | --- | --- |
| **Group** | **RGB code** | **Breed/Population** | **Code** | **N** | **Nd** | **N_al_** | **N_ad_** | **Breeding purposes** | **Breed Origin** | **Source of Genotype** |
| Outgroup | 0/0/0  (black) | Snow sheep | ONL-RU | 24 | 24 | 24 | 24 | Meat (hunting) | Siberia | [78] |
|  |  | Asian Mouflon | OGG-IR | 19 | 19 | 19 | 19 | Conservation purposes | Iran | [75] |
|  |  | Cyprus Mouflon | OGP-CY | 25 | 25 | 25 | 25 | Conservation purposes | Cyprus | This study |
|  |  | Sardinian Mouflon | OGM-IT | 22 | 17 | 17 | 17 | Conservation purposes | Sardinia | [75] |
|  |  | Corsican Mouflon | OGM-FR | 17 | 14 | 14 | 14 | Conservation purposes | Corsica | [75] |
| Minor Asia and North Africa | 255/0/0  (red) | Buubei | BUU-RU | 17 | 17 | 17 | 17 | Meat | Russia (Buryatia) | [77] |
|  |  | Tuvan | TUV-RU | 16 | 16 | 16 | 16 | Meat, wool | Russia (Tuvan) | [77] |
|  |  | Karakul (Romania) | KAR-KZ | 28 | 28 | 28 | 28 | Meat, milk, wool, pelts | Kazachstan | [77] |
|  |  | Zel | ZEL-IR | 24 | 24 | 24 | 24 | Meat, milk, wool | Iran (North) | [79] |
|  |  | Lori-Bakhtiari | LBA-IR | 24 | 24 | 24 | 24 | Meat, milk | Iran (West) | [79] |
|  |  | Moghani | MOG-IR | 34 | 33 | 33 | 33 | Meat, wool | Iran (North-West) | [43] |
|  |  | Afshari | AFS-IR | 37 | 33 | 33 | 33 | Meat | Iran (North-West) | [43] |
|  |  | Qezel | QEZ-TR | 35 | 35 | 35 | 35 | Meat, Milk | Turkey (Eastern) | [43] |
|  |  | Norduz | NDZ-TR | 20 | 20 | 20 | 20 | Meat, Milk | Turkey (Eastern) | [43] |
|  |  | Karakas | KRS-TR | 18 | 17 | 17 | 17 | Meat, milk, wool | Turkey (Eastern) | [43] |
|  |  | Lezgin | LEZ-RU | 15 | 15 | 15 | 15 | Meat, milk | Russia (Dagestan) | [77] |
|  |  | Karachaev | KRH-RU | 16 | 16 | 16 | 16 | Meat | Russia (Cherkessia) | [77] |
|  |  | Local Awassi | AWS-IL | 24 | 24 | 24 | 24 | Meat, milk, wool | Israel | [80] |
|  |  | Cyprus Fat-Tail | CFT-CY | 50 | 36 | 36 | 36 | Milk & Meat | Cyprus | This study & [43] |
|  |  | Ossimi | OSS-EG | 8 | 8 | 8 | 8 | Meat, milk, wool | Egypt | [80] |
|  |  | Egyptian Barki | BRK-EG | 13 | 13 | 13 | 13 | Meat, milk | Egypt | [80] |
|  |  | Sakiz | SKZ-TR | 22 | 16 | 16 | 16 | Meat, milk | Turkey (Western) | [43] |
| **Greece** | 0/0/255  (blue) | Rodos | ROD-GR | 4 | 4 | 0 | 0 | Milk & Meat | Greece | This study |
|  |  | Karpathos | KRP-GR | 16 | 13 | 13 | 7 | Milk & Meat | Greece | This study |
|  |  | Kasos | KSO-GR | 58 | 30 | 30 | 25 | Milk & Meat | Greece | This study |
|  |  | Sitia | SHT-GR | 19 | 13 | 13 | 7 | Milk & Meat | Greece | This study |
|  |  | Lasithi Plateu (Local) | PLS-GR | 11 | 11 | 11 | 9 | Milk & Meat | Greece | This study |
|  |  | Asterousia | AST-GR | 40 | 26 | 26 | 21 | Milk & Meat | Greece | This study |
|  |  | Anogia | ANG-GR | 25 | 13 | 13 | 12 | Milk & Meat | Greece | This study |
|  |  | Sfakia | SFK-GR | 35 | 31 | 31 | 21 | Milk & Meat | Greece | This study |
|  |  | Ikaria | IKR-GR | 5 | 4 | 0 | 0 | Milk & Meat | Greece | This study |
|  |  | Chios | HIO-GR | 70 | 46 | 46 | 9 | Milk & Meat | Greece | This study & [43] |
|  |  | Lesvos | LES-GR | 73 | 48 | 48 | 41 | Milk & Meat | Greece | This study & [39] |
|  |  | Skyros | SKY-GR | 14 | 12 | 12 | 9 | Milk & Meat | Greece | This study |
|  |  | Karystos | KRY-GR | 29 | 22 | 22 | 17 | Milk & Meat | Greece | This study |
|  |  | Kymi | KYM-GR | 15 | 15 | 15 | 9 | Milk & Meat | Greece | This study & [39] |
|  |  | Kalarrytiko | KLR-GR | 31 | 21 | 21 | 16 | Milk & Meat | Greece | This study |
|  |  | Kokovitiko | KOK-GR | 24 | 11 | 11 | 7 | Milk & Meat | Greece | This study |
|  |  | Argos | ARG-GR | 31 | 20 | 20 | 9 | Milk & Meat | Greece | This study |
|  |  | Vlahiko Peloponnese | VLP-GR | 7 | 7 | 7 | 7 | Milk & Meat | Greece | This study |
|  |  | Zakynthos | ZAK-GR | 14 | 12 | 12 | 7 | Milk & Meat | Greece | This study |
|  |  | Kefalonia | KEF-GR | 29 | 28 | 28 | 20 | Milk & Meat | Greece | This study |
|  |  | Arta | ART-GR | 14 | 14 | 14 | 11 | Milk & Meat | Greece | This study |
|  |  | Agrinio | AGR-GR | 20 | 11 | 11 | 3 | Milk & Meat | Greece | This study |
|  |  | Tsipoureika | TSP-GR | 3 | 3 | 0 | 0 | Milk & Meat | Greece | This study |
|  |  | Skopelos | SKP-GR | 31 | 17 | 17 | 9 | Milk & Meat | Greece | This study |
|  |  | Pelion | PEL-GR | 29 | 22 | 22 | 14 | Milk & Meat | Greece | This study |
|  |  | Vlahiko | VLH-GR | 25 | 25 | 25 | 18 | Milk & Meat | Greece | This study |
|  |  | Karagouniko | KRG-GR | 27 | 23 | 23 | 17 | Milk & Meat | Greece | This study |
|  |  | Thraki | THR-GR | 14 | 14 | 14 | 2 | Milk & Meat | Greece | This study |
|  |  | Serres | SER-GR | 14 | 11 | 11 | 5 | Milk & Meat | Greece | This study |
|  |  | Roumlouki | RML-GR | 4 | 4 | 0 | 0 | Milk & Meat | Greece | This study |
|  |  | Pelagonia | PLG-GR | 12 | 8 | 8 | 5 | Milk & Meat | Greece | This study |
|  |  | Sarakatsaniko | SRK-GR | 29 | 20 | 20 | 13 | Milk & Meat | Greece | This study |
|  |  | Katsika | KTS-GR | 10 | 10 | 10 | 6 | Milk & Meat | Greece | This study |
|  |  | Epirus Orino | BUT-GR | 10 | 10 | 10 | 5 | Milk & Meat | Greece | This study |
|  |  | Katafygio | KTF-GR | 8 | 7 | 7 | 5 | Milk & Meat | Greece | This study |
|  |  | Lefkimmi | LEU-GR | 19 | 10 | 10 | 6 | Milk & Meat | Greece | This study |
| Italy (South and Middle) | 0/255/0  (green) | Comisana | COM-IT | 24 | 24 | 24 | 24 | Milk (dairy), Meat and wool | Sicily | [43] |
|  |  | Pinzirita | PIN-IT | 24 | 24 | 24 | 24 | Milk, meat and wool | Sicily | [75] |
|  |  | Valle del Belice | VDB-IT | 24 | 24 | 24 | 24 | Milk | Sicily | [75] |
|  |  | Sardinian White | SAW-IT | 24 | 24 | 24 | 24 | Milk (cheese) | Sardinia | [75] |
|  |  | Sardinian Ancestral Black | SAB-IT | 20 | 20 | 20 | 20 | Milk | Sardinia | [43] |
|  |  | Leccese | LEC-IT | 24 | 22 | 22 | 22 | Milk, meat and wool | South | [43] |
|  |  | Altamurana | ALM-IT | 23 | 23 | 23 | 23 | Milk, meat and wool | South | [43] |
|  |  | Bagnolese | BAG-IT | 24 | 24 | 24 | 24 | Milk (cheese), meat | South | [75] |
|  |  | Laticauda | LAT-IT | 24 | 24 | 24 | 24 | Milk, meat | South | [75] |
|  |  | Gentile di Puglia | GEN-IT | 24 | 24 | 24 | 24 | Wool, milk, meat | South | [75] |
|  |  | Sopravissana | SOP-IT | 24 | 24 | 24 | 24 | Wool, milk, meat | South | [75] |
|  |  | Appenninica | APP-IT | 24 | 24 | 24 | 24 | Meat, (milk and wool) | Centre | [75] |
|  |  | Fabrianese | FAB-IT | 24 | 24 | 24 | 24 | Meat, milk | Centre | [75] |
|  |  | Massese | MAS-IT | 24 | 24 | 24 | 24 | Milk, meat | Centre | [75] |
| Balkans | 255/165/0  (orange) | Karakachanska | KRC-MK | 6 | 6 | 6 | 6 | Milk, meat | North Macedonia | [39] |
|  |  | Ovchepolean | OVP-MK | 10 | 10 | 10 | 10 | Milk, meat | North Macedonia | [39] |
|  |  | Balusha | BLS-XK | 30 | 25 | 25 | 25 | Milk, meat, wool | Kosovo | [76] |
|  |  | Ruda | RUD-AL | 16 | 16 | 16 | 16 | Wool | Albania | [39] |
|  |  | Pramenka (Sjenica) | SJE-BA | 16 | 16 | 16 | 16 | Milk, meat | Baosnia | This study |
|  |  | Pramenka (Privor) | PRI-BA | 16 | 15 | 15 | 15 | Milk, meat | Baosnia | This study |
| Alpe-Adria | 0/100/0 (dark green) | Rab | RAB-HR | 31 | 25 | 25 | 25 | Milk, meat | Croatia (island Rab) | This study |
|  |  | Istrian | IST-SI | 24 | 24 | 24 | 24 | Milk, meat | Croatia (Istria) | [75] |
|  |  | Alpagota | ALP-IT | 24 | 24 | 24 | 24 | Meat, milk, wool | Italy (North) | [75] |
|  |  | Bergamasca | BER-IT | 24 | 24 | 24 | 24 | Meat, milk | Italy (North) | [75] |
|  |  | Sambucana | SAM-IT | 24 | 24 | 24 | 24 | Meat, wool | Italy (North) | [75] |
|  |  | Biellese | BIE-IT | 24 | 24 | 24 | 24 | Meat, (wool) | Italy (North) | [75] |
|  |  | Delle Langhe | DLL-IT | 24 | 24 | 24 | 24 | Milk, meat, wool | Italy (North) | [75] |
|  |  | Bundner Oberländer | BOS-CH | 24 | 21 | 21 | 21 | Meat, (wool) | Switzerland | [43] |
|  |  | Mirror | SMS-CH | 24 | 20 | 20 | 20 | Meat | Switzerland | [43] |
|  |  | Black-Brown Mountain | SBS-CH | 24 | 23 | 23 | 23 | Wool | Switzerland | [43] |
|  |  | White Alpine | SWA-CH | 24 | 21 | 21 | 21 | Milk, meat, wool | Switzerland | [43] |
|  |  | Valais Blacknose | VBS-CH | 24 | 21 | 21 | 21 | Meat, wool | Switzerland | [43] |
|  |  | Valais Red | VRS-CH | 24 | 17 | 17 | 17 | Meat, wool | Switzerland | [43] |
|  |  | Engadine Red | ERS-CH | 24 | 21 | 21 | 21 | Meat | Switzerland | [43] |
| From Panonia to the North | 165/42/42  (brown) | Racka | RAK-HU | 16 | 15 | 15 | 15 | Milk, wool, meat | Hungary | This study & [39] |
|  |  | Tsigaia | TSI-RO | 27 | 26 | 26 | 26 | Meat, wool, milk | Hungary & Romania | [39] |
|  |  | Cikta | CIK-HU | 8 | 8 | 8 | 8 | Meat, wool | Hungary | [81] |
|  |  | Kamieniec | KAM-PL | 6 | 6 | 6 | 6 | Meat, wool | Poland | [39] |
|  |  | Kuchugur | KUH-RU | 16 | 15 | 15 | 15 | Meat, wool, milk | Russia | [77] |
|  |  | Romanov | ROM-RU | 32 | 32 | 32 | 32 | Meat, wool | Russia | [77] |
|  |  | Finnsheep | FIN-FI | 45 | 45 | 45 | 45 | Meat, wool, milk | Finland | [43] |
| From C-Europa to North-West | 143/0/255  (violet) | Merinolandschaf | MLS-DE | 45 | 44 | 44 | 44 | wool, meat | Germany | This study & [43] |
|  |  | Black-Headed Mutton | BHM-DE | 24 | 17 | 17 | 17 | Meat, wool | Germany | [43] |
|  |  | German Heath | GHH-DE | 7 | 7 | 7 | 7 | Meat, wool | Germany | [82] |
|  |  | Drenthe Horned Heath | DHT-NL | 5 | 5 | 5 | 5 | Meat, wool | Netherlands | [82] |
|  |  | East-Friesian | EFR-DE | 27 | 23 | 23 | 23 | Milk, meat, crossbreeding, wool | Germany | [43] |
|  |  | Texel | TXL-NL | 24 | 24 | 24 | 24 | Meat, wool | Netherlands | [83] |
|  |  | Suffolk | SUF-UK | 19 | 19 | 19 | 19 | Meat, wool | UK | [83] |
|  |  | Scottish Blackface | SBF-UK | 56 | 34 | 34 | 34 | Meat, wool | UK | [43] |
|  |  | Soay | SOY-UK | 46 | 45 | 45 | 45 | Wool, meat | UK | [43] |
|  |  | Boreray | BOR-UK | 17 | 14 | 14 | 14 | Wool, meat | UK | [43] |
|  |  | Old Norwegian Spæl | NSO-NO | 15 | 15 | 15 | 15 | Wool, meat | Norway | [43] |
|  |  | Spæl-White | NSW-NO | 32 | 29 | 29 | 29 | Meat, wool | Norway | [43] |
| From C-Europa to West | 128/128/128  (grey) | Ile de France | IDF-FR | 23 | 23 | 23 | 23 | Meat, crossbreeding,wool | France | [83] |
|  |  | Milk-Lacaune | LAL-FR | 39 | 39 | 39 | 39 | Milk, wool, crossbreeding | France | [43] |
|  |  | Meat-Lacaune | LAM-FR | 30 | 30 | 30 | 30 | Meat, crossbreeding, wool | France | [43] |
|  |  | Berrichon du Cher | BDC-FR | 19 | 19 | 19 | 19 | Meat, crossbreeding, wool | France | [83] |
|  |  | Merinos de Rambouillet | MDR-FR | 102 | 45 | 45 | 45 | Wool, meat, crossbreeding | France | [43] |
|  |  | Merinos dArles | MDA-FR | 18 | 18 | 18 | 18 | wool, meat | France | [83] |
|  |  | Latxa | LTX-ES | 19 | 19 | 19 | 19 | Milk (cheese), wool | Basque | [84] |
|  |  | Sasi-Ardi | SAA-ES | 24 | 24 | 24 | 24 | Meat, milk (cheese), wool | Basque | [39] |
|  |  | Ripollesa | RIP-ES | 21 | 21 | 21 | 21 | Meat, wool | Northeast | [74] |
|  |  | Xisqueta | XIS-ES | 24 | 24 | 24 | 24 | Meat, wool | Northeast | [74] |
|  |  | Rasa Aragonesa | RAA-ES | 22 | 20 | 20 | 20 | Meat, wool | Northeast | [43] |
|  |  | Churra | CHU-ES | 120 | 45 | 45 | 45 | Meat, wool | Spain | [43] |
|  |  | Castellana | CAS-ES | 23 | 22 | 22 | 22 | Meat, wool | Centre | [43] |
|  |  | Ojalada | OJA-ES | 24 | 24 | 24 | 24 | Meat, wool | Centre | [43] |
|  |  | Segurena | SEG-ES | 12 | 12 | 12 | 12 | Meat, wool | South | [74] |
|  |  | Merino Estremadura | MED-ES | 13 | 13 | 13 | 13 | Wool, meat | South | [82] |

Group allocation, RGB (color) code assigned to the pre-defined groups within this paper, breed names, breed code, number of sampled and genotyped individuals (*N*), number of animals after exclusion of highly related animals and outlier (*N_d_*), number of genotyped and unrelated individuals used to estimate the diversity parameters (*N_al_*), number of unrelated animals used for the admixture analysis (*N_ad_*), current breeding purposes as well as sporadic or recent past breeding purposes, breed origin, and source of the samples or genotypes used in this study or from previous studies [39,43,74-84]

| **Additional File 1 Table S2 Phenotypic traits of each Greek sheep breed/population** | | | | | | | | | |
| --- | --- | --- | --- | --- | --- | --- | --- | --- | --- |
| **Breeds/Population** | **Code** | N_m_ | | **Phenotypic Traits** | | | | | |
|  |  |  |  | **Body weight (kg)** | | **Withers height (cm)** | | **Body length (cm)** | |
|  |  | *Female* | *Male* | *Female* | *Male* | *Female* | *Male* | *Female* | *Male* |
| Agrinio | AGRGR | 16 | 4 | 48.9 ± 3.0 | 70.5 ± 3.3 | 72.2 ± 2.9 | 77.8 ± 2.4 | 73.4 ± 4.3 | 83.0 ± 6.2 |
| Anogia | ANGGR | 7 | 8 | 21.0 ± 2.5 | 32.3 ± 3.3 | 62.3 ± 3.1 | 63.2 ± 18.3 | 63.5 ± 3.4 | 68.8 ± 5.3 |
| Argos | ARGGR | 2 | - | 46.5 ± 4.9 | - | 69.0 ± 1.4 | - | 78.5 ± 2.1 | - |
| Arta | ARTGR | 14 | 14 | 62.6 ± 8.0 | - | 73.3 ± 3.5 | - | 82.4 ± 7.7 | - |
| Asterousia | ASTGR | 25 | 8 | 29.0 ± 5.8 | 44.7 ± 6.5 | 60.6 ± 4.8 | 66.5 ± 11.4 | 63.3 ± 5.4 | 72.8 ± 7.4 |
| Chios | HIOGR | 4 | 4 | 67.5 ± 3.3 | 86.3 ± 7.5 | 79.5 ± 3.4 | 83.0 ± 6.2 | 83.5 ± 4.9 | 88.5 ± 8.4 |
| Epirus Orino | BUTGR | 4 | 2 | 37.8 ± 4.4 | 52.5 ± 3.5 | 63.5 ± 2.5 | 65.5 ± 2.1 | 67.5 ± 2.4 | 71.5 ± 2.1 |
| Ikaria | IKRGR | 1 | - | 40 | - | 61 | - | 62 | - |
| Kalarrytiko | KLRGR | 16 | 4 | 41.4 ± 4.7 | 59.5 ± 13.7 | 65.2 ± 4.8 | 71.0 ± 4.3 | 70.1 ± 5.6 | 78.5 ± 5.0 |
| Karagouniko | KRGGR | 21 | 6 | 74.1 ± 5.4 | 95.8 ± 8.0 | 79.4 ± 3.4 | 91.3 ± 4.9 | 83.5 ± 4.3 | 94.4 ± 3.4 |
| Karpathos | KRPGR | - | - | - | - | - | - | - | - |
| Karystos | KRYGR | 15 | 1 | 39.9 ± 3.4 | 47 | 65.2 ± 4.2 | 67 | 76.4 ± 6.1 | 64 |
| Kasos | KSOGR | 38 | 8 | 24.0 ± 3.7 | 42.5 ± 9.3 | 58.1 ± 4.9 | 69.4 ± 4.5 | 59.9 ± 4.1 | 71.1 ± 5.1 |
| Katafygio | KTFGR | 6 | 2 | 36.5 ± 5.9 | 40.0 ± 28.3 | 65.0 ± 5.2 | 66.0 ± 9.9 | 69.5 ± 3.5 | 67.0 ± 14.1 |
| Katsika | KTSGR | 5 | 1 | 56.7 ± 6.1 | 65 | 73.0 ± 4.1 | 73 | 81.2 ± 5.6 | 86 |
| Kefalonia | KEFGR | 18 | 11 | 41.2 ± 2.4 | 54.7 ± 8.4 | 66.8 ± 3.2 | 72.6 ± 2.7 | 69.8 ± 2.8 | 76.8 ± 4.6 |
| Kokovitiko | KOKGR | 16 | 5 | 37.0 ± 4.4 | 48.2 ± 5.8 | 62.9 ± 3.9 | 67.0 ± 2.5 | 67.6 ± 4.4 | 71.8 ± 5.00 |
| Kymi | KYMGR | 7 | 2 | 48.4 ± 1.1 | 63.5 ± 4.9 | 76.2 ± 2.3 | 75.8 ± 3.2 | 82.0 ± 4.2 | 84.5 ± 4.9 |
| Lefkimmi | LEUGR | 9 | 5 | 38.6 ± 5.8 | 68.00 ± 9.8 | 71.2 ± 3.9 | 81.0 ± 4.4 | 73.7 ± 3.6 | 81.8 ± 3.0 |
| Lesvos | LESGR | - | - | - | - | - | - | - | - |
| Pelagonia | PLGGR | 7 | 3 | 43.8 ± 4.8 | 51.7 ± 11.6 | 72.0 ± 1.6 | 79.3 ± 3.2 | 74.3 ± 7.5 | 85.0 ± 2.7 |
| Pelion | PELGR | 2 | 3 | 60.0 ± 7.1 | 73.3 ± 2.9 | 64.5 ± 3.5 | 72.5 ± 3.5 | 68.0 ± 7.1 | 81.5 ± 0.7 |
| Plateu Local Lasithi | PLSGR | 4 | 3 | 24.8 ± 3.3 | 37.7 ± 2.5 | 61.8 ± 2.4 | 70.0 ± 2.0 | 62.5 ± 1.9 | 66.7 ± 4.7 |
| Rodos | RODGR | 2 | 2 | 43.5 ± 2.1 | 55.0 ± 7.1 | 65.5 ± 2.1 | 78 | 71.5 ± 2.1 | 73.0 ± 4.2 |
| Roumlouki | RMLGR | - | - | - | - | - | - | - | - |
| Sarakatsaniko | SRKGR | 15 | 5 | 33.5 ± 4.1 | 45.2 ± 3.3 | 60.4 ± 5.2 | 66.4 ± 7.6 | 64.1 ± 7.2 | 66.8 ± 8.9 |
| Serres | SERGR | 11 | 3 | 61.4 ± 23.0 | 105.0 ± 49.5 | 75.3 ± 4.0 | 81.0 ± 7.6 | 79.2 ± 6.1 | 83.7 ± 5.5 |
| Sfakia | SFKGR | 17 | 4 | 38.0 ± 4.2 | 63.0 ± 12.4 | 62.7 ± 2.4 | 73.0 ± 3.7 | 65.4 ± 5.5 | 74.3 ± 1.7 |
| Sitia | SHTGR | 4 | - | 40.0 ± 7.1 | - | 65.3 ± 4.3 | - | 67.5 ± 5.3 | - |
| Skopelos | SKPGR | 15 | 5 | 52.5 ± 9.7 | 81.60 ± 6.4 | 67.20 ± 4.6 | 78.8 ± 3.1 | 72.0 ± 3.3 | 85.4 ± 4.00 |
| Skyros | SKYGR | 7 | 4 | 39.0 ± 3.3 | 59.5 ± 4.2 | 63.3 ± 3.2 | 76.5 ± 4.4 | 67.8 ± 4.2 | 78.5 ± 7.7 |
| Thraki | THRGR | - | - | - | - | - | - | - | - |
| Tsipoureika | TSPGR | 2 | 1 | 33.5 ± 2.1 | 50 | 65.5 ± 0.7 | 78 | 69.0 ± 1.4 | 78 |
| Vlahiko | VLHGR | 19 | 6 | 33.5 ± 5.6 | 42.5 ± 2.3 | 64.2 ± 3.3 | 68.0 ± 4.2 | 65.4 ± 4.0 | 68.67 ± 2.2 |
| Vlahiko Peloponnese | VLPGR | 8 | - | 45.4 ± 4.2 | - | 68.0 ± 1.8 | - | 73.3 ± 4.2 | - |
| Zakynthos | ZAKGR | - | - | - | - | - | - | - | - |

Body weight (in kg), withers height (in cm) and body length (in cm) per gender (Female and Male). *N_m_*: number of animals that measurements were taken per gender.

| **Additional File 1 Table S3 Sampling information of the Greek sheep populations included in the study** | | | | | | | | |  |
| --- | --- | --- | --- | --- | --- | --- | --- | --- | --- |
| **Breed** | **Code** | **Date** | **Flock No.** | **No. samples per flock** | **N (same as Table 1)** | **Location** | | | |
|  |  |  |  |  |  | **Area** | **Latitude** | **Longitude** | |
| Agrinio | **AGRGR** | 30/11/2021 | 1 | 11 | 20 | Neapoli / Platitsi / Aetolia-Acarnania / Central Greece | 38.689309 | 21.362139 | |
|  |  | 30/11/2021 | 2 | 9 |  | Panaitolio / Aetolia-Acarnania / Central Greece | 38.578734 | 21.413361 | |
| Anogia | **ANGGR** | 2017 | 1 | 7 | 25 | Klima / Tympaki / Heraklion / Crete | 35.286542 | 24.907663 | |
|  |  | 2017 | 2 | 6 |  | Rizikas / Rethymno / Crete | 35.286542 | 24.907663 | |
|  |  | 4/4/2022 | 3 | 3 |  | Kounou / Anogia / Rethymno / Crete | 35.286542 | 24.907663 | |
|  |  | 4/4/2022 | 4 | 4 |  | Stefana/ Anogia/ Crete | 35.253757 | 24.848500 | |
|  |  | 4/4/2022 | 5 | 5 |  | Panampelia / Anogia / Rethymno / Crete | 35.272836 | 24.891156 | |
| Argos | **ARGGR** | 2018 | 1 | 6 | 31 | Orchomenos / Boeotia / Central Greece | 38.496422 | 22.984722 | |
|  |  | 27/9/2018 | 2 | 14 |  | Myrtia / Elis / Peloponnese | 37.714125 | 21.35399 | |
|  |  | 27/9/2018 & 2/5/2022 | 3 | 11 |  | Vitineika / Elis / Peloponnese | 37.695075 | 21.378095 | |
| Arta | **ARTGR** | 7/7/2021 | 1 | 7 | 14 | Ano Vasiliki / Messolonghi / Western Greece | 38.394766 | 21.600342 | |
|  |  | 18/5/2022 | 2 | 4 |  | Filothei / Arta / Epirus | 39.144335 | 20.905384 | |
|  |  | 9/4/2024 | 3 | 3 |  | Agia Paraskevi / Arta / Epirus | 39.077308 | 20.979914 | |
| Asterousia | **ASTGR** | 2017 | 1 | 9 | 40 | Fournofarago / Heraklion / Crete | 35.068897 | 24.779627 | |
|  |  | 29/6/2021 | 2 | 10 |  | Tympaki / Heraklion / Crete | 35.068897 | 24.779627 | |
|  |  | 30/6/2021 | 3 | 8 |  | Agarathos / Heraklion / Crete | 35.224714 | 25.265909 | |
|  |  | 31/5/2023  & 6/3/2024 | 4 | 13 |  | Kofinas / Heraklion / Crete | 34.9743479/  34.963754/  34.963389 | 25.0824764/  25.087853/  25.088028 | |
| Chios | **HIOGR** | 1/4/2018 | 1 | 9 | 70 | Paralimni / Giannitsa / Pella / Central Macedonia | 40.743342 | 22.455246 | |
|  |  | 23/9/2019 | 2 | 15 |  | Chios Island / North Aegean | 38.3722 | 26.07793 | |
|  |  | 30/5/2022 | 3 | 14 |  | Agia Paraskevi / Karies / Chios / North Aegean | 38.37220 | 26.07793 | |
|  |  | 2021 | 4 | 10 |  | Athens / Attica | 37.98527 | 23.706632 | |
|  |  | - | Previous study data | 22 |  | Genotypes from [43] | - | - | |
| Cyprus fat-tailed | **CFTCY** | 2018 | 1 | 10 | 50 | Eurihou / Cyprus | 35.01665 | 32.90553 | |
|  |  | 2018 | 2 | 10 |  | Kakopetria / Cyprus | 34.97937 | 32.91873 | |
|  |  | - | Previous study data | 30 |  | Genotypes from [43] |  |  | |
| Epirus Orino | **BUTGR** | 29/8/2021 | 1 | 10 | 10 | Perdika / Thesprotia / Epirus | 39.37558 | 20.307166 | |
| Ikaria | **IKRGR** | 17/9/2019 | 1 | 5 | 5 | Ikaria Island / North Aegean | 37.607835 | 26.167359 | |
| Kalarrytiko | **KLRGR** | 26/5/2021 | 1 | 10 | 31 | Damasi / Tyrnavos / Larissa / Thessaly | 39.709209 | 22.184688 | |
|  |  | 20/11/2021 | 2 | 4 |  | Vlochos / Farkadona / Karditsa / Thessaly / Greece | 39.55042 | 22.09562 | |
|  |  | 18/5/2022 | 3 | 7 |  | Katsika / Metsovo / Ioannina / Epirus | 39.521235 | 20.26276 | |
|  |  | 8/4/2024 | 4 | 10 |  | Enosi Georgikou Sineterismou Ioanninon/ Epirus | 39.634085 | 20.879993 | |
| Karagouniko | **KRGGR** | 9/11/2022 | 1 | 8 | 27 | Rousso / Karditsa / Thessaly | 39.312244 | 21.905284 | |
|  |  | 9/11/2022 | 2 | 11 |  | Marko / Karditsa / Thessaly | 39.428058 | 22.03585 | |
|  |  | 9/11/2022 | 3 | 8 |  | Karditsa / Thessaly | 39.34565 | 21.90332 | |
| Karpathos | **KRPGR** | 23/5/2018 | 1 | 1 | 16 | Othos / Karpathos Island / South Aegean | 35.50396 | 27.183032 | |
|  |  | 23/5/2018 | 2 | 4 |  | Lastos / Karpathos Island / South Aegean | 35.575641 | 27.140553 | |
|  |  | 23/5/2018 | 3 | 2 |  | Olympos / Karpathos Island / South Aegean | 35.73939 | 27.176548 | |
|  |  | 23/5/2018 | 4 | 2 |  | Pyles Souri / Karpathos Island / South Aegean | 35.534441 | 27.131745 | |
|  |  | 23/5/2018 | 5 | 7 |  | Menetes / Karpathos Island / South Aegean | 35.45025 | 27.114742 | |
| Karystos | **KRYGR** | 1/4/2018 | 1 | 8 | 29 | Prinia / Kafodoro / Euboea / Central Greece | 38.138425 | 24.575945 | |
|  |  | 1/4/2018 | 2 | 5 |  | Likorema / Karystos / Euboea / Central Greece | 38.017961 | 24.333729 | |
|  |  | 15/5/2023 | 3 | 5 |  | Tsefi–Barouno / Antia / Karystos / Euboea / Central Greece | 38.057881 | 24.579619 | |
|  |  | 15/5/2023 | 4 | 6 |  | Antia / Karystos / Euboea / Central Greece | 38.059414 | 24.551475 | |
|  |  | 15/5/2023 | 5 | 5 |  | Komito / Karystos / Euboea / Central Greece | 38.0715412 | 24.5450348 | |
| Kasos | **KSOGR** | 22/5/2018 | 1 | 12 | 58 | Skafi / Kasos Island / South Aegean | 35.409685 | 26.976413 | |
|  |  | 27/3/2022 | 2 | 11 |  | Maritsa / Kasos Island / South Aegean | 35.418105 | 26.957341 | |
|  |  | 28/3/2022 | 3 | 6 |  | Ammoes / Kasos Island / South Aegean | 35.400973 | 26.953177 | |
|  |  | 28/3/2022 | 4 | 7 |  | Agia Kyriaki / Kasos Island / South Aegean | 35.405924 | 26.947108 | |
|  |  | 28/3/2022 | 5 | 2 |  | Schoinias / Kasos Island / South Aegean | 35.379156 | 26.89181 | |
|  |  | 29/3/2022 | 6 | 6 |  | Fantasi / Kasos Island / South Aegean | 35.384643 | 26.933839 | |
|  |  | 29/3/2022 | 7 | 10 |  | Skafi / Kasos Island / South Aegean | 35.405593 | 26.97207 | |
|  |  | 30/3/2022 | 8 | 4 |  | Agia Kyriaki / Kasos Island / South Aegean | 35.407509 | 26.949008 | |
| Katafygio | **KTFGR** | 17/5/2022 | 1 | 8 | 8 | Igoumenitsa / Thesprotia / Epirus | 39.50615 | 20.265534 | |
| Katsika | **KTSGR** | 7/6/2021 & 8/4/2024 | 1 | 10 | 10 | Gotista / Lakmos / Ioannina / Epirus | 39.687098/ 39.694136 | 21.12655/  20.999619 | |
| Kefalonia | **KEFGR** | 19/9/2022 | 1 | 12 | 29 | Kalon Oros / Agia Efimia / Kefalonia / Ionian Islands | 38.310118 | 20.600708 | |
|  |  | 20/9/2022 | 2 | 8 |  | Themata / Agios Dinatis / Kefalonia / Ionian Islands | 38.282877 | 20.575427 | |
|  |  | 22/9/2022 | 3 | 2 |  | Perachori / Ithaca / Ionian Islands | 38.352009 | 20.716931 | |
|  |  | 22/9/2022 | 4 | 7 |  | Vathy / Ithaca / Ionian Islands | 38.358066 | 20.728713 | |
| Kokovitiko | **KOKGR** | 2/4/2022 | 1 | 10 | 24 | Amaliada / Elis / Western Greece | 37.796792 | 21.350758 | |
|  |  | 2/4/2022 | 2 | 10 |  | Platanos / Ancient Olympia / Elis / Western Greece | 37.66402 | 21.61587 | |
|  |  | 3/4/2024 | 3 | 4 |  | Kastria/ Kalavrita | 37.948872 | 22.137251 | |
| Kymi | **KYMGR** | 13/5/2022 | 1 | 9 | 15 | Mentouli / Kymi / Euboea / Central Greece | 38.6040848 | 24.08630194 | |
|  |  | - | Previous study data | 6 |  | Genotypes from [39] | - | - | |
| Lefkimmi | **LEUGR** | 1/9/2020 | 1 | 5 | 19 | Xanthates / Corfu / Ionian Islands | 39.7648 | 19.762312 | |
|  |  | 1/9/2020 | 2 | 3 |  | Perivoli / Corfu / Ionian Islands | 39.419961 | 20.006024 | |
|  |  | 30/8/2021 | 3 | 4 |  | Vitalades (Marmaro) / Corfu / Ionian Islands | 39.419358 | 20.035608 | |
|  |  | 30/8/2021 | 4 | 2 |  | Perivoli / Corfu / Ionian Islands | 39.41861 | 20.009166 | |
|  |  | 30/8/2021 | 5 | 3 |  | Koryssia / Corfu / Ionian Islands | 39.453586 | 19.904516 | |
|  |  | 30/8/2021 | 6 | 3 |  | Platonas / Corfu / Ionian Islands | 39.76469 | 19.762321 | |
| Lesvos | **LESGR** | 19/9/2019 | 1 | 10 | 73 | Agia Paraskevi /Lesvos Island / North Aegean | 39.243578 | 26.317492 | |
|  |  | 19/9/2019 | 2 | 10 |  | Hidiron /Lesvos Island / North Aegean | 39.200436 | 26.030297 | |
|  |  | 19/9/2019 | 3 | 12 |  | Skalohori /Lesvos Island / North Aegean | 39.267312 | 26.049096 | |
|  |  | 19/9/2019 | 4 | 12 |  | Agra/Lesvos Island / North Aegean | 39.160945 | 26.066491 | |
|  |  | 19/9/2019 | 5 | 12 |  | Mesotopos /Lesvos Island / North Aegean | 39.1237450 | 26.014027 | |
|  |  | 19/9/2019 | 6 | 11 |  | Pelopi /Lesvos Island / North Aegean | 39.321546 | 26.277802 | |
|  |  | - | Previous study data | 6 |  | Genotypes from [39] | - | - | |
| Pelagonia | **PLGGR** | 2018 | 1 | 5 | 12 | Paralimni / Giannitsa / Pella / Central Macedonia | 40.743342 | 22.455246 | |
|  |  | 25/5/2021 | 2 | 7 |  | Siatista / Kozani / Western Macedonia | 40.269948 | 21.542714 | |
| Pelion | **PELGR** | 1/4/2018 | 1 | 7 | 29 | Milies / Magnesia / Thessaly | 39,317364 | 23,143152 | |
|  |  | 1/4/2018 | 2 | 7 |  | Milies / Magnesia / Thessaly | 39,32666 | 23,140017 | |
|  |  | 1/12/2019 | 3 | 10 |  | Ano Gazea / Magnesia / Thessaly | 39.318317 | 23.091328 | |
|  |  | 10/11/2022 | 4 | 5 |  | Velestino / Magnesia / Thessaly | 39.374421 | 22.735012 | |
| Plateu Local Lasithi | **PLSGR** | 12/6/2022 | 1 | 11 | 11 | Lasithi Plateau / Pinakiano / Lasithi / Crete | 35.1958 | 25.4493 | |
| Rodos | **RODGR** | 29/6/2023 | 1 | 4 | 4 | Maritsa/Rodos | 36.366397 | 28.113514 | |
| Roumlouki | **RMLGR** | 1/4/2018 | 1 | 4 | 4 | Kria Vrisi / Pella / Central Macedonia | 40.697862 | 22.297644 | |
| Sarakatsaniko | **SRKGR** | 25/5/2021 | 1 | 10 | 29 | Siatista / Kozani / Western Macedonia | 40.271955 | 21.528679 | |
|  |  | 12/1/2021 | 2 | 10 |  | Kipseli / Arta / Epirus | 39.348516 | 21.077676 | |
|  |  | 21/5/2024 | 3 | 9 |  | Makrychori / Larissa / Thessaly | 39.763344 | 22.495376 | |
| Serres | **SERGR** | 2/2/2023 | 1 | 9 | 14 | Vamvakia / Serres / Central Macedonia | 41.0861 | 23.3451 | |
|  |  | 2/2/2023 | 2 | 5 |  | Lygaria / Serres / Central Macedonia | 40.9811 | 23.4368 | |
| Sfakia | **SFKGR** | 2017 | 1 | 8 | 35 | Imbros / Sfakia / Chania / Crete | 35.234007 | 24.08932 | |
|  |  | 2017 | 2 | 6 |  | Chora/ Sfakia/ Chania/ Crete | 35.201193 | 24.136566 | |
|  |  | 28/6/2021 | 3 | 8 |  | Anopoli / Sfakia / Chania / Crete | 35.234007 | 24.08932 | |
|  |  | 28/6/2021 | 4 | 9 |  | Anopoli / Sfakia / Chania / Crete | 35.216596 | 24.09323 | |
|  |  | 7/3/2024 | 5 | 2 |  | Aradena / Chania / Crete | 35.223846 | 24.043514 | |
|  |  | 7/3/2024 | 6 | 2 |  | Skaloti / Chania / Crete | 35.203870 | 24.264114 | |
| Sitia | **SHTGR** | 22/4/2018 | 1 | 9 | 19 | Dafni / Lasithi / Crete | 35.107238 | 25.994876 | |
|  |  | 22/4/2018 | 2 | 7 |  | Paleokastro / Lasithi / Crete | 35.193297 | 26.219708 | |
|  |  | 29/5/2023 | 3 | 3 |  | Halasas/ Dafni/ Lasithi/ Crete | 35.111395 | 25.997806 | |
| Skopelos | **SKPGR** | 7/11/2022 | 1 | 10 | 31 | Agios Georgios / Velestino / Magnesia / Thessaly | 39.368008 | 22.783889 | |
|  |  | 10/11/2022 | 2 | 10 |  | Dimini / Volos / Magnesia / Thessaly | 39.35797 | 22.90427 | |
|  |  | 21/5/2024 | 3 | 11 |  | Stefanovikio / Volos / Magnesia / Thessaly | 39.436325 | 22.805365 | |
| Skyros | **SKYGR** | 1/4/2018 | 1 | 5 | 14 | Agia Paraskevi / Skyros Island / Central Greece | 38.933126 | 24.515608 | |
|  |  | 22/6/2021 | 2 | 10 |  | Polyhri / Skyros Island / Central Greece | 38.958089 | 24.500325 | |
| Thraki | **THRGR** | 18/4/2018 | 1 | 3 | 14 | Kaloticho / Rhodope / Thrace | 41.288729 | 25.184183 | |
|  |  | 18/4/2018 | 2 | 11 |  | Poa / Rhodope / Thrace | 41.214293 | 25.259436 | |
| Tsipoureika | **TSPGR** | 24/5/2021 | 1 | 3 | 3 | Karakasi / Fragista / Evrytania / Central Greece | 38.965736 | 21.568345 | |
| Vlahiko | **VLHGR** | 6/2/2023 | 1 | 18 | 25 | Ampelonas & Koutsohero/ Larissa / Thessaly | 39.829053/ 39.66023 | 22.409445/  22.22386 | |
|  |  | 6/2/2023 | 2 | 7 |  | Vlahogianni / Tyrnavos / Larissa / Thessaly | 39.750368 | 22.056275 | |
| Vlahiko Peloponnese | **VLPGR** | 2/3/2022 | 1 | 7 | 7 | Skepasto / Kalavryta / Achaea / Peloponnese | 38.060158 | 22.071884 | |
| Zakynthos | **ZAKGR** | 1/4/2018 | 1 | 4 | 14 | Agios Leon / Zakynthos Island / Ionian Islands | 37.76656 | 20.719807 | |
|  |  | 1/4/2018 | 2 | 10 |  | Gaitani / Zakynthos Island / Ionian Islands | 37.783425 | 20.877426 | |

Sampling dates, number of flocks, samples per flock, total number of sampled and genotyped individuals (N), sampling areas, and geographic coordinates for each Greek sheep breed/population and the Cyprus fat-tailed breed.
