# Supplementary material for: Unravelling the genetic structure and genetic relationships of indigenous Greek sheep breeds using SNP markers
Source: Genet Sel Evol. 2026 Aug 3;58:50. doi: 10.1186/s12711-026-01074-5 (PMC13491747; doi:10.1186/s12711-026-01074-5)
Supplement: Supplementary file 8 [file 12711_2026_1074_MOESM2_ESM.docx]

**Additional File 2 Table S4 Demographic and conservation status of the 36 Greek sheep breeds**

| **#** | **Breed** | **Breed Code** | **Recent population figures** | **Herdbook** | **Active breeding program** | **In situ conservation program** |
| --- | --- | --- | --- | --- | --- | --- |
| 1 | AGRINIO | AGRGR | 900 | YES | YES | YES |
| 2 | ANOGIA | ANGGR | - | YES | NO | YES |
| 3 | ARGOS | ARGGR | 26 (2021) | - | NO | NO |
| 4 | BOUTSIKO (EPIRUS ORINO) | BUTGR | 4500 | NO | YES | YES |
| 5 | CHIOS | HIOGR | 12097 | YES | YES | NO |
| 6 | FLORINA (PELAGONIAS) | PLGGR | 2298 | YES | YES | YES |
| 7 | ARTA | ARTGR | 45000 | YES | YES | NO |
| 8 | KALARRYTIKO | KLRGR | 8282 | YES | YES | YES |
| 9 | KARAGOUNIKO | KRGGR | 3936 | YES | YES | NO |
| 10 | KARYSTOS | KRYGR | 37500 | NO | NO | NO |
| 11 | KATAFYGIO | KTFGR | EXTINCT | - | - | - |
| 12 | KATSIKA | KTSGR | 1650 | YES | YES | YES |
| 13 | KEFALLINIA | KEFGR | 32500 | NO | NO | NO |
| 14 | KYMI | KYMGR | 900 | - | NO | YES |
| 15 | LESVOS | LESGR | 260000 | YES | YES | NO |
| 16 | ROUMLOUKI | RMLGR | 598 | NO | NO | YES |
| 17 | SARAKATSANIKO | SRKGR | 12621 | YES | YES | YES |
| 18 | SERRES | SERGR | 4779 | YES | YES | YES |
| 19 | SFAKIA | SFKGR | 68500 | YES | NO | NO |
| 20 | SITIA | SHTGR | - | - | NO | - |
| 21 | SKOPELOS | SKPGR | 2421 | YES | YES | YES |
| 22 | PELION | PELGR | 2973 | YES | YES | YES |
| 23 | THRAKI | THRGR | 7500 | - | NO | YES |
| 24 | VLAHIKO | VLHGR | - | NO | NO | NO |
| 25 | ZAKYNTHOS | ZAKGR | 1000 | - | NO | YES |
| 26 | IKARIA | IKRGR | 50 | NO | NO | YES |
| 27 | KOKOVITIKO | KOKGR | 200 | NO | NO | NO |
| 28 | LEFKIMMI | LEUGR | 50 | NO | NO | YES |
| 29 | ASTEROUSIA | ASTGR | 3000-4000 | NO | NO | NO |
| 30 | SKYROS | SKYGR | 2000 | NO | NO | NO |
| 31 | RODOS | RODGR | 100 | NO | NO | NO |
| 32 | KARPATHOS | KRPGR | 300 | NO | NO | NO |
| 33 | KASOS | KSOGR | 3000 | NO | NO | NO |
| 34 | LASITHI PLATEU (LOCAL) | PLSGR | 400 | NO | NO | NO |
| 35 | VLAHIKO PELOPONNESE | VLPGR | 400 | NO | NO | NO |
| 36 | TSIPOUREIKA | TSPGR | 600 | NO | NO | NO |

Population figures and information on breeding / conservation program correspond to 2023 data from the FAO Domestic Animal Diversity Information System (DAD-IS [93]). Breeds with no updated data are considered with missing or unavailable data in the registry, denoted by a dash (-). For local populations lacking official registry data, the population figures reflect the teams' expert assessments and field observations.

Note on Official Status: Following the initial data compilation for this study, the Ikaria and Lefkimmi breeds/ populations were formally granted officially recognized status by the Greek Ministry of Rural Development and Food, as published in the National Government Gazette (Ref. No. 56307/27.02.2025) [113].
