# Supplementary material for: Unravelling the genetic structure and genetic relationships of indigenous Greek sheep breeds using SNP markers
Source: Genet Sel Evol. 2026 Aug 3;58:50. doi: 10.1186/s12711-026-01074-5 (PMC13491747; doi:10.1186/s12711-026-01074-5)
Supplement: Supplementary file 9 [file 12711_2026_1074_MOESM3_ESM.docx]

| **Additional File 3 Table S5 Descriptive statistics and significance testing of genetic diversity parameters across the eight predefined geographical sheep groups** | | | | | | |
| --- | --- | --- | --- | --- | --- | --- |
| **Group** | **Statistic** | ***H_o_*** | ***H_e_*** | ***F*** | ***A_R_*** | ***Ne_5_*** |
| **SOUTHWEST ASIA & NORTH AFRICA** | Mean ± SD | 0.771 ± 0.028^a^ | 0.747 ± 0.038^a^ | 0.092 ± 0.032^b^ | 4.828 ± 0.450^b^ | 297.06 ± 290.37 |
|  | Median [95% CI] | 0.773 [0.757, 0.786] | 0.756 [0.728, 0.767] | 0.100 [0.076, 0.109] | 5.060 [4.782, 4.874] | 192.00 [147.76, 446.35] |
| **GREECE** | Mean ± SD | 0.750 ± 0.033^ab^ | 0.744 ± 0.031^ab^ | 0.093 ± 0.039^b^ | 4.861 ± 0.307^b^ | 210.22 ± 194.22 |
|  | Median [95% CI] | 0.758 [0.738, 0.762] | 0.751 [0.733, 0.755] | 0.085 [0.079, 0.107] | 4.960 [4.836, 4.886] | 134.50 [140.19, 280.24] |
| **ITALY** | Mean ± SD | 0.764 ± 0.021^a^ | 0.755 ± 0.029^a^ | 0.079 ± 0.027^b^ | 4.838 ± 0.330^b^ | 177.21 ± 93.97 |
|  | Median [95% CI] | 0.769 [0.752, 0.776] | 0.754 [0.738, 0.772] | 0.075 [0.063, 0.094] | 4.850 [4.802, 4.874] | 168.50 [122.96, 231.47] |
| **BALKANS** | Mean ± SD | 0.755 ± 0.056^ab^ | 0.736 ± 0.088^ab^ | 0.092 ± 0.076^b^ | 4.996 ± 0.534^a^ | 176.00 ± 93.55 |
|  | Median [95% CI] | 0.773 [0.697, 0.814] | 0.772 [0.644, 0.829] | 0.070 [0.012, 0.172] | 5.170 [4.883, 5.109] | 185.50 [77.82, 274.18] |
| **ALPE-ADRIA** | Mean ± SD | 0.750 ± 0.038^ab^ | 0.737 ± 0.036^ab^ | 0.101 ± 0.045^ab^ | 4.656 ± 0.373^c^ | 240.36 ± 173.66 |
|  | Median [95% CI] | 0.756 [0.728, 0.771] | 0.739 [0.716, 0.758] | 0.090 [0.075, 0.127] | 4.730 [4.614, 4.697] | 185.00 [140.09, 340.63] |
| **FROM PANONIA TO THE NORTH** | Mean ± SD | 0.761 ± 0.024^ab^ | 0.736 ± 0.032^ab^ | 0.097 ± 0.040^ab^ | 4.749 ± 0.327^c^ | 266.71 ± 288.75 |
|  | Median [95% CI] | 0.770 [0.739, 0.782] | 0.729 [0.707, 0.765] | 0.110 [0.060, 0.134] | 4.700 [4.696, 4.802] | 101.00 [0.00*, 533.76] |
| **FROM CENTRAL EUROPE TO NORTHWEST** | Mean ± SD | 0.698 ± 0.062^b^ | 0.685 ± 0.065^b^ | 0.184 ± 0.081^a^ | 4.170 ± 0.578^d^ | 209.75 ± 115.98 |
|  | Median [95% CI] | 0.710 [0.658, 0.738] | 0.693 [0.644, 0.726] | 0.175 [0.133, 0.236] | 4.240 [4.101, 4.238] | 235.50 [136.06, 283.44] |
| **FROM CENTRAL EUROPE TO WEST** | Mean ± SD | 0.768 ± 0.040^a^ | 0.757 ± 0.039^a^ | 0.083 ± 0.054^b^ | 4.886 ± 0.392^b^ | 363.69 ± 239.10 |
|  | Median [95% CI] | 0.775 [0.747, 0.789] | 0.766 [0.737, 0.778] | 0.070 [0.054, 0.111] | 4.910 [4.847, 4.925] | 293.00 [236.28, 491.10] |

Values are presented as Mean ± Standard Deviation (SD) followed by the Median and 95% Confidence Interval (CI) [Lower, Upper]. Different superscript letters (a, b, c, d) within the same column indicate statistically significant differences (p < 0.05) based on pairwise comparisons with Bonferroni correction. Lack of superscript letters in a column indicates no significant differences across any groups. ***H_o_***: observed heterozygosity; ***H_e_***: expected heterozygosity; ***F***: inbreeding coefficient; ***A_R_***: allelic richness; ***Ne_5_***: effective population size 5 generations ago (*SNeP* values). (*Note: The lower bound of the 95% CI for the 'From Panonia to the North' group's ***Ne_5_*** was mathematically calculated as -0.34 but is reported as 0.00, as population size cannot be negative).
